# The global burden of decubitus ulcers from 1990 to 2019

**DOI:** 10.1038/s41598-021-01188-4

**Published:** 2021-11-05

**Authors:** Xianghong Zhang, Na Zhu, Zhihong Li, Xiangtao Xie, Tang Liu, Guoqing Ouyang

**Affiliations:** 1 Department of Orthopedics, The Second Xiangya Hospital of Central South University, 139# Middle Renmin Road, Changsha, 410011 Hunan China; 2 Liuzhou People’s Hospital of Guangxi Medical University, Liuzhou, 545000 Guangxi China; 3 Centers for Disease Control and Prevention of Liuzhou, Liuzhou, 545000 Guangxi China

**Keywords:** Epidemiology, Health care, Skin diseases

## Abstract

There are no studies assessing the epidemiology and burden of decubitus ulcers at global, regional, and national levels. We aim to report this issue from 1990 to 2019 by extracting data from the Global Burden of Disease Study (GBD) 2019 and stratifying it by age, gender, and socio-demographic index (SDI). Globally, the number of prevalent cases of decubitus ulcers in 2019 is 0.85 (95% UI 0.78 to 0.94) million. The age-standardized rates of prevalence, incidence, and years lived with disability (YLDs) in 2019 are 11.3 (95% UI 10.2 to 12.5), 41.8 (37.8 to 46.2), and 1.7 (1.2 to 2.2) per 100,000 population, and compared with 1990, it has decreased by 10.6% (95% UI 8.7% to 12.3%), 10.2% (8.2 to 11.9%), and 10.4% (8.1 to 12.5%), respectively. In addition, the global prevalence rate of decubitus ulcers increases with age, peaking at the > 95 age group among men and women. At the regional and national levels, we observe a positive correlation between age-standardized YLDs and SDI. Malaysia, Saudi Arabia, and Thailand experienced the most significant increases in age-standardized prevalence rates at the national level. Finally, we concluded that the age-standardized prevalence, incidence, and YLDs rates of decubitus ulcer declined from 1990 to 2019, with significant regional differences. In order to monitor the dynamic changes of decubitus ulcers burden, it is recommended to improve the quality of decubitus ulcer health data in all regions and countries.

## Introduction

Despite the increase in medical knowledge and new effective prevention and treatments, decubitus ulcer is still a common and debilitating disease that presents a tremendous burden on the affected individuals, the healthcare systems, and socioeconomic costs^1–3^. A systematic review revealed that the incidence of decubitus ulcers is estimated to be approximately 12%^3^. The prevalence of decubitus ulcers in hospitalized patients in the United States is between 5 and 15%, and the prevalence in the intensive care unit is even higher^1, 4^. Recently, studies have only presented the burden of decubitus ulcers based on regional and/or national factors and have not provided comprehensive information in all countries and regions^1–4^.

To date, little is known about the estimates of cause-specific years lived with disability (YLDs) for decubitus ulcers at the global, regional, and national levels. In addition, published papers also failed to analyze the relationship between the burden of decubitus ulcers and the sociodemographic index (SDI) of each country. Moreover, no studies report the annual trends of the prevalence, incidence, and YLDs of decubitus ulcers over time for all countries globally. Therefore, considering the impact of decubitus ulcers on physical, social, and public health, it is important to understand the prevalence of decubitus ulcers and ensure that adequate resources are allocated for disease management and prevention.

In this study, in order to provide comprehensive and comparable information on the burden of decubitus ulcers, we analyzed data from the Global Burden of Disease (GBD) 2019 Study for global, regional, national incidence, prevalence, and YLDs in terms of counts and age-standardized rates (ASRs) by sex, age, and SDI. More detailed information about the burden of decubitus ulcers from different regions and countries may be beneficial for decision-makers to reduce the cost burden of decubitus ulcers.

## Methods

### Overview

The GBD study led by the Institute of Health Metrics and Evaluation (IHME) is the largest comprehensive study. In the recent update in 2019, the epidemiologic levels of 369 diseases and injuries, 282 causes of death, and 84 risk factors in 204 countries and territories, 21 regions, and 7 super-regions from 1990 to 2019 are analyzed^5^. We obtained the data from the Global Health Data Exchange query toll (http://ghdx.healthdata.org/gbd-results-tool), including prevalence, incidence, and YLDs. The general methodology of the GBD 2019 study and its latest updates compared with previous years has been explained in the previous GBD 2019 publication^6^. Our present study complies with the Guidelines for Accurate and Transparent Health Estimates Reporting (GATHER) statement^7^.

### Case definition and data sources

In the GBD 2019 study, decubitus ulcers are defined as an injury to the skin and underlying tissue resulting from an obstruction of blood flow due to pressure on the skin. It is also known as a pressure ulcer/sore^5^. The database in the GBD 2010 study of decubitus ulcers included surveys and systematic reviews in PubMed and Google Scholar with stipulated inclusion criteria. For the GBD 2013 study, the search strategy of the GBD 2010 study was replicated to capture epidemiological studies published between 2012 and 2013. Subsequently, other studies that met the inclusion criteria, hospital inpatient, USA claims data from 2010 to 2016, Taiwan claims data for 2016, and Poland claims data for 2015–2017, were added during the GBD 2019 study update. The exclusion criteria include abnormal or unreasonable data compared with the regional, super-regional, and global ratios. Details on data adjustment were consistent with previous studies^5^. Data obtained from the GBD 2019 study for decubitus ulcers were employed to calculate the study’s estimates. The detail data sources used in estimating the burden of decubitus ulcers in the different countries can be found with the GBD 2019 Data Input Sources Tool using the following link: http://ghdx.healthdata.org/gbd-2019/data-input-sources5.

### Data processing and disease modeling

The data processing described here was carried out by the team at IHME^5^. Using a Bayesian meta-regression tool and DisMod-MR 2.1, to analyze the decubitus ulcers’ incidence and prevalence data by pooling the available heterogeneous data. DisMod-MR 2.1 can perform age-integration; however, its effect will be reduced when integrating across wide age groups (for example, all age groups). To solve this problem, the data run by the DisMod-MR 2.1 model was segmented by age to calculate the age-pattern of countries, and then the calculated age-pattern was applied to split all the aggregated age data^5^.

For decubitus ulcers in the GBD 2019 study, the separate global and data-rich models were hybridized to acquire the unadjusted results. The previous within-DisMod crosswalks were changed to crosswalked completed using the MR-BRT modeling tool. To better represent the general population, the reference incidence data was adjusted with inpatient data, along with USA market scan data 2000 and inpatient data toward the level of other incidence data points. To guide estimates for locations with few or no data, log-transformed lagged distributed income per capital (LDI) was used as a country-level covariate, and LDI was restricted to a range of -0.5 to -0.1. In addition, the location random effects were restricted to (-0.5 and 0.5) across all 7 GBD super-regions. In previous rounds, the prior excess mortality rate (EMR) was estimated in DisMod by matching prevalence data points with their corresponding cause-specific mortality rate (CSMR) values within the same age, sex, year, and location. The corresponding prevalence was derived by running an initial model and then applying the same CSMR/prevalence method for short duration conditions (remission > 1). However, for many causes, DisMod estimated an unrealistic model of EMR compared to the expected pattern decreasing EMR with greater access to quality health care. In order to provide greater guidance to DisMod on the expected pattern of excess EMR, the MR-BRT approach using age and sex with a prior on healthcare access and quality index (HAQi) having a negative coefficient was applied to model the EMR data generated in the previous round. The results from MR-BRT were applied to predicted for each location year, sex and for ages 0, 10, 20…100^5^.

### Severity and YLDs

The International Classification of Diseases (ICD) version 9 code for decubitus ulcers is 707, and the ICD-10 codes are L89-L89.95. Three disease severity levels (sequelae) were estimated, with disability weights (DWs) considered as a weight factor^5^. Furthermore, disfigurement and level 1 with itch/pain, disfigurement and level 2 with itch/pain, and disfigurement and level 3 with itch/pain are defined as mild decubitus ulcers, moderate decubitus ulcers, and severe decubitus ulcers, respectively. The DWs associated with these severities are manifested as mild decubitus ulcers 0.027 (0.015–0.042), moderate decubitus ulcers 0.188 (0.124–0.267), and severe decubitus ulcers 0.576 (0.401–0.731). The overall prevalence of decubitus ulcers was classified into severity categories by applying the disability weight and disfigurement with itch/pain. Finally, the prevalence of severity in each category was multiplied by a severity-specific disability weight to calculate the YLDs^5^.

### Complication of results

Using a bootstrap method, uncertainty is caused by performing 1000 ordered draws at each computational step, incorporating the uncertainty from multiple sources, such as input data, corrections of measurement errors, and estimates of residual non-sampling errors. All of the estimates were reported along with their uncertainty intervals (UIs) at IHME. The 95% UIs were determined according to the 2.5th and 97.5th percentile of the ordered draws.

Our team created smoothing spline models to examine the shape of the correlation curve between the burden of decubitus ulcer in terms of YLDs and SDI for 21 regions and 204 countries and territories. SDI is a composite indicator of lag-distributed income per capita and was calculated from the gross domestic product per capita, the total fertility rate among those < 25 years old, and the average years of schooling for the population > 15 years old. The value of SDI ranges from 0 (less developed) to 1 (most developed). All statistics were generated by R software version 3.6.3 (https://cran.r-project.org/doc/FAQ/R-FAQ.html#Citing-R) and visualized using the ggplot2 3.3.0 package^8^. An unpaired *t* test was adopted to analyze the differences between sexes, and it is considered statistically significant if *P* value < 0.05.

## Results

### Prevalence of decubitus ulcer

Globally, the number of prevalent cases of decubitus ulcer was 0.42 (95% UI 0.38 to 0.46) million in 1990, with an age-standardized prevalence rate of 12.6 (11.3 to 14.0) per 100,000 population. In 2019, 0.85 (95% UI 0.78 to 0.94) million prevalent cases of decubitus ulcers were identified, with an age-standardized point prevalence estimate (per 100,000 population) of 11.3 (95% UI 10.2 to 12.5). The age-standardized prevalence rate decreased 10.6% (95% UI 8.7 to 12.3%) from 1990 to 2019 (Table 1 and Table S1).

**Table 1:** Prevalent cases, incident cases, and years lived with disability (YLDs) for decubitus ulcer in 2019 for both sexes and percentage change of age-standardized rates (ASR) per 100,000 populations from 1990 to 2019 by Global Burden of Disease regions.

Different regions	Prevalence (95% uncertainty interval)			Incidence (95% uncertainty interval)			YLDs (95% uncertainty interval)		
	Counts	ASR per 100,000 population (95% UI)	Percentage change in ASRs per 100,000 population (95% UI)	Counts	ASR per 100,000 population (95% UI)	Percentage change in ASRs per 100,000 population (95% UI)	Counts	ASR per 100,000 population (95% UI)	Percentage change in ASRs per 100,000 population (95% UI)
Global	853,854 (776,189 to 942,491)	11.3 (10.19 to 12.48)	− 0.1 (− 0.12 to − 0.09)	3,170,796 (2,875,433 to 3,499,729)	41.8 (37.8 to 46.22)	− 0.1 (− 0.12 to − 0.08)	130,238 (92,478 to 171,036)	1.7 (1.21 to 2.24)	− 0.1 (− 0.12 to − 0.08)
Andean Latin America	4817 (4323 to 5338)	8.7 (7.79 to 9.63)	0.2 (0.18 to 0.25)	17,942 (16,165 to 19,885)	32.4 (29.22 to 35.93)	0.2 (0.18 to 0.26)	778 (535 to 1060)	1.4 (0.96 to 1.9)	0.2 (0.07 to 0.31)
Australasia	6434 (5662 to 7254)	12.5 (11.1 to 13.98)	− 0.1 (− 0.14 to − 0.05)	23,895 (20,996 to 27,058)	46.3 (41.13 to 51.74)	− 0.1 (− 0.14 to − 0.05)	961 (663 to 1301)	1.9 (1.32 to 2.58)	− 0.1 (− 0.2 to 0.04)
Caribbean	11,192 (10,142 to 12,348)	21.7 (19.63 to 23.88)	0.1 (0.09 to 0.16)	41,506 (37,440 to 45,961)	80.3 (72.4 to 88.88)	0.1 (0.1 to 0.16)	1711 (1203 to 2282)	3.3 (2.33 to 4.41)	0.1 (0.04 to 0.2)
Central Asia	1055 (918 to 1213)	1.6 (1.39 to 1.8)	0.1 (0.07 to 0.11)	3862 (3378 to 4406)	5.8 (5.12 to 6.59)	0.1 (0.06 to 0.1)	187 (125 to 255)	0.3 (0.19 to 0.38)	0.1 (0.06 to 0.11)
Central Europe	24,606 (22,180 to 27,197)	12 (10.86 to 13.12)	0.3 (0.23 to 0.33)	91,933 (83,069 to 101,445)	44.6 (40.73 to 48.85)	0.3 (0.23 to 0.33)	3785 (2650 to 5013)	1.9 (1.3 to 2.49)	0.2 (0.19 to 0.32)
Central Latin America	61,804 (55,697 to 68,479)	27.4 (24.63 to 30.44)	0 (0 to 0.04)	231,151 (208,858 to 255,964)	102.7 (92.34 to 114.01)	0 (0 to 0.05)	9436 (6588 to 12,481)	4.2 (2.92 to 5.5)	0 (− 0.02 to 0.07)
Central Sub-Saharan Africa	2823 (2471 to 3202)	4.2 (3.79 to 4.67)	0 (0 to 0.04)	10,366 (9107 to 11,680)	15.7 (14.14 to 17.42)	0 (0 to 0.04)	496 (336 to 684)	0.7 (0.49 to 0.97)	0 (− 0.05 to 0.09)
East Asia	92,666 (82,463 to 104,013)	5.7 (5.05 to 6.39)	0.3 (0.2 to 0.34)	350,449 (311,845 to 394,816)	21.4 (18.99 to 24.15)	0.3 (0.22 to 0.35)	14,633 (10,148 to 19,455)	0.9 (0.61 to 1.16)	0.2 (0.14 to 0.29)
Eastern Europe	24,797 (21,959 to 27,988)	7.8 (6.99 to 8.74)	0.1 (0.06 to 0.08)	90,580 (80,446 to 102,015)	28.5 (25.55 to 31.91)	0.1 (0.05 to 0.08)	4065 (2794 to 5511)	1.3 (0.89 to 1.75)	0.1 (0.02 to 0.11)
Eastern Sub-Saharan Africa	9107 (7930 to 10,376)	4 (3.58 to 4.54)	0 (0.03 to 0.05)	33,309 (29,098 to 37,929)	14.8 (13.27 to 16.7)	0 (0.03 to 0.05)	1612 (1096 to 2229)	0.7 (0.48 to 0.97)	0 (0.03 to 0.05)
High-income Asia Pacific	61,393 (54,376 to 69,977)	13.6 (12.27 to 15.03)	0 (− 0.04 to 0.01)	226,243 (199,724 to 258,461)	49.9 (45.11 to 55.4)	0 (− 0.04 to 0.01)	9408 (6611 to 12,710)	2.2 (1.51 to 2.95)	0 (− 0.04 to 0.03)
High-income North America	221,138 (202,795 to 240,434)	34.6 (31.91 to 37.56)	− 0.1 (− 0.14 to -0.06)	819,080 (752,601 to 886,065)	128 (118.5 to 138.24)	− 0.1 (− 0.14 to − 0.06)	31,877 (22,897 to 41,435)	5.1 (3.66 to 6.64)	− 0.1 (− 0.15 to − 0.06)
North Africa and Middle East	15,697 (14,099 to 17,652)	4.2 (3.78 to 4.77)	0.3 (0.26 to 0.34)	58,384 (52,568 to 65,387)	15.9 (14.15 to 17.85)	0.3 (0.25 to 0.34)	2606 (1799 to 3505)	0.7 (0.48 to 0.92)	0.3 (0.18 to 0.33)
Oceania	242 (217 to 271)	5.3 (4.74 to 5.97)	0.3 (0.26 to 0.33)	913 (818 to 1020)	20.2 (17.91 to 22.7)	0.3 (0.25 to 0.32)	39 (27 to 52)	0.8 (0.57 to 1.08)	0.3 (0.14 to 0.41)
South Asia	22,764 (20,060 to 25,884)	1.8 (1.62 to 2.07)	0.3 (0.29 to 0.36)	85,350 (75,361 to 97,351)	6.9 (6.16 to 7.82)	0.3 (0.29 to 0.36)	3969 (2664 to 5405)	0.3 (0.21 to 0.42)	0.3 (0.25 to 0.32)
Southeast Asia	32,237 (29,199 to 35,988)	6.7 (6.06 to 7.55)	0.6 (0.6 to 0.71)	120,615 (108,476 to 134,340)	25.3 (22.68 to 28.39)	0.6 (0.58 to 0.68)	5092 (3532 to 6741)	1 (0.72 to 1.38)	0.6 (0.5 to 0.73)
Southern Latin America	17,511 (15,470 to 19,821)	20.4 (18.05 to 23.02)	0.6 (0.48 to 0.69)	64,975 (57,404 to 73,761)	75.6 (66.85 to 85.59)	0.6 (0.46 to 0.66)	2611 (1837 to 3541)	3 (2.14 to 4.12)	0.5 (0.34 to 0.77)
Southern Sub-Saharan Africa	6755 (6167 to 7423)	13.2 (11.93 to 14.58)	0.1 (0.05 to 0.08)	25,327 (23,154 to 27,929)	49.9 (44.78 to 55.34)	0.1 (0.04 to 0.08)	1081 (758 to 1441)	2 (1.44 to 2.7)	0.1 (0 to 0.11)
Tropical Latin America	56,254 (51,230 to 62,092)	24.3 (22.15 to 26.82)	0.2 (0.13 to 0.19)	210,101 (191,663 to 230,778)	90.8 (82.65 to 99.99)	0.2 (0.14 to 0.2)	8695 (6076 to 11,543)	3.7 (2.62 to 4.96)	0.2 (0.09 to 0.22)
Western Europe	168,939 (150,376 to 190,360)	17.2 (15.54 to 19.09)	− 0.1 (− 0.08 to − 0.02)	622,022 (551,286 to 704,091)	63.2 (57.05 to 70.32)	− 0.1 (− 0.07 to − 0.02)	25,199 (17,931 to 33,741)	2.7 (1.88 to 3.54)	0 (− 0.09 to 0)
Western Sub-Saharan Africa	11,622 (10,285 to 13,029)	5.4 (4.9 to 6.03)	0.2 (0.19 to 0.25)	42,793 (38,134 to 47,615)	20.4 (18.42 to 22.58)	0.2 (0.19 to 0.25)	1996 (1357 to 2734)	0.9 (0.62 to 1.21)	0.2 (0.15 to 0.22)

**Figure 1:**
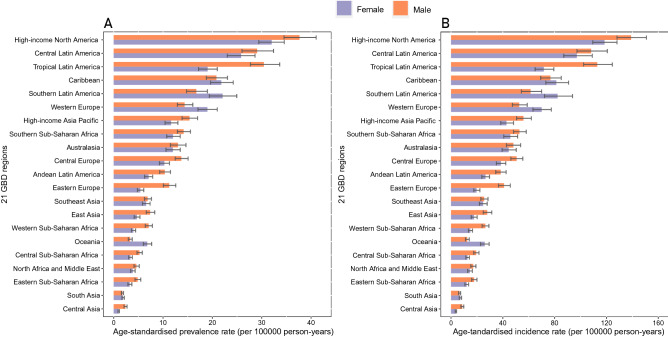
The age-standardized rate of decubitus ulcers in 2019 for 21 GBD regions, by sex. (**A**) The age-standardized prevalence rate in 2019; (**B**) The age-standardized incidence rate in 2019.

At the regional level, the highest age-standardized prevalence rates of decubitus ulcers per 100,000 population in 2019 were observed in high-income North America (34.6 [31.9 to 37.6]), Central Latin America (27.4 [24.6 to 30.4]), and Tropical Latin America (24.3 [22.2 to 26.8]) (Table 1 and Fig. 1A). The percentage change in age-standardized prevalence rates of decubitus ulcers from 1990 to 2019 varied across regions. While all regions except four (high-income Asia Pacific, Western Europe, Australasia, and High-income North America) showed an increasing trend between 1990 and 2019, the highest was observed in Southeast Asia (64.9% [59.6 to 71.1%]), Southern Latin America (57.0% [47.5 to 68.6%]), and South Asia (32.1% [28.6 to 35.5%]) (Table 1 and Fig. S1A). Similarly, we found that the contribution to the number of prevalent cases differed by approximately 900 times among the 21 GBD regions in 2019. High-income North America (0.22 [0.20 to 0.24] million), Western Europe (0.17 [0.15 to 0.19] million), and East Asia (0.09 [0.08 to 0.10] million) reported the largest number of prevalent cases in 2019 (Table 1 and Fig. S2).

**Figure 2:**
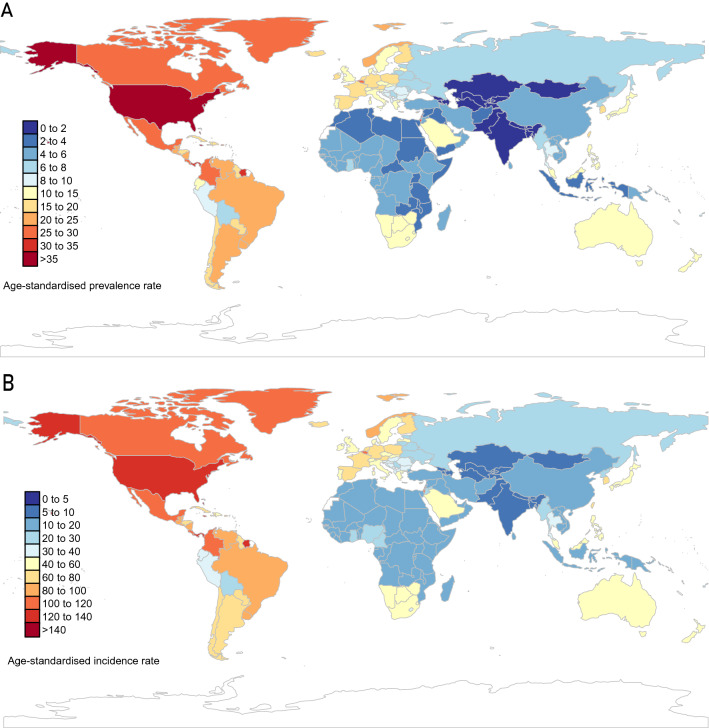
The global age-standardized prevalence (**A**) and incidence rate (**B**) of decubitus ulcers per 100,000 populations in 2019, by country and territory. These pictures were generated by R software version 3.6.3 (https://cran.r-project.org/doc/FAQ/R-FAQ.html#Citing-R) and visualized using the ggplot2 3.3.0 package^8^.

In 2019, the age-standardized prevalence rates of decubitus ulcers ranged from 1.5 to 55.2 cases per 100,000 population at the national level. Countries with the highest age-standardized prevalence rates included Barbados (55.2 [50.1 to 61.4]), Bahamas (40.6 [36.5 to 45.8]), and Saint Kitts and Nevis (39.7 [35.5 to 44.7]). (Table S1 and Fig. 2A). Percentage change in age-standardized prevalence rates from 1990 to 2019 differed substantially between countries and territories. The most significant increases were seen in Malaysia (110.3% [97.3 to 123.6%]), Saudi Arabia (100.1% [88.4 to 112.5%]), and Thailand (81.5% [69.4 to 96.5%]). In contrast, the largest decreasing trends were seen in France (− 22.7% [− 27.7 to − 17.0%]), Ireland (− 21.1% [− 26.5 to − 15.2%]), and Italy (− 20.8% [− 23.4 to − 18.1%]) (Table S1 and Fig. S3A). The United States of America (0.2 [0.18 to 0.22] million) reported the largest number of prevalent cases in 2019.

### Incidence of decubitus ulcer

Globally, decubitus ulcers accounted for 1,541,945 (1,389,163 to 1,720,928) incident cases in 1990 and 3,170,796 (2,875,433 to 3,499,729) new cases in 2019, with an age-standardized incidence rate of 41.8 (37.8 to 46.2) in 2019 per 100,000 population; this rate decreased by 10.2% (8.2–11.9%) since 1990 (Table S2 and Table 1).

At the regional level, regions with the highest age-standardized incidence rates of decubitus ulcers per 100,000 persons in 2019 were also high-income North America (128.0 [118.5 to 138.2]), Central Latin America (102.7 [92.3 to 114.0]), and Tropical Latin America (90.8 [82.6 to 100.0]) (Table 1 and Fig. 1B). From 1990 to 2019, the percentage change in the age-standardized incidence rates was highest in Southeast Asia (62.3% [57.7 to 68.2%]), Southern Latin America (55.3% [45.7 to 65.7%]), and South Asia (32.6% [29.1 to 36.0%]), and again there were four regions (High-income Asia Pacific, Western Europe, Australasia, and high-income North America) with a decreasing trend (Table 1 and Fig. S1B). In addition, the highest number of incident cases in 2019 was also found in the same regions of prevalent cases, including high-income North America (819,080 [752,601 to 886,065]), Western Europe (622,022 [551,286 to 704,091]), and East Asia (350,449 [311,845 to 394,816]) (Table 1 and Fig. S4).

Age-standardized incidence rates of decubitus ulcers in 2019 ranged from 5.6 to 198.4 cases per 100,000 population. In 2019, the highest age-standardized incidence rates occurred in Barbados (198.4 [178.8 to 220.7]), Bahamas (147.2 [131.8 to 167.0]), and Saint Kitts and Nevis (145.1 [130.0 to 163.7]). (Table S2 and Fig. 2B). The largest increase in age-standardized incidence rate of decubitus ulcers from 1990 to 2019 occurred in Malaysia (105.7% [94.8 to 117.2%]), Saudi Arabia (97.4% [87.0 to 107.9%]), and Thailand (77.6% [66.2 to 92.7%]), whereas the largest decreases during the same period occurred in France (− 21.6% [− 27.2 to − 15.9%]), Italy (− 20.5% [− 23.3 to − 17.7%]), and Ireland (− 20.3% [− 25.5 to − 14.2%]) (Table S2 and Fig. S3B). The United States of America (744,426 [686,418 to 804,927]) reported the greatest number of incident cases in 2019.

### YLDs of decubitus ulcer

Globally, decubitus ulcers YLDs amounted to 64,857 (45,375 to 85,485) in 1990 and 130,237 (92,478 to 171,036) in 2019, with an age-standardized YLDs rate of 1.9 (1.4 to 2.5) in 1990 and 1.7 (1.2 to 2.2) in 2019 per 100,000 population, this rate decreased by 10.4% (8.1 to 12.5%) from 1990 to 2019 (Table S3 and Table 1).

High-income North America (5.1 [3.7 to 6.6]), Central Latin America (4.2 [2.9 to 5.5]), and Tropical Latin America (3.7 [2.6 to 5.0]) were found to have the highest age-standardized YLDs’ rate of decubitus ulcers per 100,000 population in 2019, while the regions with the lowest rates were Central Asia (0.3 [0.2 to 0.4]), South Asia (0.3 [0.2 to 0.4]), north Africa and the Middle East (0.7 [0.5 to 0.9]) (Table 1 and Fig. S5). In addition, the regions with the largest increases in the age-standardized YLDs’ rates between 1990 and 2019 were Southeast Asia (59.8% [49.9 to 72.6%]), Southern Latin America (53.1% [34.0 to 76.6%]), and South Asia (28.3% [24.9 to 32.2%]) (Table 1 and Fig. S6).

At the national level, the age-standardized YLDs’ rate ranged from 0.3 years to 8.4 years per 100,000 population in 2019. The countries with the highest age-standardized prevalence rates of decubitus ulcers in 2019 also showed the highest age-standardized YLDs rate (Table S3 and Fig. S7). The most significant increases in age-standardized YLDs rates between 1990 and 2019 were observed in Malaysia (105.7% [70.4 to 148.1%]), Saudi Arabia (93.1% [59.0 to 134.1%]), and Thailand (78.6% [44.8 to 124.2%]), while the countries with the most significant decreases in age-standardized YLDs rates were France (− 21.2% [− 31.6 to − 9.7%]), Ireland (− 20.1% [− 31.5 to − 6.7%]), and Italy (− 19.9% [− 24.4 to − 15.1%]) (Table S3 and Fig. S8).

### Age and sex patterns

**Figure 3:**
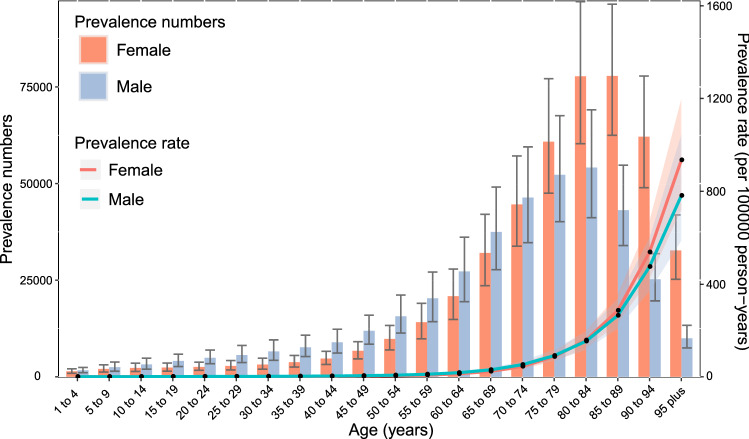
Global number of prevalence cases and prevalence rates of decubitus ulcers per 100,000 populations by age and sex, 2019, Shading indicate the 95% upper and lower uncertainty intervals (95% UIs) for the prevalent rate, respectively.

Among the incidence, prevalence, and YLDs of decubitus ulcers, we have employed different patterns according to age and sex. Globally, age-standardized prevalence rates increased with age, peaking at the > 95 age group for both men and women in 2019. However, the number of prevalent cases increased with age and peaked in the 80–84 years age group and 85–89 years age group for males and females, respectively, with decreasing patterns observed in the older age groups (Fig. 3). In 2019, the global incidence rate was also found to increase with age, peaking at the > 95 age group for both females and males. The number of incident cases reached the highest level at the 80–84 age group and the 85–89 age group for males and females, respectively. A decreasing trend in the number of incident cases was observed after that. Under the age of 75 years, the number of incident cases was also higher in males, whereas the number of incident cases was lower in males than females in the 75 years and older age group (Fig. S9). Furthermore, patterns of YLDs rates and numbers by sex across the age groups were relatively similar to those of the prevalence patterns (Fig. S10). Notably, there were no statistically significant differences between men and women for prevalence, incidence, and YLDs in all age groups (*P* > 0.05).

### Burden of decubitus ulcer by SDI

**Figure 4:**
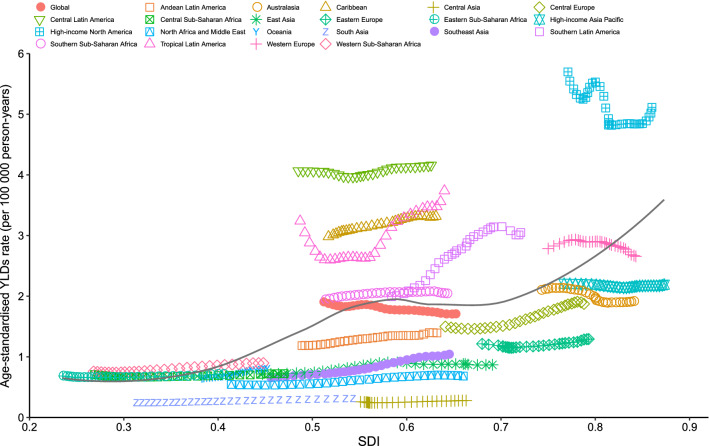
Association of age-standardized YLDs rate due to decubitus ulcers and SDI for 21 regions in the GBD study. Expected values based on SDI and disease rates in all locations are shown as the black line. Different points are plotted for each GBD region and show observed age-standardized YLDs rates from 1990 to 2019.

Generally, a positive association between the age-standardized YLDs rate of decubitus ulcer and the SDI at the global level and for all GBD regions was observed. Globally, the observed burden of decubitus ulcer was higher than the expected level in patients from regions with lower SDI. However, for patients from regions with higher SDI, the observed burden of decubitus ulcer was lower than the expected level. At the regional level, the observed burden estimate of decubitus ulcer was higher than the expected level based on the SDI from 1990 to 2019 for high-income North America, Central Latin America, the Caribbean, and Tropical Latin America. However, Central Europe, Andean Latin America, Eastern Europe, East Asia, North Africa and the Middle East, South Asia, and Central Asia had a lower-than-expected burden of decubitus ulcers during all measurement periods (Fig. 4).

**Figure 5:**
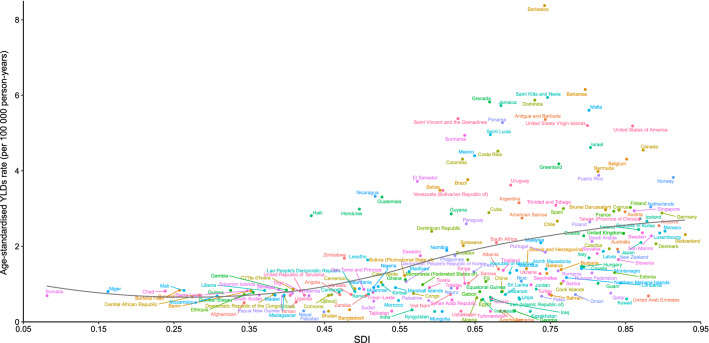
Association of age-standardized YLDs rate due to decubitus ulcers and SDI for 204 countries and territories. Expected values based on SDI and disease rates in all locations are shown as the black line. Each point shows the observed YLDs rate per country in 2019.

A generally positive correlation between age-standardized YLDs rates and SDI of decubitus ulcers in 2019 was also noted at the national level. Barbados, Bahamas, Saint Kitts and Nevis, United States of America, Canada, Norway, France, Finland, and many other countries or territories showed much higher burden of decubitus ulcers than the expected level of age-standardized YLDs rates based on SDI. In contrast, Mongolia, India, China, Japan, Australia, United Kingdom, and many other countries or territories showed a much lower than expected age-standardized YLDs rates (Fig. 5). Similarly, there were also generally positive associations between the SDI and age-standardized incidence and prevalence rates of decubitus ulcers in 2019 (Fig. S11–S14).

## Discussion

To the best of our knowledge, the prevalence, incidence, and YLDs numbers and ASRs for decubitus ulcers in 204 counties and territories from 1990 to 2019 are the first to be reported in this study. Globally, decubitus ulcers accounted for 0.85 million prevalent cases, 3.17 million incident cases, and 0.13 million YLDs in 2019. The ASRs of prevalence, incidence, and YLDs for decubitus ulcers was 11.3, 41.8, and 1.7 per 100,000 population, respectively. High-income North America, Central Latin America, and Tropical Latin America were found to have the highest point prevalence estimates.

A previous systematic review of 79 studies of decubitus ulcer reported that the estimated prevalence was 10.8% (range 4.6% to 27.2%) in Europe^9^. Moreover, another systematic review concluded that the estimated incidence rate of decubitus ulcers was approximately 12%^3^. Based on the GBD 2017 study, the estimated incidence rate of the European 15 + countries ranged from 40.7 to 139.4 cases per 100,000 population-years^10^. The above studies presented the prevalence and incidence of decubitus ulcers for only some selected regions and countries. In the present study, the age-standardized prevalence and incidence rates for decubitus ulcers at a global level were 11.3 (95% CI 10.2 to 12.5) and 41.8 (95% CI 37.8 to 46.2) per 100,000 population-years in 2019, respectively. The largest age-standardized prevalence and incidence rates were found in Barbados. Due to differences in the methodologies and data sources, the results of the above studies could not be directly compared with our results. Additionally, the above systematic reviews did not present age-standardized incidence, prevalence, and YLDs rates of decubitus ulcers for both sexes and separately. However, the age-standardized strategy might affect the results if the population of the regions or countries is relatively young. Moreover, the input data of decubitus ulcers were incidence from hospital admission and outpatient data for the GBD study^11^.

The formulation of accurate, transparent, and frequently updated disease burden measures may affect different levels of healthcare and further influence the role of dermatology in global health. We found that in this study, the percent changes in the age-standardized prevalence, incidence, and YLDs rate of decubitus ulcers were reduced from 1990 to 2019, indicating that the global burden of decubitus ulcers decreased with time within the GBD 2019 study. Similar results were also presented in the previous GBD 2013 study^12^. However, a recent study demonstrated a general trend for increasing age-standardized incidence rates across European 15 + countries of the GBD 2017 study from 1990 to 2017^10^. Similarly, high-income North America, Central Latin America, and Tropical Latin America had the largest prevalence and incidence rates among the 21 GBD regions. However, intrinsic and extrinsic risk factors may be the primary reasons for the high burden of decubitus ulcers within these regions^4^. Moreover, the aging population with an increase in the elderly living with disability, the corresponding rates of decubitus ulcers continue to grow. Therefore, a continuous emphasis on prevention measures, management, and treatment may be prioritized by policy makers. Additionally, more attention should also be paid to the regional prevalence and incidence of decubitus ulcers.

There were no statistically significant differences between males and females in the present study regarding prevalence, incidence, and YLDs of decubitus ulcers. Similar to our results, some previous studies also reported that sex cannot be regarded as an independent risk factor for decubitus ulcers development, and the differences regarding decubitus ulcers prevention interventions seem to be minimal^4, 13^. However, some studies have shown that the prevalence of decubitus ulcers in men may be higher than that in women^10, 14^. According to their opinions, smoking was more common in men than women. The limited data sources and different methodologies may reduce the reliability of their above conclusions. Additionally, the distribution of age varied greatly in this study. In the GBD 2019 study, the highest burden of decubitus ulcer was found to increase with age, peaking at more than 95 years of age for both males and females. This means that there should be more policies for these specific age groups globally. Although the burden of decubitus ulcers in younger people was much lower than that of the middle-aged and older-aged groups, this does not mean that adolescent decubitus ulcer does not need attention as well as additional prevention and management measures.

Wide geopolitical variation is also characteristic of decubitus ulcers and may be attributed to multiple factors. For example, there appears to be some association in the prevalence of decubitus ulcers and climate, environmental factors, armed conflict, and others^4^. Additionally, due to the availability and quality of available diagnostics and treatments, socioeconomic factors affect the disease burden of decubitus ulcers. However, the relationship between the level of a country’s development and the incidence of decubitus ulcer remains poorly understood; thus, our present study revealed some important findings. There is a positive correction between YLDs and SDI in 21 GBD regions and 204 countries and territories regarding decubitus ulcers from 1990 to 2019. This means that the countries with higher socioeconomic development levels generally have a higher burden of decubitus ulcers. Low smoking rates due to shortages of tobacco and low population aging levels may be the reasons for the lower burden of decubitus ulcers in lower SDI countries and territories. Alternatively, this phenomenon could also be attributed to prolonged immobility due to higher levels of disabling diseases in higher SDI countries. However, the high burden of decubitus ulcer is not limited to high-SDI or low-SDI regions and countries, which shows that decubitus ulcer is not just a health problem in high income countries. In some regions such as high-income North America, Central Latin America, Caribbean, and Tropical Latin America, as well as some countries and territories such as Barbados, Bahamas, United States of America, Canada, France, and Finland, the burden of decubitus ulcer was higher than the expected levels. However, the global burden of decubitus ulcers has been lower than the expected levels in recent years. This global phenomenon could be attributed to effective preventive measures, early diagnosis, improved health care, and clarity on the optimal timing to conduct different effective interventions (antibiotics, dressing, biophysical modalities, surgery)^4^. However, the burden of decubitus ulcer could be estimated relative to the expected burden based on the SDI when considering prevention programs.

The importance of decubitus ulcer, its consequences, and treatment strategies may not be apparent for most diagnosed patients. This highlights the need for prevention programs to further public health awareness of decubitus ulcers disease onset, risk factors, consequences, and treatment strategies, which may require health education interventions. To develop these programs and interventions, the emphasis should be placed on the risk factors associated with the occurrence of decubitus ulcers, as reported in the literature, including intrinsic and extrinsic risk factors^4^. Alleviating the risk factors for the individual patients will prevent the formation of decubitus ulcers, for example, minimizing episodes of prolonged pressure by placing appropriate padding at pressure points or frequently repositioning the patients. Furthermore, adequate nutrition is also vital in preventing decubitus ulcers formation^15^. Despite a multifaceted approach that could have the potential to decrease the disease burden caused by decubitus ulcers; thus, the prevention and treatment, remains frustrating and time-consuming. Therefore, it is necessary to focus on the systemic improvement of decubitus ulcer care, including more effective prevention and treatment.

This study comprehensively analyzed the relative burden of decubitus ulcers globally, regionally, and nationally from 1990 to 2019; however, there are important limitations to consider. First, the accuracy and robustness of the GBD 2019 study estimates may be affected by the quality and quantity of the input data used in the DisMod-MR 2.1 model. Due to data deficiencies and sparsity in many regions and countries, only a few countries globally provided actual national data. The burden estimates heavily rely on the modeled data rather than the typical data based on individual population studies. Therefore, we should explain the burden of decubitus ulcers more carefully at a national level. In order to obtain more detailed information and representative data from each country and territory, national health surveys were encouraged if possible. Second, using coding systems other than ICD in primary care may decrease the availability of data. Third, no country-level covariates were used due to the limited knowledge of risk factors. Finally, geographical coverage may affect the available studies for comparisons among the different GBD regions where certain populations have a relative excess or scarcity of studies in relation to their total population^16, 17^. Despite the inherent limitations of the GBD study and the global reporting burden of decubitus ulcers, large-scale epidemiological data continues to help key policy makers formulate public health policies.

Decubitus ulcers are a major public health challenge with variations between countries regarding the burden of decubitus ulcers. At a global level, the age-standardized prevalence, incidence, and YLDs rates decreased from 1990 to 2019. The highest burden of decubitus ulcers was observed in elderly-aged patients due to the ageing of the global population. There were no statistically significant differences for prevalence, incidence, and YLDs of decubitus ulcers between males and females. To mitigate the future burden of decubitus ulcers improved awareness, early diagnosis, effective treatment, improved healthcare infrastructure, and health conditions are warranted. Furthermore, improving the population-based health data of decubitus ulcers at the national level as well as the dynamic monitoring of the decubitus ulcer burden provides further prevention and treatment strategies for decubitus ulcers.

## Supplementary Information

Supplementary Tables.

Supplementary Figures.

## Data Availability

The datasets generated for this study can be found in the GBD at http://ghdx.healthdata.org/gbd-results-tool. All data used in this study is publicly available.
