# Supplementary material for: The global burden of decubitus ulcers from 1990 to 2019
Source: Sci Rep. 2021 Nov 5;11:21750. doi: 10.1038/s41598-021-01188-4 (PMC8571371; doi:10.1038/s41598-021-01188-4)
Supplement: Supplementary file 1 — Supplementary Tables. [file 41598_2021_1188_MOESM1_ESM.docx]

**Additional file 1**

**The global burden of decubitus ulcers from 1990 to 2019**

**List of supplemental Table**

**Table S1**: Prevalent cases of decubitus ulcer in 1990 and 2019 for both sexes and percentage change of age-standardized rates (ASR) by location…………………………………........2-13

**Table S2**: Incident cases of decubitus ulcer in 1990 and 2019 for both sexes and percentage change of age-standardized rates (ASR) by location……………………………………...14-26

**Table S3**: YLDs of decubitus ulcer in 1990 and 2019 for both sexes and percentage change of age-standardized rates (ASR) by location………………………………………………...27-38

**Table S1: Prevalent cases of decubitus ulcer in 1990 and 2019 for both sexes and percentage change of age-standardized rates (ASR) by location.**

| **Regions or countries** | **1990** | | **2019** | | **Percentage change in ASR from 1990 and 2019** |
| --- | --- | --- | --- | --- | --- |
|  | **Counts (95% UI)** | **Rate (95% UI)** | **Counts (95% UI)** | **Rate (95% UI)** |  |
| Global | 417024(375180 to 462607) | 12.6(11.33 to 14.05) | 853854(776189 to 942491) | 11.3(10.19 to 12.48) | -0.1(-0.12 to -0.09) |
| High-income North America | 138741(124275 to 154182) | 38.4(34.66 to 42.51) | 221138(202795 to 240434) | 34.6(31.91 to 37.56) | -0.1(-0.14 to -0.06) |
| Canada | 9776(8794 to 10896) | 31.2(28.14 to 34.68) | 19998(17709 to 22659) | 29.5(26.39 to 33.12) | -0.1(-0.1 to -0.01) |
| Greenland | 9(8 to 10) | 26.2(23.1 to 29.34) | 16(14 to 19) | 27.4(24.19 to 31.03) | 0(0.01 to 0.08) |
| United States of America | 128953(115285 to 143353) | 39.1(35.23 to 43.27) | 201120(184624 to 218008) | 35.3(32.54 to 38.12) | -0.1(-0.14 to -0.06) |
| Australasia | 3011(2690 to 3346) | 13.8(12.42 to 15.28) | 6434(5662 to 7254) | 12.5(11.1 to 13.98) | -0.1(-0.14 to -0.05) |
| Australia | 2523(2254 to 2805) | 14(12.55 to 15.43) | 5488(4807 to 6206) | 12.6(11.15 to 14.13) | -0.1(-0.15 to -0.05) |
| New Zealand | 488(431 to 548) | 13.2(11.65 to 14.74) | 946(843 to 1058) | 12(10.74 to 13.3) | -0.1(-0.14 to -0.05) |
| High-income Asia Pacific | 24491(21882 to 27354) | 13.8(12.36 to 15.44) | 61393(54376 to 69977) | 13.6(12.27 to 15.03) | 0(-0.04 to 0.01) |
| Brunei Darussalam | 18(16 to 20) | 17.9(16.19 to 19.87) | 46(41 to 51) | 19.2(17.19 to 21.43) | 0.1(0.03 to 0.12) |
| Japan | 20254(17955 to 22776) | 13.7(12.26 to 15.44) | 46727(40955 to 53707) | 13(11.66 to 14.42) | -0.1(-0.08 to -0.02) |
| Singapore | 407(367 to 450) | 20.6(18.51 to 22.84) | 1306(1170 to 1462) | 18.7(16.77 to 20.9) | -0.1(-0.14 to -0.03) |
| Republic of Korea | 3812(3427 to 4210) | 13.5(12.2 to 14.84) | 13314(11869 to 14941) | 16.9(15.13 to 18.99) | 0.3(0.19 to 0.36) |
| Western Europe | 101558(91153 to 112716) | 18.2(16.4 to 20.13) | 168939(150376 to 190360) | 17.2(15.54 to 19.09) | -0.1(-0.08 to -0.02) |
| Andorra | 7(6 to 8) | 16.3(14.53 to 18.17) | 24(21 to 27) | 16.4(14.58 to 18.38) | 0(-0.04 to 0.04) |
| Austria | 2200(1957 to 2456) | 19.1(17.3 to 21.1) | 3395(3017 to 3791) | 18.7(16.83 to 20.68) | 0(-0.07 to 0.03) |
| Belgium | 4663(4080 to 5216) | 31.3(27.62 to 34.77) | 7192(6325 to 8235) | 28.5(25.48 to 31.97) | -0.1(-0.15 to -0.02) |
| Cyprus | 113(99 to 130) | 17.4(15.47 to 19.57) | 328(290 to 375) | 19.5(17.29 to 22.03) | 0.1(0.07 to 0.18) |
| Denmark | 930(814 to 1056) | 11.7(10.37 to 13.16) | 1470(1291 to 1659) | 13(11.6 to 14.66) | 0.1(0.07 to 0.16) |
| Finland | 1182(1042 to 1345) | 17.5(15.67 to 19.69) | 2298(2015 to 2617) | 19.4(17.32 to 21.77) | 0.1(0.07 to 0.14) |
| France | 21191(18672 to 24214) | 24.5(21.84 to 27.64) | 30537(26556 to 35381) | 19(16.79 to 21.36) | -0.2(-0.28 to -0.17) |
| Germany | 18568(16440 to 20946) | 15.3(13.73 to 17.08) | 37649(33281 to 42577) | 18.7(16.76 to 20.8) | 0.2(0.14 to 0.31) |
| Greece | 1858(1647 to 2090) | 13.4(11.98 to 14.81) | 3524(3079 to 4024) | 14.4(12.84 to 16.11) | 0.1(0.02 to 0.12) |
| Iceland | 40(35 to 45) | 13.7(12.19 to 15.43) | 99(86 to 112) | 17.5(15.47 to 19.74) | 0.3(0.22 to 0.33) |
| Ireland | 766(680 to 855) | 19.9(17.74 to 22) | 1139(1006 to 1292) | 15.7(13.92 to 17.63) | -0.2(-0.27 to -0.15) |
| Israel | 914(815 to 1022) | 20(17.87 to 22.17) | 3757(3379 to 4197) | 30.4(27.44 to 33.87) | 0.5(0.43 to 0.64) |
| Italy | 11521(10167 to 12998) | 14(12.44 to 15.68) | 18573(16396 to 20926) | 11.1(9.94 to 12.27) | -0.2(-0.23 to -0.18) |
| Luxembourg | 78(70 to 86) | 15.4(13.85 to 16.94) | 159(140 to 180) | 15.3(13.6 to 17.02) | 0(-0.06 to 0.05) |
| Malta | 147(133 to 167) | 37.9(34.24 to 42.8) | 362(323 to 406) | 37.2(33.49 to 41.44) | 0(-0.1 to 0.04) |
| Monaco | 10(9 to 11) | 13.9(12.41 to 15.58) | 15(13 to 17) | 15.3(13.66 to 17.03) | 0.1(0.06 to 0.14) |
| Netherlands | 4765(4195 to 5444) | 24.1(21.28 to 27.48) | 6947(6127 to 7975) | 20(17.74 to 22.61) | -0.2(-0.22 to -0.12) |
| Norway | 1621(1445 to 1809) | 24.4(22.05 to 26.94) | 2397(2139 to 2700) | 24.8(22.42 to 27.59) | 0(0 to 0.04) |
| San Marino | 4(4 to 5) | 13.1(11.64 to 14.74) | 9(8 to 11) | 14.3(12.76 to 16.16) | 0.1(0.06 to 0.13) |
| Portugal | 1429(1279 to 1599) | 11.5(10.34 to 12.77) | 2957(2587 to 3395) | 11.8(10.49 to 13.24) | 0(-0.05 to 0.1) |
| Spain | 9765(8716 to 10912) | 19.3(17.25 to 21.43) | 21757(19027 to 25589) | 19.6(17.46 to 22.32) | 0(-0.05 to 0.13) |
| Sweden | 2679(2366 to 3012) | 17.6(15.73 to 19.57) | 3188(2782 to 3598) | 14.9(13.23 to 16.65) | -0.2(-0.19 to -0.11) |
| Switzerland | 1313(1153 to 1491) | 12.7(11.34 to 14.31) | 2737(2418 to 3124) | 14.8(13.24 to 16.57) | 0.2(0.1 to 0.23) |
| United Kingdom | 15710(13950 to 17609) | 17.9(16.08 to 19.98) | 18279(16149 to 20627) | 14.9(13.28 to 16.61) | -0.2(-0.21 to -0.13) |
| Southern Latin America | 5289(4689 to 5911) | 13(11.53 to 14.5) | 17511(15470 to 19821) | 20.4(18.05 to 23.02) | 0.6(0.48 to 0.69) |
| Argentina | 3552(3125 to 4011) | 12.4(10.88 to 14.12) | 11741(10327 to 13301) | 21(18.52 to 23.78) | 0.7(0.56 to 0.84) |
| Chile | 941(834 to 1048) | 11(9.73 to 12.24) | 4216(3716 to 4785) | 17.9(15.78 to 20.32) | 0.6(0.5 to 0.79) |
| Uruguay | 796(701 to 894) | 20.9(18.52 to 23.48) | 1554(1370 to 1798) | 24.2(21.52 to 27.71) | 0.2(0.08 to 0.25) |
| Eastern Europe | 19041(16793 to 21502) | 7.3(6.54 to 8.22) | 24797(21959 to 27988) | 7.8(6.99 to 8.74) | 0.1(0.06 to 0.08) |
| Belarus | 916(809 to 1043) | 7.5(6.64 to 8.46) | 1139(1004 to 1290) | 7.7(6.89 to 8.67) | 0(0.01 to 0.07) |
| Estonia | 144(127 to 164) | 7.4(6.6 to 8.39) | 195(170 to 223) | 7.9(7.02 to 8.9) | 0.1(0.03 to 0.1) |
| Latvia | 316(281 to 356) | 9.4(8.4 to 10.48) | 441(390 to 497) | 11.6(10.39 to 12.86) | 0.2(0.18 to 0.3) |
| Lithuania | 251(222 to 284) | 5.7(5.09 to 6.49) | 338(297 to 385) | 6.2(5.53 to 6.98) | 0.1(0.04 to 0.11) |
| Republic of Moldova | 312(275 to 353) | 7.6(6.7 to 8.53) | 421(371 to 479) | 7.8(6.96 to 8.82) | 0(0 to 0.06) |
| Russian Federation | 12107(10706 to 13662) | 7.2(6.41 to 8.05) | 16876(14951 to 19055) | 7.8(6.99 to 8.75) | 0.1(0.08 to 0.1) |
| Ukraine | 4995(4377 to 5670) | 7.6(6.73 to 8.55) | 5387(4735 to 6088) | 7.7(6.86 to 8.62) | 0(-0.02 to 0.04) |
| Central Europe | 12546(11102 to 14108) | 9.4(8.39 to 10.45) | 24606(22180 to 27197) | 12(10.86 to 13.12) | 0.3(0.23 to 0.33) |
| Albania | 165(147 to 185) | 7.9(7.02 to 8.88) | 346(304 to 394) | 8.7(7.71 to 9.79) | 0.1(0.06 to 0.14) |
| Bosnia and Herzegovina | 283(251 to 317) | 7.9(7 to 8.85) | 472(415 to 535) | 8.9(7.93 to 9.95) | 0.1(0.08 to 0.18) |
| Bulgaria | 842(733 to 969) | 8(7.07 to 8.99) | 1176(1017 to 1351) | 8.6(7.65 to 9.72) | 0.1(0.05 to 0.12) |
| Croatia | 450(396 to 509) | 7.9(7 to 8.84) | 784(685 to 899) | 9.3(8.21 to 10.45) | 0.2(0.11 to 0.25) |
| Czechia | 1126(996 to 1265) | 8.8(7.88 to 9.85) | 2568(2266 to 2871) | 12.6(11.24 to 13.92) | 0.4(0.34 to 0.52) |
| Hungary | 1049(921 to 1191) | 7.8(6.95 to 8.78) | 1733(1535 to 1954) | 9.3(8.31 to 10.4) | 0.2(0.13 to 0.25) |
| North Macedonia | 139(124 to 157) | 8.4(7.36 to 9.48) | 240(211 to 275) | 8.8(7.79 to 9.96) | 0.1(0.02 to 0.09) |
| Montenegro | 48(43 to 55) | 8.2(7.27 to 9.3) | 78(68 to 89) | 8.8(7.83 to 9.95) | 0.1(0.04 to 0.1) |
| Poland | 5380(4767 to 6062) | 13.1(11.74 to 14.68) | 11765(10645 to 12902) | 17.1(15.65 to 18.58) | 0.3(0.22 to 0.39) |
| Romania | 1802(1587 to 2047) | 7.5(6.6 to 8.39) | 3007(2628 to 3415) | 8.4(7.44 to 9.4) | 0.1(0.08 to 0.16) |
| Serbia | 528(467 to 600) | 5.3(4.75 to 6.05) | 991(869 to 1130) | 6.8(6.1 to 7.67) | 0.3(0.22 to 0.34) |
| Slovakia | 542(476 to 612) | 9.6(8.48 to 10.76) | 977(868 to 1089) | 11.5(10.3 to 12.75) | 0.2(0.14 to 0.26) |
| Slovenia | 192(169 to 218) | 8.4(7.45 to 9.37) | 470(413 to 529) | 10.5(9.39 to 11.78) | 0.3(0.19 to 0.32) |
| Central Asia | 684(596 to 781) | 1.5(1.27 to 1.66) | 1055(918 to 1213) | 1.6(1.39 to 1.8) | 0.1(0.07 to 0.11) |
| Armenia | 38(33 to 44) | 1.5(1.3 to 1.71) | 62(54 to 71) | 1.6(1.38 to 1.81) | 0.1(0.03 to 0.1) |
| Azerbaijan | 73(64 to 84) | 1.5(1.27 to 1.66) | 128(110 to 148) | 1.6(1.39 to 1.81) | 0.1(0.05 to 0.12) |
| Georgia | 82(71 to 95) | 1.5(1.28 to 1.67) | 99(88 to 111) | 1.7(1.49 to 1.86) | 0.1(0.08 to 0.21) |
| Kazakhstan | 174(152 to 199) | 1.4(1.24 to 1.61) | 243(211 to 278) | 1.6(1.37 to 1.78) | 0.1(0.07 to 0.14) |
| Kyrgyzstan | 46(40 to 52) | 1.4(1.26 to 1.65) | 69(60 to 79) | 1.6(1.35 to 1.77) | 0.1(0.04 to 0.1) |
| Mongolia | 17(14 to 19) | 1.5(1.28 to 1.68) | 33(29 to 38) | 1.5(1.35 to 1.77) | 0.1(0.02 to 0.1) |
| Tajikistan | 47(41 to 54) | 1.5(1.32 to 1.73) | 76(66 to 89) | 1.6(1.4 to 1.83) | 0.1(0.02 to 0.09) |
| Turkmenistan | 29(25 to 34) | 1.5(1.27 to 1.67) | 57(50 to 66) | 1.6(1.37 to 1.8) | 0.1(0.04 to 0.11) |
| Uzbekistan | 179(155 to 205) | 1.5(1.29 to 1.68) | 288(246 to 338) | 1.6(1.39 to 1.81) | 0.1(0.04 to 0.11) |
| Central Latin America | 21574(19626 to 23683) | 26.9(24.14 to 29.73) | 61804(55697 to 68479) | 27.4(24.63 to 30.44) | 0(0 to 0.04) |
| Colombia | 4940(4483 to 5426) | 30.5(27.4 to 33.68) | 15262(13657 to 17106) | 28.1(25.12 to 31.25) | -0.1(-0.12 to -0.03) |
| Costa Rica | 531(479 to 585) | 30.8(27.63 to 34.19) | 1510(1347 to 1681) | 29.8(26.57 to 33.24) | 0(-0.07 to 0.01) |
| El Salvador | 605(543 to 665) | 19.4(17.21 to 21.59) | 1547(1388 to 1708) | 24.4(21.86 to 26.98) | 0.3(0.19 to 0.33) |
| Guatemala | 678(608 to 748) | 19.1(16.94 to 21.22) | 2362(2116 to 2622) | 21.6(19.33 to 24.05) | 0.1(0.07 to 0.2) |
| Honduras | 444(395 to 495) | 20.2(17.78 to 22.76) | 1151(1032 to 1282) | 19.2(17 to 21.4) | -0.1(-0.1 to -0.01) |
| Mexico | 11552(10461 to 12703) | 28.3(25.49 to 31.56) | 31500(28443 to 34792) | 29.3(26.35 to 32.62) | 0(0.01 to 0.06) |
| Nicaragua | 350(314 to 386) | 21.3(18.94 to 23.96) | 886(799 to 980) | 21.5(19.34 to 23.9) | 0(-0.05 to 0.07) |
| Panama | 452(408 to 499) | 31(27.86 to 34.45) | 1467(1316 to 1631) | 34.7(31.2 to 38.75) | 0.1(0.07 to 0.18) |
| Venezuela (Bolivarian Republic of) | 2022(1810 to 2246) | 20.3(18.05 to 22.79) | 6118(5392 to 6916) | 22.5(19.73 to 25.57) | 0.1(0.05 to 0.17) |
| Andean Latin America | 1528(1375 to 1694) | 7.2(6.43 to 8.01) | 4817(4323 to 5338) | 8.7(7.79 to 9.63) | 0.2(0.18 to 0.25) |
| Bolivia (Plurinational State of) | 221(197 to 248) | 6.6(5.87 to 7.39) | 639(569 to 718) | 7.5(6.68 to 8.37) | 0.1(0.09 to 0.17) |
| Ecuador | 390(347 to 436) | 6.9(6.14 to 7.77) | 1494(1349 to 1657) | 10.4(9.39 to 11.54) | 0.5(0.41 to 0.6) |
| Peru | 917(825 to 1016) | 7.4(6.63 to 8.22) | 2683(2395 to 2991) | 8.2(7.35 to 9.21) | 0.1(0.07 to 0.17) |
| Caribbean | 4740(4288 to 5233) | 19.3(17.43 to 21.31) | 11192(10142 to 12348) | 21.7(19.63 to 23.88) | 0.1(0.09 to 0.16) |
| Antigua and Barbuda | 13(11 to 14) | 22.1(19.94 to 24.56) | 31(28 to 35) | 35.4(31.48 to 40.24) | 0.6(0.5 to 0.75) |
| Bahamas | 48(44 to 53) | 35.3(31.83 to 39.25) | 138(124 to 154) | 40.6(36.53 to 45.84) | 0.1(0.09 to 0.21) |
| Barbados | 162(145 to 181) | 53.2(48.4 to 58.46) | 265(240 to 296) | 55.2(50.09 to 61.44) | 0(-0.02 to 0.09) |
| Belize | 20(18 to 22) | 20.5(18.63 to 22.66) | 57(51 to 63) | 22.7(20.31 to 25.18) | 0.1(0.05 to 0.15) |
| Bermuda | 10(9 to 11) | 17.9(16.08 to 19.95) | 36(31 to 41) | 26.1(23.18 to 29.81) | 0.5(0.36 to 0.61) |
| Cuba | 1483(1315 to 1668) | 14.7(13.1 to 16.46) | 3653(3263 to 4056) | 18.8(16.97 to 20.85) | 0.3(0.21 to 0.36) |
| Dominica | 25(22 to 28) | 34.2(30.58 to 38.27) | 35(31 to 39) | 38.8(34.63 to 43.52) | 0.1(0.09 to 0.18) |
| Dominican Republic | 498(446 to 552) | 13.2(11.79 to 14.73) | 1358(1221 to 1513) | 15.1(13.6 to 16.93) | 0.1(0.11 to 0.19) |
| Grenada | 18(16 to 20) | 22(19.89 to 24.32) | 36(33 to 41) | 38.9(34.82 to 43.58) | 0.8(0.66 to 0.92) |
| Guyana | 50(45 to 55) | 13.3(12.01 to 14.65) | 97(87 to 107) | 18.6(16.71 to 20.75) | 0.4(0.33 to 0.47) |
| Haiti | 402(362 to 443) | 13.8(12.43 to 15.39) | 1096(993 to 1205) | 18.2(16.44 to 20.16) | 0.3(0.26 to 0.39) |
| Jamaica | 595(534 to 662) | 32.2(28.91 to 35.69) | 1230(1101 to 1377) | 37.7(33.69 to 42.03) | 0.2(0.11 to 0.24) |
| Puerto Rico | 1027(914 to 1152) | 29.5(26.43 to 33.04) | 2074(1830 to 2370) | 25.6(22.83 to 28.89) | -0.1(-0.19 to -0.07) |
| Saint Kitts and Nevis | 15(13 to 17) | 43.4(39 to 48.53) | 20(18 to 22) | 39.7(35.53 to 44.7) | -0.1(-0.13 to -0.03) |
| Saint Lucia | 18(17 to 20) | 22.5(20.33 to 24.87) | 66(59 to 74) | 33.2(29.66 to 37.02) | 0.5(0.39 to 0.58) |
| Saint Vincent and the Grenadines | 18(17 to 20) | 26.8(24.19 to 29.39) | 43(39 to 48) | 35.5(31.91 to 39.43) | 0.3(0.26 to 0.4) |
| Suriname | 52(47 to 57) | 20.8(18.72 to 23.06) | 180(162 to 201) | 32.8(29.32 to 36.8) | 0.6(0.5 to 0.66) |
| Trinidad and Tobago | 110(99 to 123) | 13.6(12.24 to 15.2) | 342(308 to 380) | 20(17.99 to 22.24) | 0.5(0.38 to 0.56) |
| United States Virgin Islands | 19(17 to 21) | 26.7(23.97 to 29.51) | 57(50 to 64) | 34.3(30.58 to 38.51) | 0.3(0.22 to 0.36) |
| Tropical Latin America | 18638(16817 to 20505) | 20.9(18.96 to 23.13) | 56254(51230 to 62092) | 24.3(22.15 to 26.82) | 0.2(0.13 to 0.19) |
| Brazil | 18328(16535 to 20168) | 21.1(19.18 to 23.39) | 55347(50399 to 61132) | 24.5(22.32 to 27.03) | 0.2(0.13 to 0.19) |
| Paraguay | 310(280 to 344) | 13.2(11.85 to 14.71) | 907(817 to 999) | 16.6(14.93 to 18.38) | 0.3(0.2 to 0.33) |
| East Asia | 30787(26943 to 35374) | 4.5(3.91 to 5.08) | 92666(82463 to 104013) | 5.7(5.05 to 6.39) | 0.3(0.2 to 0.34) |
| China | 28227(24595 to 32537) | 4.2(3.61 to 4.75) | 83636(74445 to 94117) | 5.3(4.68 to 5.94) | 0.3(0.19 to 0.34) |
| Democratic People's Republic of Korea | 622(554 to 694) | 5.4(4.79 to 6.14) | 1806(1593 to 2029) | 6.9(6.06 to 7.74) | 0.3(0.21 to 0.31) |
| Taiwan (Province of China) | 1937(1737 to 2174) | 16.9(15.11 to 18.9) | 7225(6444 to 8013) | 18(16.05 to 19.92) | 0.1(-0.01 to 0.14) |
| Southeast Asia | 8318(7414 to 9301) | 4.1(3.63 to 4.61) | 32237(29199 to 35988) | 6.7(6.06 to 7.55) | 0.6(0.6 to 0.71) |
| Cambodia | 114(101 to 129) | 3.2(2.87 to 3.64) | 458(406 to 513) | 5.2(4.56 to 5.86) | 0.6(0.51 to 0.68) |
| Indonesia | 2392(2108 to 2729) | 3(2.61 to 3.41) | 6181(5429 to 7019) | 3.7(3.21 to 4.19) | 0.2(0.21 to 0.26) |
| Lao People's Democratic Republic | 51(45 to 57) | 3.2(2.86 to 3.6) | 166(149 to 185) | 5.2(4.61 to 5.82) | 0.6(0.54 to 0.69) |
| Malaysia | 490(437 to 549) | 6.6(5.87 to 7.48) | 2978(2680 to 3328) | 13.9(12.53 to 15.65) | 1.1(0.97 to 1.24) |
| Maldives | 3(3 to 4) | 5.6(4.98 to 6.34) | 21(19 to 23) | 8.7(7.78 to 9.76) | 0.5(0.47 to 0.64) |
| Mauritius | 34(30 to 38) | 6(5.34 to 6.88) | 137(123 to 153) | 9(8.06 to 10.14) | 0.5(0.4 to 0.6) |
| Myanmar | 677(603 to 761) | 3.9(3.46 to 4.37) | 2198(1950 to 2470) | 6.2(5.45 to 7) | 0.6(0.51 to 0.67) |
| Philippines | 1789(1602 to 1999) | 8.1(7.22 to 9.09) | 6951(6274 to 7721) | 11.6(10.37 to 12.99) | 0.4(0.42 to 0.45) |
| Sri Lanka | 274(240 to 311) | 3.1(2.74 to 3.55) | 970(849 to 1105) | 4.7(4.09 to 5.34) | 0.5(0.41 to 0.58) |
| Seychelles | 2(2 to 3) | 4.4(3.91 to 5.01) | 6(5 to 7) | 6.5(5.71 to 7.39) | 0.5(0.39 to 0.54) |
| Thailand | 1333(1187 to 1493) | 4.9(4.34 to 5.57) | 8637(7776 to 9655) | 8.9(8.03 to 10) | 0.8(0.69 to 0.97) |
| Timor-Leste | 7(6 to 8) | 3.2(2.85 to 3.62) | 31(27 to 35) | 5(4.48 to 5.67) | 0.6(0.5 to 0.65) |
| Viet Nam | 1139(1000 to 1297) | 3.1(2.75 to 3.58) | 3462(3034 to 3906) | 4.6(4.03 to 5.25) | 0.5(0.39 to 0.55) |
| Oceania | 80(70 to 90) | 4.1(3.64 to 4.61) | 242(217 to 271) | 5.3(4.74 to 5.97) | 0.3(0.26 to 0.33) |
| American Samoa | 2(2 to 2) | 13.1(11.72 to 14.68) | 7(6 to 8) | 18.6(16.63 to 20.8) | 0.4(0.33 to 0.49) |
| Cook Islands | 1(0 to 1) | 5.5(4.82 to 6.33) | 2(1 to 2) | 6.8(5.97 to 7.89) | 0.2(0.17 to 0.32) |
| Micronesia (Federated States of) | 1(1 to 2) | 4.4(3.94 to 4.99) | 3(2 to 3) | 6.5(5.77 to 7.49) | 0.5(0.39 to 0.55) |
| Fiji | 12(10 to 13) | 5.2(4.61 to 5.85) | 32(28 to 36) | 7.2(6.34 to 8.12) | 0.4(0.31 to 0.45) |
| Guam | 2(2 to 3) | 5.2(4.52 to 5.94) | 12(11 to 14) | 6.7(5.85 to 7.68) | 0.3(0.22 to 0.38) |
| Kiribati | 1(1 to 1) | 5.2(4.55 to 5.83) | 3(2 to 3) | 6.4(5.62 to 7.19) | 0.2(0.18 to 0.29) |
| Nauru | 0(0 to 0) | 5(4.42 to 5.71) | 0(0 to 0) | 5.9(5.25 to 6.73) | 0.2(0.12 to 0.24) |
| Niue | 0(0 to 0) | 6.1(5.35 to 7.07) | 0(0 to 0) | 7.3(6.36 to 8.59) | 0.2(0.13 to 0.27) |
| Marshall Islands | 1(0 to 1) | 4.8(4.26 to 5.4) | 1(1 to 1) | 6(5.29 to 6.72) | 0.3(0.18 to 0.32) |
| Northern Mariana Islands | 1(1 to 1) | 7.3(6.34 to 8.37) | 2(2 to 2) | 7.9(6.84 to 8.94) | 0.1(0 to 0.15) |
| Palau | 0(0 to 0) | 3.8(3.28 to 4.41) | 1(1 to 1) | 4.4(3.78 to 5.1) | 0.2(0.11 to 0.21) |
| Papua New Guinea | 43(38 to 49) | 3.4(3.02 to 3.86) | 140(124 to 158) | 4.4(3.9 to 4.97) | 0.3(0.23 to 0.35) |
| Samoa | 3(3 to 4) | 5.3(4.6 to 5.98) | 8(7 to 9) | 6.8(6.02 to 7.76) | 0.3(0.22 to 0.38) |
| Solomon Islands | 3(3 to 4) | 3.5(3.12 to 3.91) | 10(9 to 11) | 4.8(4.3 to 5.46) | 0.4(0.33 to 0.46) |
| Tokelau | 0(0 to 0) | 4.7(4.15 to 5.31) | 0(0 to 0) | 5.9(5.19 to 6.73) | 0.3(0.19 to 0.33) |
| Tonga | 2(2 to 2) | 4.8(4.27 to 5.46) | 5(4 to 6) | 6.8(5.94 to 7.78) | 0.4(0.33 to 0.49) |
| Tuvalu | 0(0 to 0) | 5.1(4.51 to 5.79) | 0(0 to 1) | 6(5.28 to 6.86) | 0.2(0.11 to 0.24) |
| Vanuatu | 2(2 to 2) | 3.5(3.12 to 3.99) | 6(5 to 7) | 4.7(4.16 to 5.37) | 0.3(0.27 to 0.4) |
| North Africa and Middle East | 5156(4564 to 5843) | 3.3(2.89 to 3.69) | 15697(14099 to 17652) | 4.2(3.78 to 4.77) | 0.3(0.26 to 0.34) |
| Afghanistan | 176(154 to 200) | 2.8(2.43 to 3.16) | 392(341 to 458) | 3(2.65 to 3.4) | 0.1(0.05 to 0.12) |
| Algeria | 334(291 to 384) | 3.1(2.77 to 3.56) | 1049(930 to 1187) | 3.9(3.47 to 4.43) | 0.2(0.19 to 0.31) |
| Bahrain | 6(5 to 7) | 3.1(2.73 to 3.46) | 29(25 to 33) | 4.5(3.94 to 5.17) | 0.5(0.36 to 0.6) |
| Egypt | 779(681 to 888) | 2.9(2.56 to 3.34) | 1809(1593 to 2059) | 3.5(3.03 to 3.92) | 0.2(0.14 to 0.22) |
| Iran (Islamic Republic of) | 743(648 to 851) | 3(2.68 to 3.43) | 2614(2337 to 2928) | 4(3.56 to 4.53) | 0.3(0.28 to 0.37) |
| Iraq | 236(207 to 270) | 3(2.61 to 3.35) | 714(632 to 810) | 3.5(3.04 to 3.93) | 0.2(0.13 to 0.21) |
| Jordan | 51(45 to 58) | 4.5(3.96 to 5) | 272(244 to 305) | 5.6(4.95 to 6.17) | 0.2(0.2 to 0.3) |
| Kuwait | 21(18 to 25) | 3.2(2.86 to 3.64) | 89(79 to 101) | 3.7(3.26 to 4.25) | 0.2(0.11 to 0.2) |
| Lebanon | 61(54 to 69) | 3.1(2.75 to 3.55) | 198(175 to 226) | 4(3.51 to 4.53) | 0.3(0.21 to 0.35) |
| Libya | 60(53 to 68) | 3.1(2.73 to 3.51) | 168(150 to 190) | 3.6(3.2 to 4.1) | 0.2(0.13 to 0.21) |
| Morocco | 370(325 to 420) | 2.9(2.53 to 3.26) | 894(794 to 1011) | 3.4(2.99 to 3.83) | 0.2(0.14 to 0.23) |
| Palestine | 26(23 to 30) | 2.8(2.5 to 3.22) | 74(65 to 84) | 3.4(2.98 to 3.81) | 0.2(0.15 to 0.23) |
| Oman | 21(18 to 25) | 3.2(2.84 to 3.65) | 68(58 to 78) | 4.6(4.05 to 5.25) | 0.4(0.35 to 0.55) |
| Qatar | 5(4 to 5) | 3.3(2.95 to 3.75) | 39(32 to 46) | 4.9(4.23 to 5.66) | 0.5(0.38 to 0.6) |
| Saudi Arabia | 354(317 to 398) | 7.2(6.4 to 8.17) | 1645(1483 to 1820) | 14.4(12.88 to 16.17) | 1(0.88 to 1.12) |
| Sudan | 261(227 to 300) | 2.8(2.49 to 3.23) | 601(529 to 686) | 3.3(2.88 to 3.71) | 0.2(0.11 to 0.19) |
| Syrian Arab Republic | 163(143 to 185) | 3.1(2.67 to 3.49) | 342(302 to 390) | 3.5(3.05 to 3.97) | 0.1(0.11 to 0.18) |
| Tunisia | 137(120 to 157) | 3.1(2.71 to 3.51) | 421(372 to 478) | 3.9(3.37 to 4.39) | 0.3(0.2 to 0.31) |
| Turkey | 1196(1060 to 1348) | 3.5(3.1 to 3.99) | 3728(3301 to 4202) | 4.6(4.04 to 5.2) | 0.3(0.22 to 0.41) |
| United Arab Emirates | 18(14 to 21) | 3.4(2.98 to 3.84) | 130(106 to 156) | 4.4(3.85 to 4.91) | 0.3(0.22 to 0.36) |
| Yemen | 137(118 to 160) | 2.8(2.45 to 3.21) | 407(357 to 469) | 3.1(2.76 to 3.55) | 0.1(0.07 to 0.16) |
| South Asia | 7704(6586 to 8995) | 1.4(1.22 to 1.58) | 22764(20060 to 25884) | 1.8(1.62 to 2.07) | 0.3(0.29 to 0.36) |
| Bangladesh | 672(572 to 787) | 1.4(1.2 to 1.55) | 2147(1894 to 2446) | 1.9(1.7 to 2.18) | 0.4(0.33 to 0.49) |
| Bhutan | 3(3 to 4) | 1.2(1.09 to 1.41) | 8(7 to 10) | 1.6(1.43 to 1.85) | 0.3(0.25 to 0.37) |
| India | 6101(5223 to 7140) | 1.4(1.25 to 1.6) | 18592(16390 to 21108) | 1.8(1.63 to 2.09) | 0.3(0.28 to 0.34) |
| Nepal | 119(101 to 139) | 1.3(1.11 to 1.46) | 337(294 to 385) | 1.7(1.48 to 1.9) | 0.3(0.26 to 0.38) |
| Pakistan | 808(693 to 943) | 1.3(1.12 to 1.48) | 1680(1431 to 1975) | 1.5(1.31 to 1.72) | 0.2(0.13 to 0.19) |
| Southern Sub-Saharan Africa | 3447(3133 to 3786) | 12.4(11.27 to 13.77) | 6755(6167 to 7423) | 13.2(11.93 to 14.58) | 0.1(0.05 to 0.08) |
| Botswana | 62(56 to 68) | 10.6(9.54 to 11.63) | 157(141 to 174) | 13.1(11.72 to 14.7) | 0.2(0.19 to 0.3) |
| Lesotho | 93(84 to 103) | 9.2(8.36 to 10.23) | 124(112 to 138) | 10.4(9.41 to 11.46) | 0.1(0.09 to 0.17) |
| Namibia | 77(70 to 86) | 10.7(9.68 to 11.82) | 167(151 to 184) | 12.1(10.84 to 13.44) | 0.1(0.09 to 0.17) |
| South Africa | 2719(2476 to 2980) | 13(11.75 to 14.35) | 5489(4990 to 6045) | 13.6(12.31 to 15.07) | 0.1(0.03 to 0.07) |
| Eswatini | 33(29 to 37) | 9.9(8.94 to 10.96) | 63(56 to 70) | 11.2(10.06 to 12.44) | 0.1(0.09 to 0.17) |
| Zimbabwe | 463(416 to 515) | 10.6(9.61 to 11.72) | 755(679 to 835) | 10.7(9.62 to 11.81) | 0(-0.03 to 0.04) |
| Western Sub-Saharan Africa | 4592(4043 to 5173) | 4.5(4.04 to 4.96) | 11622(10285 to 13029) | 5.4(4.9 to 6.03) | 0.2(0.19 to 0.25) |
| Benin | 107(95 to 121) | 4.2(3.81 to 4.69) | 294(259 to 331) | 4.9(4.44 to 5.46) | 0.2(0.13 to 0.21) |
| Burkina Faso | 211(185 to 239) | 4.3(3.83 to 4.7) | 532(470 to 600) | 5(4.53 to 5.57) | 0.2(0.14 to 0.23) |
| Cameroon | 232(204 to 262) | 4.4(3.95 to 4.88) | 723(640 to 819) | 5.4(4.82 to 5.96) | 0.2(0.17 to 0.27) |
| Cabo Verde | 11(10 to 12) | 4.3(3.84 to 4.74) | 29(26 to 32) | 6.5(5.82 to 7.24) | 0.5(0.43 to 0.61) |
| Chad | 138(122 to 155) | 4.1(3.66 to 4.51) | 347(305 to 392) | 4.9(4.39 to 5.35) | 0.2(0.16 to 0.25) |
| Cote d'Ivoire | 240(209 to 274) | 4.4(4.01 to 4.95) | 638(562 to 715) | 5.3(4.73 to 5.84) | 0.2(0.14 to 0.23) |
| Gambia | 20(18 to 23) | 4.3(3.85 to 4.76) | 58(51 to 65) | 5.1(4.55 to 5.61) | 0.2(0.14 to 0.23) |
| Ghana | 374(331 to 424) | 5.6(5.03 to 6.28) | 1066(957 to 1190) | 6.9(6.22 to 7.76) | 0.2(0.18 to 0.3) |
| Guinea | 156(138 to 176) | 4.2(3.8 to 4.67) | 322(285 to 361) | 5(4.5 to 5.53) | 0.2(0.14 to 0.22) |
| Guinea-Bissau | 21(18 to 24) | 4.1(3.66 to 4.52) | 42(37 to 47) | 4.6(4.19 to 5.14) | 0.1(0.11 to 0.18) |
| Liberia | 52(46 to 59) | 4.4(3.98 to 4.87) | 121(107 to 137) | 5(4.47 to 5.52) | 0.1(0.09 to 0.17) |
| Mali | 196(172 to 222) | 4.1(3.72 to 4.59) | 506(448 to 569) | 5(4.47 to 5.5) | 0.2(0.16 to 0.25) |
| Mauritania | 50(44 to 56) | 4.3(3.87 to 4.71) | 119(106 to 133) | 5.3(4.77 to 5.87) | 0.2(0.19 to 0.29) |
| Niger | 155(135 to 177) | 4.1(3.73 to 4.6) | 467(407 to 530) | 4.8(4.3 to 5.31) | 0.2(0.12 to 0.2) |
| Nigeria | 2290(2021 to 2572) | 4.5(4.06 to 5.01) | 5524(4882 to 6205) | 5.5(4.96 to 6.13) | 0.2(0.19 to 0.26) |
| Sao Tome and Principe | 3(3 to 3) | 4.2(3.81 to 4.72) | 6(5 to 7) | 5.2(4.68 to 5.79) | 0.2(0.18 to 0.27) |
| Senegal | 171(150 to 192) | 4.4(3.93 to 4.84) | 423(375 to 473) | 5.2(4.64 to 5.71) | 0.2(0.13 to 0.22) |
| Sierra Leone | 93(82 to 105) | 4.2(3.81 to 4.68) | 208(184 to 234) | 4.9(4.42 to 5.42) | 0.2(0.12 to 0.2) |
| Togo | 73(63 to 83) | 4.3(3.9 to 4.8) | 197(173 to 221) | 4.9(4.42 to 5.44) | 0.1(0.1 to 0.18) |
| Eastern Sub-Saharan Africa | 3965(3452 to 4514) | 3.9(3.44 to 4.37) | 9107(7930 to 10376) | 4(3.58 to 4.54) | 0(0.03 to 0.05) |
| Burundi | 118(103 to 134) | 3.8(3.32 to 4.26) | 251(218 to 285) | 4(3.55 to 4.54) | 0.1(0.04 to 0.09) |
| Comoros | 11(9 to 12) | 3.8(3.36 to 4.32) | 22(19 to 25) | 4(3.51 to 4.51) | 0(0.01 to 0.07) |
| Djibouti | 9(8 to 10) | 3.8(3.4 to 4.35) | 30(26 to 35) | 4(3.57 to 4.59) | 0.1(0.02 to 0.08) |
| Eritrea | 55(48 to 64) | 3.7(3.27 to 4.17) | 144(125 to 165) | 3.9(3.43 to 4.38) | 0(0.02 to 0.07) |
| Ethiopia | 1075(933 to 1227) | 3.9(3.46 to 4.41) | 2447(2136 to 2795) | 4.1(3.63 to 4.64) | 0(0.03 to 0.06) |
| Kenya | 486(424 to 557) | 4(3.58 to 4.55) | 1228(1071 to 1399) | 4.2(3.71 to 4.72) | 0(0.03 to 0.04) |
| Madagascar | 259(227 to 295) | 3.9(3.4 to 4.35) | 583(504 to 663) | 4(3.55 to 4.53) | 0(0.01 to 0.07) |
| Malawi | 198(172 to 226) | 3.8(3.37 to 4.33) | 402(350 to 459) | 4(3.52 to 4.52) | 0(0.02 to 0.07) |
| Mozambique | 283(248 to 323) | 3.7(3.32 to 4.26) | 598(520 to 682) | 3.9(3.44 to 4.37) | 0(0.01 to 0.06) |
| Rwanda | 148(128 to 168) | 3.8(3.33 to 4.25) | 301(264 to 343) | 3.9(3.49 to 4.43) | 0(0.01 to 0.07) |
| Somalia | 139(120 to 159) | 3.8(3.37 to 4.31) | 392(337 to 451) | 3.9(3.47 to 4.42) | 0(0 to 0.06) |
| South Sudan | 128(111 to 146) | 3.9(3.44 to 4.41) | 204(178 to 232) | 4(3.56 to 4.52) | 0(0 to 0.06) |
| United Republic of Tanzania | 550(479 to 624) | 3.8(3.38 to 4.32) | 1289(1123 to 1467) | 4(3.53 to 4.5) | 0(0.02 to 0.07) |
| Uganda | 347(303 to 396) | 3.8(3.39 to 4.33) | 825(715 to 943) | 4(3.5 to 4.48) | 0(0.01 to 0.06) |
| Zambia | 156(136 to 179) | 3.8(3.37 to 4.33) | 383(333 to 438) | 4(3.51 to 4.45) | 0(0.01 to 0.06) |
| Central Sub-Saharan Africa | 1132(987 to 1279) | 4.1(3.72 to 4.58) | 2823(2471 to 3202) | 4.2(3.79 to 4.67) | 0(0 to 0.04) |
| Angola | 198(171 to 226) | 3.8(3.38 to 4.19) | 605(524 to 689) | 4.2(3.75 to 4.62) | 0.1(0.08 to 0.14) |
| Central African Republic | 54(47 to 61) | 3.6(3.27 to 4.02) | 106(92 to 121) | 3.8(3.38 to 4.19) | 0(0.02 to 0.07) |
| Congo | 51(45 to 58) | 4(3.58 to 4.42) | 130(114 to 147) | 4.4(3.95 to 4.86) | 0.1(0.07 to 0.13) |
| Democratic Republic of the Congo | 795(692 to 899) | 4.3(3.86 to 4.75) | 1898(1659 to 2155) | 4.2(3.77 to 4.66) | 0(-0.05 to 0.01) |
| Equatorial Guinea | 9(7 to 10) | 3.5(3.18 to 3.92) | 31(27 to 36) | 4.7(4.26 to 5.24) | 0.3(0.28 to 0.4) |
| Gabon | 26(23 to 29) | 4.3(3.86 to 4.75) | 52(46 to 59) | 4.7(4.25 to 5.28) | 0.1(0.07 to 0.15) |

**Table S2: Incident cases of decubitus ulcer in 1990 and 2019 for both sexes and percentage change of age-standardized rates (ASR) by location.**

| **Regions or countries** | **1990** | | **2019** | | **Percentage change in ASR from 1990 and 2019** |
| --- | --- | --- | --- | --- | --- |
|  | **Counts (95% UI)** | **Rate (95% UI)** | **Counts (95% UI)** | **Rate (95% UI)** |  |
| Global | 1541945(1389163 to 1720928) | 46.5(41.72 to 52.02) | 3170796(2875433 to 3499729) | 41.8(37.8 to 46.22) | -0.1(-0.12 to -0.08) |
| High-income North America | 513842(457573 to 576450) | 142.1(127.48 to 158.42) | 819080(752601 to 886065) | 128(118.5 to 138.24) | -0.1(-0.14 to -0.06) |
| Canada | 36416(32722 to 40747) | 116.2(104.77 to 129.5) | 74580(66179 to 84618) | 109.8(98.19 to 123.43) | -0.1(-0.1 to -0.01) |
| Greenland | 32(28 to 35) | 97.8(86.59 to 109.71) | 61(54 to 70) | 101.9(90.62 to 115.76) | 0(0.01 to 0.08) |
| United States of America | 477382(424199 to 535030) | 144.5(129.6 to 161.31) | 744426(686418 to 804927) | 130.3(120.77 to 140.39) | -0.1(-0.14 to -0.05) |
| Australasia | 11134(9892 to 12413) | 51(45.55 to 56.62) | 23895(20996 to 27058) | 46.3(41.13 to 51.74) | -0.1(-0.14 to -0.05) |
| Australia | 9325(8303 to 10401) | 51.5(45.98 to 57.08) | 20399(17825 to 23182) | 46.7(41.35 to 52.41) | -0.1(-0.14 to -0.04) |
| New Zealand | 1809(1603 to 2056) | 48.8(43.35 to 55.17) | 3497(3121 to 3913) | 44.1(39.85 to 48.92) | -0.1(-0.14 to -0.05) |
| High-income Asia Pacific | 89823(80581 to 101351) | 50.7(45.59 to 57.08) | 226243(199724 to 258461) | 49.9(45.11 to 55.4) | 0(-0.04 to 0.01) |
| Brunei Darussalam | 66(59 to 74) | 65.6(59.23 to 73.04) | 166(150 to 185) | 70.2(63.06 to 78.37) | 0.1(0.02 to 0.12) |
| Japan | 74338(66228 to 84306) | 50.4(45.06 to 56.79) | 172584(151302 to 198159) | 47.6(42.85 to 52.97) | -0.1(-0.09 to -0.02) |
| Singapore | 1473(1339 to 1623) | 74.4(66.91 to 82.53) | 4742(4257 to 5317) | 67.8(60.73 to 75.77) | -0.1(-0.14 to -0.03) |
| Republic of Korea | 13946(12609 to 15434) | 49.7(45.18 to 54.69) | 48750(43676 to 55041) | 61.8(55.62 to 69.17) | 0.2(0.18 to 0.33) |
| Western Europe | 373634(333338 to 418911) | 66.6(60.06 to 73.84) | 622022(551286 to 704091) | 63.2(57.05 to 70.32) | -0.1(-0.07 to -0.02) |
| Andorra | 26(23 to 29) | 59.8(53.32 to 66.65) | 87(77 to 99) | 60.1(53.38 to 67.53) | 0(-0.04 to 0.05) |
| Austria | 8351(7443 to 9378) | 72.2(65.52 to 79.69) | 12764(11361 to 14287) | 70(63.2 to 77.35) | 0(-0.08 to 0.02) |
| Belgium | 17393(15260 to 19628) | 116.3(103.29 to 129.76) | 26921(23676 to 30732) | 106.4(94.95 to 119.61) | -0.1(-0.15 to -0.02) |
| Cyprus | 410(359 to 472) | 62.9(55.84 to 71.34) | 1191(1053 to 1370) | 70.4(62.55 to 79.93) | 0.1(0.07 to 0.18) |
| Denmark | 3419(3010 to 3912) | 42.8(38.3 to 48.25) | 5403(4719 to 6134) | 47.7(42.49 to 53.62) | 0.1(0.07 to 0.16) |
| Finland | 4425(3918 to 5018) | 65.5(58.78 to 73.38) | 8605(7551 to 9903) | 72.2(64.53 to 81.24) | 0.1(0.07 to 0.14) |
| France | 76807(67879 to 88212) | 88.7(78.49 to 100.55) | 112056(96748 to 129922) | 69.6(62.02 to 78.88) | -0.2(-0.27 to -0.16) |
| Germany | 68997(61218 to 77476) | 56.8(51.02 to 63.24) | 138946(121998 to 156613) | 69(61.63 to 76.66) | 0.2(0.14 to 0.29) |
| Greece | 6863(6109 to 7699) | 49.2(44.41 to 54.37) | 13048(11384 to 14950) | 52.9(47.29 to 59.28) | 0.1(0.02 to 0.12) |
| Iceland | 148(131 to 168) | 51(45.5 to 57.22) | 369(325 to 419) | 65.2(58.25 to 73.59) | 0.3(0.22 to 0.34) |
| Ireland | 2792(2476 to 3141) | 72.3(64.5 to 80.61) | 4196(3707 to 4736) | 57.6(51.41 to 64.5) | -0.2(-0.25 to -0.14) |
| Israel | 3328(2970 to 3733) | 72.5(64.76 to 80.92) | 13479(12139 to 15085) | 109.3(98.76 to 121.12) | 0.5(0.41 to 0.62) |
| Italy | 42497(37126 to 48344) | 51.3(45.42 to 58.11) | 68755(60713 to 77729) | 40.8(36.57 to 45.51) | -0.2(-0.23 to -0.18) |
| Luxembourg | 285(255 to 319) | 56.1(50.42 to 62.06) | 581(513 to 656) | 55.6(49.98 to 62.15) | 0(-0.06 to 0.05) |
| Malta | 531(478 to 604) | 136.5(123.16 to 155.67) | 1303(1158 to 1467) | 134.2(120.34 to 149.93) | 0(-0.1 to 0.05) |
| Monaco | 36(32 to 42) | 51.3(45.89 to 57.25) | 56(49 to 64) | 56.2(50.42 to 63.01) | 0.1(0.06 to 0.14) |
| Netherlands | 17297(15163 to 19723) | 87.5(76.86 to 99.1) | 25396(22478 to 29388) | 72.9(65.41 to 83.33) | -0.2(-0.21 to -0.12) |
| Norway | 6042(5391 to 6765) | 90.5(81.56 to 100.51) | 8947(7948 to 10076) | 92.3(83.18 to 102.61) | 0(0 to 0.04) |
| San Marino | 15(13 to 17) | 48.4(42.98 to 54.52) | 35(31 to 40) | 52.8(47.33 to 59.29) | 0.1(0.06 to 0.13) |
| Portugal | 5173(4616 to 5827) | 41.4(37.44 to 46.32) | 10902(9546 to 12506) | 43.1(38.46 to 48.32) | 0(-0.03 to 0.12) |
| Spain | 35768(31866 to 40271) | 70.4(63.09 to 78.55) | 79555(69289 to 93456) | 71.7(63.99 to 81.45) | 0(-0.05 to 0.13) |
| Sweden | 9945(8739 to 11294) | 65(58.4 to 72.7) | 11777(10369 to 13299) | 54.9(49.19 to 61.63) | -0.2(-0.2 to -0.11) |
| Switzerland | 4824(4262 to 5532) | 46.7(41.67 to 52.72) | 10006(8844 to 11378) | 54(48.4 to 60.52) | 0.2(0.1 to 0.23) |
| United Kingdom | 57951(51211 to 65676) | 65.8(58.93 to 74.31) | 67100(59216 to 75970) | 54.4(48.71 to 61.08) | -0.2(-0.21 to -0.13) |
| Southern Latin America | 19832(17538 to 22193) | 48.7(42.94 to 54.78) | 64975(57404 to 73761) | 75.6(66.85 to 85.59) | 0.6(0.46 to 0.66) |
| Argentina | 13365(11729 to 15044) | 46.8(40.77 to 53) | 43501(38347 to 49593) | 77.9(68.77 to 88.51) | 0.7(0.53 to 0.8) |
| Chile | 3541(3145 to 3938) | 41.4(36.87 to 46.13) | 15750(13748 to 17908) | 66.8(58.34 to 75.8) | 0.6(0.49 to 0.76) |
| Uruguay | 2926(2580 to 3304) | 76.9(67.94 to 86.7) | 5721(5059 to 6600) | 89.3(79.56 to 102.2) | 0.2(0.08 to 0.25) |
| Eastern Europe | 69658(61760 to 78840) | 26.7(23.95 to 30.05) | 90580(80446 to 102015) | 28.5(25.55 to 31.91) | 0.1(0.05 to 0.08) |
| Belarus | 3363(2987 to 3829) | 27.4(24.4 to 30.97) | 4177(3697 to 4724) | 28.3(25.35 to 31.82) | 0(0.01 to 0.06) |
| Estonia | 529(467 to 601) | 27.2(24.27 to 30.63) | 715(627 to 814) | 28.9(25.77 to 32.46) | 0.1(0.03 to 0.1) |
| Latvia | 1178(1048 to 1331) | 34.9(31.24 to 39.01) | 1633(1445 to 1845) | 42.8(38.44 to 47.56) | 0.2(0.18 to 0.29) |
| Lithuania | 910(806 to 1030) | 20.8(18.63 to 23.38) | 1221(1077 to 1390) | 22.3(20.04 to 25.05) | 0.1(0.04 to 0.11) |
| Republic of Moldova | 1146(1013 to 1291) | 27.7(24.68 to 31.15) | 1547(1368 to 1758) | 28.7(25.65 to 32.38) | 0(0 to 0.06) |
| Russian Federation | 44252(39271 to 49988) | 26.2(23.42 to 29.37) | 61598(54852 to 69366) | 28.5(25.55 to 31.94) | 0.1(0.08 to 0.1) |
| Ukraine | 18280(16161 to 20794) | 27.7(24.68 to 31.25) | 19689(17381 to 22211) | 28(25.05 to 31.36) | 0(-0.02 to 0.04) |
| Central Europe | 46588(41338 to 52520) | 34.8(31.3 to 38.92) | 91933(83069 to 101445) | 44.6(40.73 to 48.85) | 0.3(0.23 to 0.33) |
| Albania | 609(547 to 681) | 29.3(26.18 to 32.84) | 1286(1138 to 1465) | 32.2(28.74 to 36.31) | 0.1(0.06 to 0.14) |
| Bosnia and Herzegovina | 1047(936 to 1176) | 29.3(26.06 to 33.01) | 1753(1552 to 1980) | 32.9(29.46 to 37) | 0.1(0.07 to 0.18) |
| Bulgaria | 3124(2744 to 3593) | 29.6(26.4 to 33.36) | 4383(3845 to 5053) | 32(28.49 to 36.12) | 0.1(0.05 to 0.12) |
| Croatia | 1670(1471 to 1889) | 29.3(26.04 to 32.75) | 2927(2555 to 3377) | 34.4(30.55 to 38.86) | 0.2(0.11 to 0.27) |
| Czechia | 4195(3729 to 4722) | 32.7(29.4 to 36.68) | 9496(8357 to 10686) | 46.3(41.43 to 51.75) | 0.4(0.34 to 0.5) |
| Hungary | 3900(3433 to 4468) | 29(25.94 to 32.6) | 6436(5678 to 7279) | 34.3(30.77 to 38.37) | 0.2(0.13 to 0.24) |
| North Macedonia | 515(460 to 580) | 31(27.61 to 34.99) | 891(787 to 1014) | 32.6(29.06 to 36.97) | 0.1(0.02 to 0.09) |
| Montenegro | 179(159 to 203) | 30.5(27.09 to 34.49) | 291(255 to 330) | 32.7(28.97 to 36.62) | 0.1(0.04 to 0.11) |
| Poland | 20027(17800 to 22540) | 48.8(43.85 to 54.55) | 44352(40692 to 48623) | 64.1(59.39 to 69.67) | 0.3(0.23 to 0.4) |
| Romania | 6671(5887 to 7519) | 27.6(24.71 to 31) | 11155(9793 to 12655) | 31(27.65 to 34.69) | 0.1(0.08 to 0.16) |
| Serbia | 1906(1690 to 2157) | 19.3(17.14 to 21.83) | 3567(3111 to 4103) | 24.6(21.84 to 27.7) | 0.3(0.22 to 0.33) |
| Slovakia | 2029(1796 to 2314) | 35.9(31.93 to 40.48) | 3652(3270 to 4127) | 42.8(38.54 to 47.9) | 0.2(0.14 to 0.26) |
| Slovenia | 715(634 to 809) | 31.2(27.8 to 35.02) | 1744(1533 to 1981) | 39(34.75 to 43.64) | 0.3(0.19 to 0.31) |
| Central Asia | 2509(2195 to 2867) | 5.4(4.68 to 6.12) | 3862(3378 to 4406) | 5.8(5.12 to 6.59) | 0.1(0.06 to 0.1) |
| Armenia | 139(122 to 160) | 5.5(4.79 to 6.28) | 227(198 to 260) | 5.8(5.09 to 6.66) | 0.1(0.03 to 0.1) |
| Azerbaijan | 269(235 to 305) | 5.4(4.7 to 6.12) | 470(408 to 541) | 5.8(5.1 to 6.66) | 0.1(0.05 to 0.12) |
| Georgia | 302(263 to 346) | 5.4(4.68 to 6.11) | 363(323 to 407) | 6.1(5.47 to 6.82) | 0.1(0.08 to 0.21) |
| Kazakhstan | 638(558 to 730) | 5.2(4.55 to 5.93) | 889(777 to 1019) | 5.7(5.01 to 6.51) | 0.1(0.06 to 0.14) |
| Kyrgyzstan | 168(147 to 190) | 5.3(4.65 to 6.06) | 252(220 to 287) | 5.7(4.97 to 6.48) | 0.1(0.04 to 0.1) |
| Mongolia | 61(52 to 71) | 5.4(4.73 to 6.18) | 121(105 to 139) | 5.7(5 to 6.51) | 0.1(0.02 to 0.09) |
| Tajikistan | 172(150 to 196) | 5.6(4.89 to 6.38) | 280(240 to 326) | 5.9(5.14 to 6.77) | 0.1(0.02 to 0.09) |
| Turkmenistan | 106(92 to 124) | 5.4(4.69 to 6.15) | 210(184 to 239) | 5.8(5.04 to 6.6) | 0.1(0.04 to 0.11) |
| Uzbekistan | 654(571 to 749) | 5.4(4.75 to 6.18) | 1052(904 to 1229) | 5.8(5.11 to 6.65) | 0.1(0.04 to 0.11) |
| Central Latin America | 79891(72540 to 88097) | 100.2(89.74 to 111.54) | 231151(208858 to 255964) | 102.7(92.34 to 114.01) | 0(0 to 0.05) |
| Colombia | 18268(16662 to 20169) | 113.2(101.61 to 125.72) | 57068(51028 to 64099) | 105.1(94.12 to 118.02) | -0.1(-0.11 to -0.03) |
| Costa Rica | 1970(1777 to 2181) | 114.6(102.58 to 127.97) | 5624(5034 to 6280) | 111.1(99.28 to 124.31) | 0(-0.07 to 0.01) |
| El Salvador | 2263(2040 to 2503) | 73(65.03 to 81.48) | 5819(5220 to 6496) | 91.6(81.86 to 102.15) | 0.3(0.19 to 0.33) |
| Guatemala | 2526(2272 to 2785) | 72.6(64.53 to 80.21) | 8884(7945 to 9848) | 81.8(73.36 to 91) | 0.1(0.08 to 0.2) |
| Honduras | 1649(1478 to 1839) | 76(67.26 to 85.9) | 4294(3845 to 4772) | 72(64.34 to 80.56) | -0.1(-0.09 to -0.02) |
| Mexico | 42722(38597 to 47200) | 105.4(94.26 to 118.24) | 117861(106685 to 131023) | 109.8(98.96 to 122.23) | 0(0.01 to 0.07) |
| Nicaragua | 1298(1172 to 1433) | 80.2(71.41 to 89.83) | 3309(2980 to 3667) | 80.7(72.36 to 89.89) | 0(-0.05 to 0.07) |
| Panama | 1677(1519 to 1861) | 115.3(103.67 to 128.67) | 5425(4867 to 6080) | 128.5(115.46 to 143.91) | 0.1(0.06 to 0.17) |
| Venezuela (Bolivarian Republic of) | 7519(6764 to 8324) | 76.3(67.93 to 85.74) | 22868(20294 to 25916) | 84.2(74.28 to 96.28) | 0.1(0.05 to 0.17) |
| Andean Latin America | 5641(5077 to 6257) | 26.6(23.87 to 29.73) | 17942(16165 to 19885) | 32.4(29.22 to 35.93) | 0.2(0.18 to 0.26) |
| Bolivia (Plurinational State of) | 818(731 to 918) | 24.6(21.98 to 27.66) | 2371(2117 to 2662) | 27.8(24.82 to 31.1) | 0.1(0.09 to 0.17) |
| Ecuador | 1437(1287 to 1608) | 25.6(22.83 to 28.69) | 5641(5103 to 6242) | 39.4(35.66 to 43.34) | 0.5(0.45 to 0.63) |
| Peru | 3387(3053 to 3740) | 27.4(24.59 to 30.53) | 9930(8899 to 11056) | 30.5(27.31 to 34.04) | 0.1(0.07 to 0.17) |
| Caribbean | 17501(15706 to 19360) | 71.2(64.06 to 78.81) | 41506(37440 to 45961) | 80.3(72.4 to 88.88) | 0.1(0.1 to 0.16) |
| Antigua and Barbuda | 47(42 to 53) | 81.9(73.62 to 91.72) | 114(102 to 129) | 129.1(114.29 to 146.98) | 0.6(0.47 to 0.71) |
| Bahamas | 175(159 to 193) | 128.3(115.17 to 142.3) | 499(449 to 562) | 147.2(131.78 to 167.01) | 0.1(0.09 to 0.21) |
| Barbados | 582(521 to 648) | 190.7(173.39 to 209.8) | 955(855 to 1067) | 198.4(178.78 to 220.74) | 0(-0.01 to 0.09) |
| Belize | 73(66 to 80) | 76.6(69.19 to 84.98) | 211(191 to 233) | 84.3(75.79 to 94.31) | 0.1(0.05 to 0.15) |
| Bermuda | 38(34 to 42) | 66.6(59.99 to 74.44) | 131(115 to 151) | 96.1(85.09 to 109.43) | 0.4(0.35 to 0.58) |
| Cuba | 5522(4901 to 6202) | 54.9(48.91 to 61.4) | 13636(12137 to 15172) | 70.2(62.86 to 77.83) | 0.3(0.21 to 0.35) |
| Dominica | 92(82 to 104) | 125(112.09 to 140.2) | 128(113 to 145) | 141.8(126.06 to 160.04) | 0.1(0.08 to 0.18) |
| Dominican Republic | 1851(1660 to 2058) | 49.6(44.55 to 55.39) | 5081(4575 to 5679) | 56.8(51 to 63.5) | 0.1(0.11 to 0.19) |
| Grenada | 67(60 to 74) | 81.8(73.82 to 90.69) | 133(119 to 148) | 142(126.71 to 158.68) | 0.7(0.63 to 0.88) |
| Guyana | 186(168 to 205) | 50.4(45.62 to 55.95) | 364(328 to 403) | 70.1(63.25 to 78.23) | 0.4(0.33 to 0.46) |
| Haiti | 1507(1365 to 1665) | 52.6(47.37 to 58.62) | 4119(3744 to 4533) | 69.2(62.45 to 77.15) | 0.3(0.26 to 0.38) |
| Jamaica | 2170(1940 to 2413) | 117.3(105.43 to 130.22) | 4471(3975 to 5010) | 137(122.2 to 153.31) | 0.2(0.11 to 0.23) |
| Puerto Rico | 3745(3314 to 4199) | 107.5(95.45 to 120.66) | 7636(6732 to 8751) | 94.4(84.19 to 107.02) | -0.1(-0.17 to -0.07) |
| Saint Kitts and Nevis | 54(48 to 61) | 158.8(141.92 to 176.21) | 72(65 to 80) | 145.1(129.96 to 163.68) | -0.1(-0.13 to -0.04) |
| Saint Lucia | 68(61 to 75) | 83.6(75.22 to 92.74) | 244(218 to 274) | 122(108.97 to 137.21) | 0.5(0.37 to 0.55) |
| Saint Vincent and the Grenadines | 68(61 to 75) | 98.8(89.06 to 109.28) | 158(142 to 176) | 130.1(115.91 to 144.67) | 0.3(0.25 to 0.38) |
| Suriname | 193(175 to 213) | 77.5(69.61 to 85.76) | 661(595 to 736) | 120.5(107.88 to 135.02) | 0.6(0.48 to 0.63) |
| Trinidad and Tobago | 412(371 to 460) | 51.2(46.17 to 57.01) | 1281(1146 to 1427) | 74.8(66.95 to 83.62) | 0.5(0.39 to 0.54) |
| United States Virgin Islands | 69(62 to 76) | 98.1(87.8 to 109.19) | 208(183 to 235) | 125.4(111.53 to 141.64) | 0.3(0.22 to 0.35) |
| Tropical Latin America | 69228(62850 to 76635) | 77.9(70.6 to 86.18) | 210101(191663 to 230778) | 90.8(82.65 to 99.99) | 0.2(0.14 to 0.2) |
| Brazil | 68078(61818 to 75368) | 78.8(71.36 to 87.14) | 206736(188619 to 227106) | 91.5(83.31 to 100.75) | 0.2(0.13 to 0.19) |
| Paraguay | 1150(1039 to 1278) | 49.1(44.25 to 54.85) | 3365(3043 to 3714) | 61.7(55.34 to 68.25) | 0.3(0.2 to 0.31) |
| East Asia | 114703(100350 to 131356) | 16.7(14.68 to 19.14) | 350449(311845 to 394816) | 21.4(18.99 to 24.15) | 0.3(0.22 to 0.35) |
| China | 105267(91931 to 121496) | 15.6(13.62 to 17.92) | 316525(281279 to 357029) | 19.9(17.64 to 22.5) | 0.3(0.21 to 0.35) |
| Democratic People's Republic of Korea | 2342(2086 to 2618) | 20.6(18.21 to 23.3) | 6851(6044 to 7708) | 26(22.98 to 29.39) | 0.3(0.21 to 0.31) |
| Taiwan (Province of China) | 7094(6343 to 7990) | 61.8(55.18 to 69.55) | 27073(24091 to 30155) | 67.3(59.99 to 74.89) | 0.1(0.02 to 0.16) |
| Southeast Asia | 31438(28039 to 35197) | 15.6(13.83 to 17.53) | 120615(108476 to 134340) | 25.3(22.68 to 28.39) | 0.6(0.58 to 0.68) |
| Cambodia | 437(388 to 491) | 12.6(11.16 to 14.12) | 1737(1549 to 1949) | 19.7(17.43 to 22.24) | 0.6(0.49 to 0.66) |
| Indonesia | 9022(7948 to 10263) | 11.4(10.02 to 13.01) | 23312(20536 to 26455) | 13.9(12.29 to 15.85) | 0.2(0.21 to 0.25) |
| Lao People's Democratic Republic | 195(173 to 219) | 12.5(11.17 to 14.07) | 630(565 to 704) | 19.8(17.45 to 22.26) | 0.6(0.51 to 0.65) |
| Malaysia | 1822(1629 to 2044) | 24.7(21.86 to 28) | 10827(9675 to 12075) | 50.8(45.12 to 56.87) | 1.1(0.95 to 1.17) |
| Maldives | 12(11 to 14) | 21(18.66 to 23.63) | 77(69 to 87) | 32.2(28.65 to 36.43) | 0.5(0.46 to 0.61) |
| Mauritius | 126(112 to 142) | 22.6(19.93 to 25.75) | 506(450 to 566) | 33.3(29.56 to 37.47) | 0.5(0.38 to 0.57) |
| Myanmar | 2587(2320 to 2887) | 15(13.31 to 16.85) | 8286(7383 to 9293) | 23.3(20.58 to 26.45) | 0.6(0.48 to 0.63) |
| Philippines | 6821(6111 to 7601) | 31.3(27.93 to 35.14) | 26550(23958 to 29446) | 44.7(39.98 to 50.14) | 0.4(0.41 to 0.45) |
| Sri Lanka | 1031(905 to 1175) | 11.8(10.42 to 13.5) | 3654(3198 to 4180) | 17.6(15.39 to 20.13) | 0.5(0.4 to 0.56) |
| Seychelles | 9(8 to 11) | 16.7(14.77 to 19.04) | 22(19 to 25) | 24.3(21.45 to 27.81) | 0.5(0.38 to 0.52) |
| Thailand | 4982(4440 to 5607) | 18.5(16.25 to 21) | 31700(28426 to 35581) | 32.8(29.41 to 36.96) | 0.8(0.66 to 0.93) |
| Timor-Leste | 27(24 to 30) | 12.4(11.08 to 14.04) | 117(104 to 132) | 19.2(17.05 to 21.75) | 0.5(0.48 to 0.62) |
| Viet Nam | 4325(3807 to 4927) | 12(10.52 to 13.76) | 13038(11507 to 14716) | 17.4(15.3 to 19.89) | 0.5(0.38 to 0.53) |
| Oceania | 301(267 to 337) | 15.7(13.97 to 17.68) | 913(818 to 1020) | 20.2(17.91 to 22.7) | 0.3(0.25 to 0.32) |
| American Samoa | 8(7 to 8) | 48.4(42.88 to 54.31) | 26(23 to 29) | 68.4(61.3 to 76.31) | 0.4(0.33 to 0.49) |
| Cook Islands | 2(2 to 2) | 20.6(18.05 to 23.84) | 6(5 to 7) | 25.4(22.3 to 29.48) | 0.2(0.16 to 0.3) |
| Micronesia (Federated States of) | 5(5 to 6) | 17(15.13 to 19.23) | 10(9 to 11) | 24.8(21.86 to 28.29) | 0.5(0.38 to 0.53) |
| Fiji | 44(39 to 49) | 19.9(17.63 to 22.42) | 119(106 to 134) | 27.2(23.87 to 30.95) | 0.4(0.3 to 0.44) |
| Guam | 9(8 to 10) | 19.4(16.85 to 22.37) | 45(39 to 52) | 25(21.64 to 28.85) | 0.3(0.21 to 0.37) |
| Kiribati | 5(4 to 5) | 19.9(17.45 to 22.4) | 10(9 to 11) | 24.4(21.48 to 27.58) | 0.2(0.18 to 0.28) |
| Nauru | 0(0 to 0) | 18.9(16.64 to 21.52) | 0(0 to 1) | 22.4(19.68 to 25.46) | 0.2(0.13 to 0.25) |
| Niue | 1(0 to 1) | 23.1(19.88 to 26.72) | 1(0 to 1) | 27.4(23.75 to 32.04) | 0.2(0.13 to 0.27) |
| Marshall Islands | 2(2 to 2) | 18.4(16.26 to 20.86) | 4(4 to 5) | 22.6(20.11 to 25.51) | 0.2(0.17 to 0.3) |
| Northern Mariana Islands | 2(2 to 3) | 26.8(23.37 to 30.95) | 8(7 to 9) | 29.1(25.25 to 33.59) | 0.1(0.01 to 0.15) |
| Palau | 1(1 to 1) | 14.4(12.44 to 16.63) | 2(2 to 3) | 16.6(14.32 to 19.24) | 0.2(0.1 to 0.2) |
| Papua New Guinea | 165(144 to 187) | 13.3(11.69 to 14.93) | 528(470 to 595) | 16.9(14.95 to 19.09) | 0.3(0.22 to 0.34) |
| Samoa | 13(12 to 15) | 19.9(17.5 to 22.76) | 30(27 to 34) | 25.7(22.62 to 29.33) | 0.3(0.22 to 0.36) |
| Solomon Islands | 12(10 to 14) | 13.3(11.95 to 15.02) | 36(33 to 41) | 18.4(16.42 to 20.86) | 0.4(0.31 to 0.45) |
| Tokelau | 0(0 to 0) | 18(15.79 to 20.41) | 0(0 to 0) | 22.3(19.61 to 25.49) | 0.2(0.17 to 0.31) |
| Tonga | 8(7 to 9) | 18.3(16.15 to 20.81) | 19(17 to 22) | 25.5(22.36 to 29.42) | 0.4(0.32 to 0.47) |
| Tuvalu | 1(1 to 1) | 19.6(17.22 to 22.26) | 2(2 to 2) | 22.8(19.99 to 26.07) | 0.2(0.1 to 0.23) |
| Vanuatu | 7(6 to 7) | 13.6(11.99 to 15.45) | 22(20 to 25) | 18(15.99 to 20.53) | 0.3(0.26 to 0.39) |
| North Africa and Middle East | 19166(17107 to 21669) | 12.3(10.92 to 13.84) | 58384(52568 to 65387) | 15.9(14.15 to 17.85) | 0.3(0.25 to 0.34) |
| Afghanistan | 662(581 to 751) | 10.5(9.33 to 12.01) | 1459(1277 to 1685) | 11.4(10.11 to 12.95) | 0.1(0.05 to 0.12) |
| Algeria | 1237(1082 to 1425) | 11.8(10.48 to 13.26) | 3916(3494 to 4415) | 14.7(12.99 to 16.63) | 0.2(0.19 to 0.31) |
| Bahrain | 21(18 to 24) | 11.5(10.29 to 13.01) | 105(92 to 121) | 16.9(14.77 to 19.33) | 0.5(0.37 to 0.59) |
| Egypt | 2897(2554 to 3305) | 11(9.75 to 12.54) | 6726(5959 to 7622) | 13(11.4 to 14.7) | 0.2(0.14 to 0.22) |
| Iran (Islamic Republic of) | 2741(2406 to 3143) | 11.3(10.03 to 12.75) | 9763(8738 to 10984) | 15(13.41 to 16.92) | 0.3(0.28 to 0.38) |
| Iraq | 879(781 to 996) | 11.1(9.84 to 12.59) | 2651(2365 to 3005) | 13(11.49 to 14.73) | 0.2(0.13 to 0.21) |
| Jordan | 189(169 to 213) | 16.7(14.77 to 18.65) | 1009(909 to 1127) | 20.7(18.41 to 23.28) | 0.2(0.19 to 0.3) |
| Kuwait | 78(68 to 90) | 12(10.72 to 13.59) | 330(293 to 373) | 13.9(12.25 to 15.82) | 0.2(0.11 to 0.2) |
| Lebanon | 227(202 to 256) | 11.7(10.34 to 13.24) | 740(653 to 843) | 14.8(13.08 to 16.9) | 0.3(0.21 to 0.34) |
| Libya | 221(197 to 249) | 11.6(10.25 to 13.09) | 628(562 to 705) | 13.6(12 to 15.36) | 0.2(0.13 to 0.21) |
| Morocco | 1377(1219 to 1571) | 10.8(9.59 to 12.16) | 3344(2982 to 3772) | 12.8(11.31 to 14.39) | 0.2(0.14 to 0.23) |
| Palestine | 96(85 to 109) | 10.6(9.45 to 12.07) | 273(242 to 309) | 12.7(11.27 to 14.26) | 0.2(0.15 to 0.23) |
| Oman | 78(68 to 90) | 12(10.64 to 13.62) | 248(215 to 286) | 17.1(15.09 to 19.52) | 0.4(0.35 to 0.52) |
| Qatar | 17(14 to 20) | 12.3(10.93 to 13.9) | 141(118 to 168) | 18.3(15.85 to 21.34) | 0.5(0.39 to 0.61) |
| Saudi Arabia | 1289(1163 to 1448) | 26.3(23.42 to 29.89) | 5918(5348 to 6548) | 52(46.48 to 58.44) | 1(0.87 to 1.08) |
| Sudan | 972(854 to 1115) | 10.7(9.51 to 12.21) | 2238(1982 to 2541) | 12.3(10.91 to 14.02) | 0.1(0.11 to 0.19) |
| Syrian Arab Republic | 603(536 to 685) | 11.5(10.08 to 13.04) | 1275(1126 to 1446) | 13.1(11.56 to 14.92) | 0.1(0.11 to 0.18) |
| Tunisia | 508(447 to 581) | 11.5(10.19 to 13.12) | 1573(1382 to 1787) | 14.4(12.59 to 16.44) | 0.3(0.2 to 0.31) |
| Turkey | 4487(4007 to 5047) | 13.3(11.8 to 15.06) | 13997(12461 to 15852) | 17.3(15.31 to 19.67) | 0.3(0.22 to 0.39) |
| United Arab Emirates | 64(53 to 76) | 12.7(11.25 to 14.31) | 472(389 to 568) | 16.4(14.48 to 18.63) | 0.3(0.23 to 0.36) |
| Yemen | 511(444 to 592) | 10.6(9.31 to 12.08) | 1516(1339 to 1736) | 11.8(10.5 to 13.37) | 0.1(0.08 to 0.16) |
| South Asia | 28569(24645 to 33204) | 5.2(4.63 to 5.89) | 85350(75361 to 97351) | 6.9(6.16 to 7.82) | 0.3(0.29 to 0.36) |
| Bangladesh | 2504(2157 to 2897) | 5.2(4.59 to 5.85) | 8052(7142 to 9114) | 7.2(6.39 to 8.2) | 0.4(0.33 to 0.48) |
| Bhutan | 12(10 to 15) | 4.7(4.13 to 5.36) | 32(28 to 36) | 6.1(5.39 to 6.91) | 0.3(0.24 to 0.35) |
| India | 22617(19414 to 26449) | 5.3(4.72 to 5.99) | 69788(61670 to 79425) | 7(6.22 to 7.89) | 0.3(0.28 to 0.34) |
| Nepal | 442(376 to 517) | 4.8(4.23 to 5.53) | 1264(1103 to 1441) | 6.3(5.57 to 7.17) | 0.3(0.25 to 0.37) |
| Pakistan | 2993(2589 to 3465) | 4.8(4.23 to 5.49) | 6214(5367 to 7258) | 5.6(4.92 to 6.41) | 0.2(0.13 to 0.19) |
| Southern Sub-Saharan Africa | 12893(11783 to 14181) | 47(42.54 to 52.09) | 25327(23154 to 27929) | 49.9(44.78 to 55.34) | 0.1(0.04 to 0.08) |
| Botswana | 230(208 to 256) | 40(36.19 to 44.37) | 585(528 to 650) | 49.3(44.15 to 55.44) | 0.2(0.18 to 0.29) |
| Lesotho | 349(317 to 390) | 35.3(32.08 to 39.2) | 465(422 to 519) | 39.5(35.69 to 43.86) | 0.1(0.08 to 0.16) |
| Namibia | 290(264 to 322) | 40.5(36.78 to 45.06) | 623(566 to 687) | 45.4(40.92 to 50.72) | 0.1(0.08 to 0.16) |
| South Africa | 10175(9313 to 11179) | 48.9(44.26 to 54.24) | 20598(18770 to 22774) | 51.4(46.2 to 57.22) | 0.1(0.03 to 0.07) |
| Eswatini | 122(109 to 136) | 37.7(34.06 to 41.73) | 234(212 to 259) | 42.4(38.32 to 47.09) | 0.1(0.08 to 0.17) |
| Zimbabwe | 1726(1557 to 1923) | 40.2(36.36 to 44.38) | 2822(2554 to 3124) | 40.6(36.64 to 44.97) | 0(-0.03 to 0.05) |
| Western Sub-Saharan Africa | 16881(14972 to 18929) | 16.7(15.09 to 18.45) | 42793(38134 to 47615) | 20.4(18.42 to 22.58) | 0.2(0.19 to 0.25) |
| Benin | 396(349 to 445) | 15.9(14.29 to 17.54) | 1082(958 to 1216) | 18.5(16.72 to 20.56) | 0.2(0.13 to 0.21) |
| Burkina Faso | 780(685 to 883) | 16(14.46 to 17.73) | 1963(1736 to 2197) | 18.9(17.12 to 20.82) | 0.2(0.14 to 0.22) |
| Cameroon | 854(755 to 961) | 16.5(14.89 to 18.27) | 2667(2381 to 2984) | 20.1(18.2 to 22.32) | 0.2(0.18 to 0.27) |
| Cabo Verde | 41(37 to 46) | 15.9(14.36 to 17.68) | 107(96 to 119) | 24(21.46 to 26.87) | 0.5(0.43 to 0.59) |
| Chad | 510(451 to 572) | 15.2(13.79 to 16.92) | 1281(1128 to 1443) | 18.4(16.63 to 20.36) | 0.2(0.17 to 0.25) |
| Cote d'Ivoire | 879(765 to 1000) | 16.7(15.14 to 18.56) | 2350(2078 to 2643) | 19.7(17.85 to 21.79) | 0.2(0.14 to 0.23) |
| Gambia | 74(65 to 84) | 16(14.5 to 17.79) | 214(191 to 240) | 19(17.1 to 21.17) | 0.2(0.14 to 0.23) |
| Ghana | 1380(1229 to 1549) | 21(18.94 to 23.44) | 3935(3560 to 4388) | 25.9(23.26 to 28.85) | 0.2(0.18 to 0.29) |
| Guinea | 577(513 to 647) | 15.9(14.32 to 17.57) | 1193(1066 to 1331) | 18.8(16.96 to 20.88) | 0.2(0.15 to 0.23) |
| Guinea-Bissau | 77(67 to 87) | 15.3(13.88 to 17.01) | 154(136 to 174) | 17.5(15.86 to 19.44) | 0.1(0.11 to 0.18) |
| Liberia | 192(171 to 216) | 16.5(14.89 to 18.25) | 447(396 to 504) | 18.7(16.86 to 20.83) | 0.1(0.1 to 0.18) |
| Mali | 723(640 to 815) | 15.5(13.99 to 17.25) | 1868(1653 to 2095) | 18.6(16.84 to 20.66) | 0.2(0.16 to 0.25) |
| Mauritania | 184(163 to 206) | 16(14.45 to 17.76) | 441(395 to 489) | 19.8(17.89 to 21.97) | 0.2(0.19 to 0.29) |
| Niger | 569(496 to 644) | 15.5(14.02 to 17.25) | 1719(1504 to 1946) | 18(16.36 to 19.93) | 0.2(0.12 to 0.2) |
| Nigeria | 8392(7450 to 9422) | 16.7(15.11 to 18.58) | 20292(18076 to 22625) | 20.6(18.51 to 22.91) | 0.2(0.2 to 0.27) |
| Sao Tome and Principe | 11(10 to 13) | 15.8(14.27 to 17.58) | 22(20 to 25) | 19.4(17.53 to 21.53) | 0.2(0.18 to 0.27) |
| Senegal | 630(558 to 706) | 16.4(14.79 to 18.16) | 1565(1398 to 1747) | 19.3(17.49 to 21.52) | 0.2(0.14 to 0.22) |
| Sierra Leone | 345(307 to 384) | 15.9(14.34 to 17.55) | 769(682 to 864) | 18.4(16.73 to 20.43) | 0.2(0.12 to 0.2) |
| Togo | 266(232 to 302) | 16.2(14.63 to 17.98) | 724(641 to 815) | 18.4(16.65 to 20.38) | 0.1(0.1 to 0.18) |
| Eastern Sub-Saharan Africa | 14557(12717 to 16565) | 14.3(12.8 to 16.12) | 33309(29098 to 37929) | 14.8(13.27 to 16.7) | 0(0.03 to 0.05) |
| Burundi | 434(380 to 495) | 13.9(12.45 to 15.75) | 919(802 to 1047) | 14.8(13.23 to 16.75) | 0.1(0.04 to 0.09) |
| Comoros | 39(35 to 44) | 14.1(12.5 to 15.81) | 79(71 to 90) | 14.6(13.06 to 16.49) | 0(0.01 to 0.07) |
| Djibouti | 33(29 to 38) | 14.2(12.63 to 15.98) | 111(96 to 126) | 14.9(13.29 to 16.8) | 0.1(0.02 to 0.08) |
| Eritrea | 203(175 to 232) | 13.8(12.27 to 15.48) | 528(459 to 606) | 14.3(12.81 to 16.2) | 0(0.01 to 0.07) |
| Ethiopia | 3957(3450 to 4522) | 14.5(12.95 to 16.35) | 8959(7855 to 10240) | 15.1(13.51 to 17.05) | 0(0.02 to 0.06) |
| Kenya | 1776(1552 to 2025) | 14.8(13.29 to 16.68) | 4484(3933 to 5110) | 15.3(13.74 to 17.27) | 0(0.03 to 0.04) |
| Madagascar | 951(834 to 1080) | 14.2(12.68 to 16.01) | 2133(1856 to 2435) | 14.8(13.17 to 16.69) | 0(0.01 to 0.07) |
| Malawi | 728(637 to 830) | 14.2(12.58 to 16) | 1473(1288 to 1681) | 14.7(13.11 to 16.66) | 0(0.01 to 0.07) |
| Mozambique | 1045(919 to 1185) | 13.9(12.38 to 15.74) | 2190(1911 to 2497) | 14.3(12.78 to 16.03) | 0(0 to 0.05) |
| Rwanda | 542(473 to 617) | 14(12.48 to 15.68) | 1102(970 to 1252) | 14.5(12.96 to 16.31) | 0(0.01 to 0.07) |
| Somalia | 509(441 to 581) | 14.2(12.6 to 15.95) | 1439(1241 to 1648) | 14.6(13.01 to 16.44) | 0(0 to 0.06) |
| South Sudan | 470(410 to 535) | 14.4(12.84 to 16.26) | 746(651 to 845) | 14.8(13.21 to 16.71) | 0(0 to 0.06) |
| United Republic of Tanzania | 2014(1767 to 2297) | 14.1(12.54 to 15.88) | 4709(4135 to 5334) | 14.7(13.09 to 16.52) | 0(0.02 to 0.07) |
| Uganda | 1273(1114 to 1452) | 14.1(12.63 to 16.01) | 3010(2617 to 3426) | 14.6(13 to 16.38) | 0(0 to 0.06) |
| Zambia | 572(499 to 654) | 14.1(12.57 to 15.97) | 1400(1225 to 1599) | 14.6(13.03 to 16.35) | 0(0 to 0.06) |
| Central Sub-Saharan Africa | 4158(3625 to 4715) | 15.4(13.92 to 17.08) | 10366(9107 to 11680) | 15.7(14.14 to 17.42) | 0(0 to 0.04) |
| Angola | 726(630 to 827) | 14.1(12.68 to 15.63) | 2216(1930 to 2511) | 15.6(14 to 17.29) | 0.1(0.07 to 0.14) |
| Central African Republic | 198(173 to 224) | 13.6(12.29 to 15.11) | 392(340 to 445) | 14.2(12.81 to 15.75) | 0(0.02 to 0.07) |
| Congo | 189(165 to 214) | 14.9(13.44 to 16.54) | 480(425 to 540) | 16.4(14.78 to 18.18) | 0.1(0.07 to 0.13) |
| Democratic Republic of the Congo | 2918(2547 to 3293) | 16(14.38 to 17.66) | 6971(6131 to 7863) | 15.7(14.1 to 17.41) | 0(-0.05 to 0.01) |
| Equatorial Guinea | 32(28 to 36) | 13.3(12.01 to 14.77) | 115(101 to 131) | 17.5(15.78 to 19.46) | 0.3(0.27 to 0.37) |
| Gabon | 95(85 to 107) | 15.9(14.36 to 17.66) | 192(172 to 214) | 17.6(15.82 to 19.63) | 0.1(0.07 to 0.15) |

**Table S3: YLDs of decubitus ulcer in 1990 and 2019 for both sexes and percentage change of age-standardized rates (ASR) by location.**

| **Regions or countries** | **1990** | | **2019** | | **Percentage change in ASR from 1990 and 2019** |
| --- | --- | --- | --- | --- | --- |
|  | **Counts (95% UI)** | **Rate (95% UI)** | **Counts (95% UI)** | **Rate (95% UI)** |  |
| Global | 64857(45376 to 85486) | 1.9(1.36 to 2.51) | 130238(92478 to 171036) | 1.7(1.21 to 2.24) | -0.1(-0.12 to -0.08) |
| High-income North America | 20378(14441 to 26666) | 5.7(4.04 to 7.49) | 31877(22897 to 41435) | 5.1(3.66 to 6.64) | -0.1(-0.15 to -0.06) |
| Canada | 1503(1037 to 2057) | 4.8(3.31 to 6.57) | 3009(2074 to 4129) | 4.5(3.14 to 6.17) | -0.1(-0.19 to 0.1) |
| Greenland | 1(1 to 2) | 4(2.79 to 5.39) | 3(2 to 3) | 4.2(2.92 to 5.68) | 0(-0.1 to 0.21) |
| United States of America | 18873(13348 to 24711) | 5.8(4.1 to 7.58) | 28865(20699 to 37373) | 5.2(3.72 to 6.71) | -0.1(-0.15 to -0.05) |
| Australasia | 460(319 to 618) | 2.1(1.47 to 2.81) | 961(663 to 1301) | 1.9(1.32 to 2.58) | -0.1(-0.2 to 0.04) |
| Australia | 386(266 to 522) | 2.1(1.48 to 2.84) | 818(559 to 1121) | 1.9(1.31 to 2.61) | -0.1(-0.23 to 0.06) |
| New Zealand | 74(51 to 102) | 2(1.39 to 2.72) | 143(99 to 193) | 1.9(1.27 to 2.48) | -0.1(-0.21 to 0.08) |
| High-income Asia Pacific | 3958(2725 to 5326) | 2.2(1.52 to 2.96) | 9408(6611 to 12710) | 2.2(1.51 to 2.95) | 0(-0.04 to 0.03) |
| Brunei Darussalam | 3(2 to 4) | 2.8(1.94 to 3.77) | 8(5 to 10) | 3(2.02 to 3.98) | 0.1(-0.07 to 0.21) |
| Japan | 3249(2257 to 4370) | 2.2(1.51 to 2.94) | 7107(5017 to 9546) | 2.1(1.45 to 2.83) | 0(-0.08 to 0) |
| Singapore | 65(45 to 88) | 3.2(2.23 to 4.26) | 206(141 to 283) | 2.9(2.03 to 4.04) | -0.1(-0.2 to 0.05) |
| Republic of Korea | 641(435 to 872) | 2.2(1.48 to 2.9) | 2087(1424 to 2844) | 2.7(1.83 to 3.63) | 0.2(0.08 to 0.41) |
| Western Europe | 15452(10825 to 20511) | 2.8(1.98 to 3.67) | 25199(17931 to 33741) | 2.7(1.88 to 3.54) | 0(-0.09 to 0) |
| Andorra | 1(1 to 2) | 2.5(1.77 to 3.42) | 4(3 to 5) | 2.5(1.77 to 3.4) | 0(-0.11 to 0.15) |
| Austria | 337(226 to 466) | 3(2 to 4.05) | 512(358 to 696) | 2.9(2.04 to 3.93) | 0(-0.16 to 0.15) |
| Belgium | 698(472 to 927) | 4.7(3.23 to 6.26) | 1052(737 to 1405) | 4.3(2.98 to 5.76) | -0.1(-0.2 to 0.04) |
| Cyprus | 17(12 to 24) | 2.6(1.8 to 3.6) | 50(34 to 69) | 3(2.08 to 4.02) | 0.1(-0.03 to 0.3) |
| Denmark | 145(96 to 202) | 1.9(1.24 to 2.57) | 227(158 to 309) | 2.1(1.44 to 2.79) | 0.1(-0.04 to 0.3) |
| Finland | 185(125 to 254) | 2.8(1.88 to 3.8) | 347(239 to 474) | 3(2.09 to 4.09) | 0.1(-0.06 to 0.27) |
| France | 3181(2190 to 4365) | 3.7(2.56 to 5.05) | 4533(3113 to 6248) | 2.9(2.02 to 4.01) | -0.2(-0.32 to -0.1) |
| Germany | 2867(1974 to 3972) | 2.4(1.65 to 3.26) | 5609(3958 to 7644) | 2.9(2.04 to 3.92) | 0.2(0.03 to 0.41) |
| Greece | 293(198 to 409) | 2.1(1.45 to 2.92) | 537(369 to 738) | 2.3(1.56 to 3.1) | 0.1(-0.07 to 0.26) |
| Iceland | 6(4 to 9) | 2.2(1.46 to 2.99) | 15(10 to 20) | 2.7(1.88 to 3.66) | 0.3(0.09 to 0.48) |
| Ireland | 118(81 to 163) | 3.1(2.11 to 4.16) | 175(123 to 238) | 2.4(1.71 to 3.31) | -0.2(-0.32 to -0.07) |
| Israel | 142(95 to 193) | 3.1(2.08 to 4.14) | 563(395 to 754) | 4.6(3.21 to 6.16) | 0.5(0.3 to 0.75) |
| Italy | 1743(1231 to 2328) | 2.1(1.5 to 2.81) | 2741(1962 to 3621) | 1.7(1.19 to 2.24) | -0.2(-0.24 to -0.15) |
| Luxembourg | 12(8 to 16) | 2.4(1.64 to 3.21) | 24(16 to 32) | 2.4(1.61 to 3.13) | 0(-0.14 to 0.14) |
| Malta | 22(16 to 30) | 5.7(4.04 to 7.76) | 53(37 to 71) | 5.6(3.83 to 7.45) | 0(-0.16 to 0.13) |
| Monaco | 2(1 to 2) | 2.2(1.5 to 2.98) | 2(2 to 3) | 2.4(1.65 to 3.27) | 0.1(-0.06 to 0.26) |
| Netherlands | 717(487 to 976) | 3.6(2.49 to 4.98) | 1033(710 to 1412) | 3(2.09 to 4.13) | -0.2(-0.28 to -0.04) |
| Norway | 244(170 to 323) | 3.8(2.63 to 4.96) | 357(253 to 472) | 3.8(2.68 to 5.04) | 0(-0.02 to 0.07) |
| San Marino | 1(0 to 1) | 2.1(1.41 to 2.83) | 1(1 to 2) | 2.3(1.59 to 3.08) | 0.1(-0.06 to 0.27) |
| Portugal | 221(150 to 303) | 1.8(1.22 to 2.39) | 448(306 to 622) | 1.8(1.28 to 2.54) | 0(-0.14 to 0.25) |
| Spain | 1488(1031 to 2028) | 2.9(2.04 to 3.99) | 3211(2178 to 4384) | 3(2.06 to 4.08) | 0(-0.12 to 0.2) |
| Sweden | 406(279 to 553) | 2.7(1.88 to 3.65) | 485(340 to 655) | 2.4(1.64 to 3.18) | -0.1(-0.23 to -0.03) |
| Switzerland | 203(138 to 277) | 2(1.37 to 2.72) | 412(282 to 572) | 2.3(1.58 to 3.15) | 0.2(-0.01 to 0.34) |
| United Kingdom | 2389(1671 to 3149) | 2.8(1.93 to 3.62) | 2785(1948 to 3711) | 2.3(1.62 to 3.13) | -0.1(-0.19 to -0.11) |
| Southern Latin America | 823(561 to 1113) | 2(1.36 to 2.69) | 2611(1837 to 3541) | 3(2.14 to 4.12) | 0.5(0.34 to 0.77) |
| Argentina | 555(373 to 769) | 1.9(1.29 to 2.64) | 1753(1216 to 2371) | 3.2(2.19 to 4.26) | 0.6(0.38 to 0.99) |
| Chile | 148(101 to 206) | 1.7(1.17 to 2.33) | 628(437 to 855) | 2.7(1.85 to 3.62) | 0.6(0.34 to 0.87) |
| Uruguay | 120(82 to 166) | 3.2(2.16 to 4.32) | 229(160 to 311) | 3.6(2.5 to 4.9) | 0.1(-0.01 to 0.33) |
| Eastern Europe | 3177(2139 to 4315) | 1.2(0.83 to 1.65) | 4065(2794 to 5511) | 1.3(0.89 to 1.75) | 0.1(0.02 to 0.11) |
| Belarus | 151(102 to 210) | 1.2(0.84 to 1.7) | 187(125 to 258) | 1.3(0.87 to 1.76) | 0(-0.1 to 0.19) |
| Estonia | 24(16 to 33) | 1.2(0.82 to 1.7) | 31(21 to 44) | 1.3(0.88 to 1.78) | 0.1(-0.08 to 0.23) |
| Latvia | 51(35 to 70) | 1.5(1.05 to 2.11) | 69(48 to 96) | 1.9(1.3 to 2.56) | 0.2(0.04 to 0.43) |
| Lithuania | 42(28 to 57) | 1(0.65 to 1.31) | 55(36 to 77) | 1(0.69 to 1.41) | 0.1(-0.05 to 0.22) |
| Republic of Moldova | 52(36 to 72) | 1.2(0.86 to 1.72) | 69(46 to 96) | 1.3(0.87 to 1.79) | 0(-0.09 to 0.19) |
| Russian Federation | 2029(1377 to 2760) | 1.2(0.81 to 1.62) | 2770(1904 to 3745) | 1.3(0.89 to 1.74) | 0.1(0.06 to 0.11) |
| Ukraine | 827(546 to 1127) | 1.3(0.83 to 1.71) | 885(591 to 1215) | 1.3(0.85 to 1.76) | 0(-0.11 to 0.16) |
| Central Europe | 2022(1396 to 2732) | 1.5(1.05 to 2.03) | 3785(2650 to 5013) | 1.9(1.3 to 2.49) | 0.2(0.19 to 0.32) |
| Albania | 28(19 to 38) | 1.3(0.88 to 1.76) | 55(37 to 77) | 1.4(0.94 to 1.92) | 0.1(-0.07 to 0.28) |
| Bosnia and Herzegovina | 47(32 to 64) | 1.3(0.86 to 1.74) | 74(50 to 104) | 1.4(0.96 to 1.95) | 0.1(-0.05 to 0.3) |
| Bulgaria | 138(92 to 193) | 1.3(0.88 to 1.76) | 185(124 to 254) | 1.4(0.94 to 1.87) | 0.1(-0.08 to 0.25) |
| Croatia | 73(49 to 99) | 1.3(0.86 to 1.71) | 121(81 to 167) | 1.5(0.99 to 2.01) | 0.2(-0.02 to 0.35) |
| Czechia | 177(117 to 245) | 1.4(0.94 to 1.91) | 388(266 to 531) | 1.9(1.32 to 2.62) | 0.4(0.2 to 0.65) |
| Hungary | 168(114 to 230) | 1.2(0.86 to 1.69) | 268(184 to 369) | 1.5(1.01 to 1.99) | 0.2(-0.01 to 0.38) |
| North Macedonia | 23(15 to 31) | 1.3(0.91 to 1.83) | 39(26 to 54) | 1.4(0.96 to 1.92) | 0(-0.11 to 0.23) |
| Montenegro | 8(5 to 11) | 1.3(0.88 to 1.84) | 12(9 to 17) | 1.4(0.97 to 1.96) | 0.1(-0.09 to 0.24) |
| Poland | 859(594 to 1158) | 2.1(1.45 to 2.8) | 1787(1256 to 2373) | 2.6(1.85 to 3.52) | 0.3(0.17 to 0.37) |
| Romania | 297(202 to 417) | 1.2(0.83 to 1.68) | 476(319 to 659) | 1.3(0.9 to 1.85) | 0.1(-0.03 to 0.28) |
| Serbia | 87(58 to 118) | 0.9(0.58 to 1.19) | 156(107 to 214) | 1.1(0.75 to 1.45) | 0.2(0.05 to 0.49) |
| Slovakia | 87(59 to 120) | 1.5(1.04 to 2.1) | 153(102 to 209) | 1.8(1.22 to 2.48) | 0.2(0.01 to 0.4) |
| Slovenia | 31(21 to 42) | 1.3(0.92 to 1.82) | 71(48 to 98) | 1.6(1.12 to 2.25) | 0.2(0.04 to 0.45) |
| Central Asia | 121(81 to 165) | 0.3(0.17 to 0.35) | 187(125 to 255) | 0.3(0.19 to 0.38) | 0.1(0.06 to 0.11) |
| Armenia | 7(4 to 9) | 0.3(0.18 to 0.36) | 11(7 to 15) | 0.3(0.19 to 0.38) | 0.1(0.03 to 0.1) |
| Azerbaijan | 13(9 to 18) | 0.3(0.17 to 0.35) | 23(15 to 31) | 0.3(0.19 to 0.38) | 0.1(0.05 to 0.13) |
| Georgia | 15(10 to 20) | 0.3(0.17 to 0.35) | 17(12 to 24) | 0.3(0.2 to 0.4) | 0.1(0.08 to 0.21) |
| Kazakhstan | 31(20 to 42) | 0.2(0.17 to 0.34) | 43(29 to 59) | 0.3(0.18 to 0.37) | 0.1(0.06 to 0.15) |
| Kyrgyzstan | 8(5 to 11) | 0.3(0.17 to 0.35) | 12(8 to 17) | 0.3(0.18 to 0.38) | 0.1(0.03 to 0.11) |
| Mongolia | 3(2 to 4) | 0.3(0.17 to 0.36) | 6(4 to 8) | 0.3(0.18 to 0.37) | 0.1(0.02 to 0.1) |
| Tajikistan | 8(6 to 11) | 0.3(0.18 to 0.37) | 14(9 to 19) | 0.3(0.19 to 0.39) | 0.1(0.02 to 0.1) |
| Turkmenistan | 5(3 to 7) | 0.3(0.17 to 0.35) | 10(7 to 14) | 0.3(0.19 to 0.38) | 0.1(0.04 to 0.12) |
| Uzbekistan | 32(21 to 43) | 0.3(0.18 to 0.36) | 51(33 to 70) | 0.3(0.19 to 0.38) | 0.1(0.03 to 0.11) |
| Central Latin America | 3381(2344 to 4462) | 4.1(2.83 to 5.35) | 9436(6588 to 12481) | 4.2(2.92 to 5.5) | 0(-0.02 to 0.07) |
| Colombia | 775(536 to 1052) | 4.6(3.23 to 6.26) | 2328(1593 to 3147) | 4.3(2.93 to 5.82) | -0.1(-0.2 to 0.07) |
| Costa Rica | 82(57 to 110) | 4.7(3.25 to 6.28) | 229(160 to 306) | 4.5(3.14 to 6.08) | 0(-0.15 to 0.11) |
| El Salvador | 96(66 to 130) | 3(2.07 to 4.08) | 235(163 to 316) | 3.7(2.56 to 5.05) | 0.2(0.06 to 0.44) |
| Guatemala | 111(76 to 151) | 3(2.02 to 4.03) | 370(254 to 503) | 3.3(2.29 to 4.48) | 0.1(-0.04 to 0.31) |
| Honduras | 72(49 to 100) | 3.2(2.15 to 4.33) | 184(127 to 249) | 3(2.1 to 4.08) | -0.1(-0.19 to 0.11) |
| Mexico | 1794(1247 to 2362) | 4.2(2.96 to 5.54) | 4774(3353 to 6279) | 4.4(3.1 to 5.8) | 0(0 to 0.08) |
| Nicaragua | 56(38 to 76) | 3.3(2.26 to 4.49) | 141(96 to 192) | 3.3(2.28 to 4.51) | 0(-0.14 to 0.18) |
| Panama | 70(49 to 94) | 4.7(3.33 to 6.39) | 222(154 to 294) | 5.3(3.65 to 6.98) | 0.1(-0.02 to 0.29) |
| Venezuela (Bolivarian Republic of) | 324(224 to 440) | 3.2(2.21 to 4.28) | 952(656 to 1300) | 3.5(2.39 to 4.75) | 0.1(-0.06 to 0.3) |
| Andean Latin America | 257(174 to 349) | 1.2(0.8 to 1.62) | 778(535 to 1060) | 1.4(0.96 to 1.9) | 0.2(0.07 to 0.31) |
| Bolivia (Plurinational State of) | 38(25 to 52) | 1.1(0.73 to 1.51) | 107(73 to 146) | 1.2(0.82 to 1.68) | 0.1(-0.03 to 0.27) |
| Ecuador | 66(45 to 91) | 1.1(0.78 to 1.6) | 239(163 to 320) | 1.6(1.12 to 2.19) | 0.4(0.22 to 0.68) |
| Peru | 153(104 to 209) | 1.2(0.82 to 1.65) | 431(292 to 593) | 1.3(0.89 to 1.82) | 0.1(-0.06 to 0.28) |
| Caribbean | 744(518 to 985) | 3(2.08 to 3.93) | 1711(1203 to 2282) | 3.3(2.33 to 4.41) | 0.1(0.04 to 0.2) |
| Antigua and Barbuda | 2(1 to 3) | 3.4(2.34 to 4.64) | 5(3 to 6) | 5.4(3.69 to 7.25) | 0.6(0.35 to 0.82) |
| Bahamas | 8(5 to 10) | 5.4(3.76 to 7.36) | 21(15 to 29) | 6.2(4.25 to 8.33) | 0.1(-0.01 to 0.31) |
| Barbados | 25(17 to 33) | 8.2(5.75 to 10.83) | 40(28 to 54) | 8.4(5.85 to 11.24) | 0(-0.1 to 0.15) |
| Belize | 3(2 to 4) | 3.2(2.18 to 4.34) | 9(6 to 12) | 3.5(2.42 to 4.69) | 0.1(-0.05 to 0.27) |
| Bermuda | 2(1 to 2) | 2.8(1.93 to 3.81) | 5(4 to 7) | 4(2.79 to 5.32) | 0.4(0.23 to 0.67) |
| Cuba | 234(159 to 314) | 2.3(1.58 to 3.08) | 552(376 to 740) | 2.9(1.97 to 3.87) | 0.3(0.06 to 0.47) |
| Dominica | 4(3 to 5) | 5.2(3.59 to 7.1) | 5(4 to 7) | 5.9(4.13 to 7.86) | 0.1(-0.01 to 0.27) |
| Dominican Republic | 81(55 to 112) | 2.1(1.43 to 2.91) | 217(147 to 295) | 2.4(1.63 to 3.24) | 0.1(-0.03 to 0.35) |
| Grenada | 3(2 to 4) | 3.4(2.32 to 4.66) | 6(4 to 7) | 5.8(4.08 to 7.81) | 0.7(0.47 to 1) |
| Guyana | 8(6 to 11) | 2.1(1.43 to 2.8) | 15(10 to 21) | 2.9(1.94 to 3.9) | 0.4(0.16 to 0.63) |
| Haiti | 66(45 to 89) | 2.2(1.48 to 2.91) | 176(121 to 244) | 2.8(1.93 to 3.87) | 0.3(0.12 to 0.53) |
| Jamaica | 92(64 to 125) | 5(3.47 to 6.78) | 186(131 to 250) | 5.7(4.02 to 7.72) | 0.2(-0.01 to 0.32) |
| Puerto Rico | 156(107 to 213) | 4.5(3.11 to 6.12) | 307(214 to 415) | 3.9(2.71 to 5.2) | -0.1(-0.25 to 0) |
| Saint Kitts and Nevis | 2(2 to 3) | 6.5(4.51 to 8.73) | 3(2 to 4) | 5.9(4.08 to 8.03) | -0.1(-0.2 to 0.04) |
| Saint Lucia | 3(2 to 4) | 3.4(2.43 to 4.6) | 10(7 to 14) | 5(3.43 to 6.75) | 0.4(0.25 to 0.69) |
| Saint Vincent and the Grenadines | 3(2 to 4) | 4.1(2.85 to 5.52) | 7(5 to 9) | 5.4(3.75 to 7.16) | 0.3(0.14 to 0.52) |
| Suriname | 8(6 to 11) | 3.2(2.22 to 4.35) | 27(19 to 36) | 4.9(3.43 to 6.61) | 0.5(0.32 to 0.79) |
| Trinidad and Tobago | 18(12 to 25) | 2.1(1.44 to 2.91) | 53(36 to 72) | 3.1(2.12 to 4.17) | 0.4(0.22 to 0.69) |
| United States Virgin Islands | 3(2 to 4) | 4.1(2.83 to 5.64) | 9(6 to 12) | 5.2(3.58 to 7) | 0.3(0.09 to 0.47) |
| Tropical Latin America | 2987(2039 to 3983) | 3.2(2.27 to 4.29) | 8695(6076 to 11543) | 3.7(2.62 to 4.96) | 0.2(0.09 to 0.22) |
| Brazil | 2936(2005 to 3916) | 3.3(2.29 to 4.33) | 8551(5971 to 11368) | 3.8(2.64 to 5) | 0.2(0.09 to 0.22) |
| Paraguay | 51(34 to 68) | 2.1(1.44 to 2.86) | 144(99 to 192) | 2.6(1.78 to 3.47) | 0.2(0.04 to 0.45) |
| East Asia | 5197(3556 to 7039) | 0.7(0.5 to 0.96) | 14633(10148 to 19455) | 0.9(0.61 to 1.16) | 0.2(0.14 to 0.29) |
| China | 4790(3257 to 6498) | 0.7(0.46 to 0.91) | 13261(9178 to 17696) | 0.8(0.57 to 1.07) | 0.2(0.12 to 0.29) |
| Democratic People's Republic of Korea | 104(70 to 140) | 0.9(0.58 to 1.19) | 286(193 to 400) | 1.1(0.73 to 1.47) | 0.2(0.03 to 0.47) |
| Taiwan (Province of China) | 304(212 to 414) | 2.6(1.79 to 3.51) | 1087(750 to 1461) | 2.7(1.88 to 3.65) | 0.1(-0.1 to 0.26) |
| Southeast Asia | 1373(939 to 1847) | 0.6(0.45 to 0.87) | 5092(3532 to 6741) | 1(0.72 to 1.38) | 0.6(0.5 to 0.73) |
| Cambodia | 19(13 to 26) | 0.5(0.36 to 0.71) | 73(49 to 101) | 0.8(0.53 to 1.08) | 0.5(0.29 to 0.81) |
| Indonesia | 407(276 to 555) | 0.5(0.34 to 0.66) | 1037(707 to 1404) | 0.6(0.41 to 0.8) | 0.2(0.14 to 0.3) |
| Lao People's Democratic Republic | 9(6 to 12) | 0.5(0.36 to 0.73) | 27(18 to 37) | 0.8(0.55 to 1.11) | 0.5(0.28 to 0.8) |
| Malaysia | 78(53 to 106) | 1(0.69 to 1.39) | 460(315 to 620) | 2.1(1.42 to 2.82) | 1.1(0.7 to 1.48) |
| Maldives | 1(0 to 1) | 0.9(0.6 to 1.19) | 3(2 to 4) | 1.3(0.92 to 1.79) | 0.5(0.26 to 0.86) |
| Mauritius | 5(4 to 7) | 0.9(0.61 to 1.26) | 21(15 to 29) | 1.4(0.94 to 1.86) | 0.5(0.22 to 0.85) |
| Myanmar | 113(78 to 155) | 0.6(0.42 to 0.84) | 344(236 to 465) | 0.9(0.64 to 1.28) | 0.5(0.28 to 0.8) |
| Philippines | 287(198 to 377) | 1.2(0.87 to 1.64) | 1087(756 to 1435) | 1.8(1.24 to 2.32) | 0.4(0.37 to 0.48) |
| Sri Lanka | 47(32 to 64) | 0.5(0.35 to 0.71) | 155(104 to 215) | 0.7(0.49 to 1) | 0.4(0.19 to 0.66) |
| Seychelles | 0(0 to 1) | 0.7(0.47 to 0.98) | 1(1 to 1) | 1(0.66 to 1.36) | 0.4(0.15 to 0.73) |
| Thailand | 214(147 to 294) | 0.8(0.51 to 1.05) | 1321(901 to 1808) | 1.4(0.92 to 1.87) | 0.8(0.45 to 1.24) |
| Timor-Leste | 1(1 to 2) | 0.5(0.35 to 0.71) | 5(3 to 7) | 0.8(0.53 to 1.06) | 0.5(0.27 to 0.8) |
| Viet Nam | 190(130 to 264) | 0.5(0.35 to 0.73) | 552(378 to 758) | 0.7(0.49 to 1) | 0.4(0.17 to 0.7) |
| Oceania | 13(9 to 18) | 0.6(0.44 to 0.85) | 39(27 to 52) | 0.8(0.57 to 1.08) | 0.3(0.14 to 0.41) |
| American Samoa | 0(0 to 0) | 2(1.37 to 2.67) | 1(1 to 1) | 2.7(1.88 to 3.68) | 0.4(0.19 to 0.65) |
| Cook Islands | 0(0 to 0) | 0.8(0.57 to 1.11) | 0(0 to 0) | 1(0.7 to 1.42) | 0.2(0.02 to 0.51) |
| Micronesia (Federated States of) | 0(0 to 0) | 0.7(0.45 to 0.97) | 0(0 to 1) | 1(0.66 to 1.34) | 0.4(0.15 to 0.79) |
| Fiji | 2(1 to 3) | 0.8(0.54 to 1.08) | 5(3 to 7) | 1.1(0.73 to 1.45) | 0.4(0.1 to 0.7) |
| Guam | 0(0 to 1) | 0.8(0.54 to 1.09) | 2(1 to 3) | 1(0.69 to 1.4) | 0.3(0.04 to 0.61) |
| Kiribati | 0(0 to 0) | 0.8(0.52 to 1.1) | 0(0 to 1) | 1(0.67 to 1.38) | 0.2(-0.01 to 0.55) |
| Nauru | 0(0 to 0) | 0.8(0.51 to 1.08) | 0(0 to 0) | 0.9(0.62 to 1.24) | 0.2(-0.05 to 0.43) |
| Niue | 0(0 to 0) | 0.9(0.61 to 1.29) | 0(0 to 0) | 1.1(0.74 to 1.49) | 0.2(-0.04 to 0.46) |
| Marshall Islands | 0(0 to 0) | 0.7(0.49 to 1.01) | 0(0 to 0) | 0.9(0.62 to 1.25) | 0.2(0.01 to 0.54) |
| Northern Mariana Islands | 0(0 to 0) | 1.1(0.73 to 1.53) | 0(0 to 0) | 1.2(0.8 to 1.63) | 0.1(-0.1 to 0.3) |
| Palau | 0(0 to 0) | 0.6(0.39 to 0.84) | 0(0 to 0) | 0.7(0.45 to 0.95) | 0.1(-0.1 to 0.42) |
| Papua New Guinea | 7(5 to 10) | 0.5(0.36 to 0.73) | 23(16 to 31) | 0.7(0.46 to 0.92) | 0.3(0.05 to 0.5) |
| Samoa | 1(0 to 1) | 0.8(0.54 to 1.1) | 1(1 to 2) | 1(0.69 to 1.38) | 0.3(0.03 to 0.59) |
| Solomon Islands | 1(0 to 1) | 0.6(0.39 to 0.77) | 2(1 to 2) | 0.8(0.5 to 1.02) | 0.3(0.13 to 0.62) |
| Tokelau | 0(0 to 0) | 0.7(0.48 to 0.98) | 0(0 to 0) | 0.9(0.61 to 1.24) | 0.2(0 to 0.52) |
| Tonga | 0(0 to 0) | 0.7(0.5 to 1.03) | 1(1 to 1) | 1(0.7 to 1.41) | 0.4(0.11 to 0.69) |
| Tuvalu | 0(0 to 0) | 0.8(0.52 to 1.08) | 0(0 to 0) | 0.9(0.61 to 1.24) | 0.2(-0.07 to 0.47) |
| Vanuatu | 0(0 to 0) | 0.6(0.38 to 0.77) | 1(1 to 1) | 0.7(0.5 to 1) | 0.3(0.08 to 0.58) |
| North Africa and Middle East | 888(605 to 1201) | 0.5(0.37 to 0.73) | 2606(1799 to 3505) | 0.7(0.48 to 0.92) | 0.3(0.18 to 0.33) |
| Afghanistan | 31(21 to 42) | 0.5(0.32 to 0.65) | 68(45 to 95) | 0.5(0.34 to 0.7) | 0.1(-0.01 to 0.16) |
| Algeria | 59(40 to 80) | 0.5(0.36 to 0.72) | 178(120 to 242) | 0.6(0.43 to 0.87) | 0.2(0.07 to 0.38) |
| Bahrain | 1(1 to 1) | 0.5(0.36 to 0.7) | 5(3 to 7) | 0.7(0.48 to 0.96) | 0.4(0.17 to 0.58) |
| Egypt | 136(91 to 186) | 0.5(0.34 to 0.69) | 313(210 to 424) | 0.6(0.39 to 0.79) | 0.2(0.03 to 0.29) |
| Iran (Islamic Republic of) | 129(88 to 176) | 0.5(0.35 to 0.69) | 430(296 to 583) | 0.6(0.45 to 0.88) | 0.3(0.21 to 0.34) |
| Iraq | 40(27 to 55) | 0.5(0.33 to 0.68) | 122(84 to 167) | 0.6(0.39 to 0.8) | 0.2(0.04 to 0.3) |
| Jordan | 9(6 to 12) | 0.7(0.48 to 0.98) | 45(30 to 61) | 0.9(0.59 to 1.17) | 0.2(0.01 to 0.46) |
| Kuwait | 4(2 to 5) | 0.5(0.37 to 0.75) | 15(10 to 20) | 0.6(0.41 to 0.85) | 0.1(-0.03 to 0.29) |
| Lebanon | 10(7 to 14) | 0.5(0.36 to 0.71) | 32(22 to 45) | 0.6(0.44 to 0.88) | 0.2(0.08 to 0.4) |
| Libya | 10(7 to 14) | 0.5(0.35 to 0.72) | 28(19 to 38) | 0.6(0.4 to 0.82) | 0.1(0.03 to 0.29) |
| Morocco | 64(44 to 88) | 0.5(0.33 to 0.67) | 152(102 to 205) | 0.6(0.38 to 0.76) | 0.2(0.05 to 0.27) |
| Palestine | 4(3 to 6) | 0.5(0.32 to 0.67) | 13(8 to 17) | 0.6(0.38 to 0.77) | 0.2(0.05 to 0.29) |
| Oman | 4(2 to 5) | 0.5(0.37 to 0.72) | 12(8 to 16) | 0.7(0.48 to 0.99) | 0.4(0.18 to 0.58) |
| Qatar | 1(1 to 1) | 0.5(0.38 to 0.74) | 7(4 to 9) | 0.8(0.52 to 1.05) | 0.4(0.19 to 0.62) |
| Saudi Arabia | 57(39 to 78) | 1.1(0.75 to 1.5) | 261(179 to 357) | 2.1(1.5 to 2.88) | 0.9(0.59 to 1.34) |
| Sudan | 46(31 to 62) | 0.5(0.33 to 0.66) | 103(70 to 140) | 0.5(0.37 to 0.75) | 0.1(0.02 to 0.25) |
| Syrian Arab Republic | 28(19 to 38) | 0.5(0.35 to 0.72) | 59(40 to 80) | 0.6(0.39 to 0.78) | 0.1(0.01 to 0.27) |
| Tunisia | 24(16 to 33) | 0.5(0.36 to 0.72) | 69(47 to 95) | 0.6(0.43 to 0.85) | 0.2(0.07 to 0.37) |
| Turkey | 203(138 to 276) | 0.6(0.4 to 0.81) | 599(412 to 809) | 0.7(0.5 to 0.99) | 0.2(0.06 to 0.45) |
| United Arab Emirates | 3(2 to 4) | 0.6(0.38 to 0.76) | 23(15 to 32) | 0.7(0.47 to 0.95) | 0.2(0.05 to 0.43) |
| Yemen | 24(16 to 33) | 0.5(0.32 to 0.66) | 71(47 to 97) | 0.5(0.35 to 0.72) | 0.1(0 to 0.2) |
| South Asia | 1364(899 to 1859) | 0.2(0.16 to 0.33) | 3969(2664 to 5405) | 0.3(0.21 to 0.42) | 0.3(0.25 to 0.32) |
| Bangladesh | 119(79 to 162) | 0.2(0.16 to 0.32) | 367(247 to 498) | 0.3(0.22 to 0.44) | 0.3(0.22 to 0.51) |
| Bhutan | 1(0 to 1) | 0.2(0.15 to 0.3) | 1(1 to 2) | 0.3(0.19 to 0.38) | 0.3(0.2 to 0.36) |
| India | 1081(711 to 1474) | 0.2(0.17 to 0.34) | 3244(2184 to 4426) | 0.3(0.21 to 0.43) | 0.3(0.24 to 0.31) |
| Nepal | 21(14 to 29) | 0.2(0.15 to 0.31) | 59(40 to 81) | 0.3(0.2 to 0.4) | 0.3(0.21 to 0.38) |
| Pakistan | 143(95 to 194) | 0.2(0.15 to 0.31) | 297(196 to 407) | 0.3(0.18 to 0.36) | 0.2(0.12 to 0.19) |
| Southern Sub-Saharan Africa | 560(386 to 750) | 1.9(1.35 to 2.56) | 1081(758 to 1441) | 2(1.44 to 2.7) | 0.1(0 to 0.11) |
| Botswana | 10(7 to 14) | 1.7(1.12 to 2.24) | 26(17 to 35) | 2(1.38 to 2.72) | 0.2(0.02 to 0.43) |
| Lesotho | 15(10 to 21) | 1.5(0.97 to 2) | 21(14 to 28) | 1.6(1.11 to 2.26) | 0.1(-0.06 to 0.3) |
| Namibia | 13(9 to 18) | 1.7(1.15 to 2.31) | 27(19 to 37) | 1.9(1.32 to 2.57) | 0.1(-0.03 to 0.31) |
| South Africa | 438(302 to 586) | 2(1.41 to 2.67) | 871(612 to 1151) | 2.1(1.48 to 2.8) | 0(-0.02 to 0.11) |
| Eswatini | 6(4 to 8) | 1.6(1.07 to 2.14) | 10(7 to 14) | 1.8(1.2 to 2.38) | 0.1(-0.05 to 0.29) |
| Zimbabwe | 78(53 to 105) | 1.7(1.14 to 2.27) | 126(85 to 171) | 1.7(1.16 to 2.27) | 0(-0.14 to 0.16) |
| Western Sub-Saharan Africa | 800(541 to 1090) | 0.8(0.52 to 1.02) | 1996(1357 to 2734) | 0.9(0.62 to 1.21) | 0.2(0.15 to 0.22) |
| Benin | 19(13 to 26) | 0.7(0.49 to 0.98) | 51(34 to 69) | 0.8(0.56 to 1.12) | 0.1(0.03 to 0.28) |
| Burkina Faso | 37(25 to 50) | 0.7(0.49 to 0.98) | 92(62 to 127) | 0.8(0.57 to 1.15) | 0.2(0.04 to 0.29) |
| Cameroon | 40(27 to 55) | 0.7(0.51 to 1) | 124(84 to 171) | 0.9(0.61 to 1.19) | 0.2(0.05 to 0.34) |
| Cabo Verde | 2(1 to 3) | 0.7(0.49 to 0.99) | 5(3 to 6) | 1(0.72 to 1.44) | 0.4(0.26 to 0.62) |
| Chad | 24(16 to 33) | 0.7(0.48 to 0.95) | 60(40 to 82) | 0.8(0.55 to 1.09) | 0.2(0.05 to 0.28) |
| Cote d'Ivoire | 42(28 to 58) | 0.8(0.52 to 1.02) | 110(75 to 152) | 0.9(0.59 to 1.17) | 0.1(0 to 0.31) |
| Gambia | 4(2 to 5) | 0.7(0.5 to 1) | 10(7 to 14) | 0.8(0.58 to 1.15) | 0.2(0.05 to 0.29) |
| Ghana | 64(43 to 88) | 0.9(0.61 to 1.25) | 179(120 to 243) | 1.1(0.76 to 1.52) | 0.2(0.05 to 0.41) |
| Guinea | 27(18 to 37) | 0.7(0.49 to 0.99) | 55(37 to 75) | 0.8(0.56 to 1.14) | 0.1(0.05 to 0.26) |
| Guinea-Bissau | 4(2 to 5) | 0.7(0.48 to 0.96) | 7(5 to 10) | 0.8(0.53 to 1.05) | 0.1(0.02 to 0.21) |
| Liberia | 9(6 to 12) | 0.7(0.51 to 1.01) | 21(14 to 29) | 0.8(0.56 to 1.12) | 0.1(0 to 0.25) |
| Mali | 34(23 to 47) | 0.7(0.49 to 0.98) | 88(59 to 122) | 0.8(0.56 to 1.13) | 0.2(0.07 to 0.28) |
| Mauritania | 9(6 to 12) | 0.7(0.49 to 1.01) | 20(14 to 28) | 0.9(0.6 to 1.19) | 0.2(0.08 to 0.34) |
| Niger | 27(18 to 38) | 0.7(0.48 to 0.97) | 82(55 to 112) | 0.8(0.55 to 1.09) | 0.1(0.03 to 0.23) |
| Nigeria | 399(272 to 545) | 0.8(0.52 to 1.03) | 949(649 to 1308) | 0.9(0.63 to 1.24) | 0.2(0.15 to 0.24) |
| Sao Tome and Principe | 1(0 to 1) | 0.7(0.49 to 0.99) | 1(1 to 1) | 0.9(0.58 to 1.16) | 0.2(0.06 to 0.33) |
| Senegal | 30(20 to 40) | 0.7(0.5 to 1.01) | 72(49 to 100) | 0.9(0.58 to 1.17) | 0.2(0.03 to 0.29) |
| Sierra Leone | 16(11 to 22) | 0.7(0.5 to 0.98) | 36(25 to 49) | 0.8(0.56 to 1.11) | 0.1(0.03 to 0.24) |
| Togo | 13(9 to 18) | 0.7(0.5 to 1.01) | 34(23 to 47) | 0.8(0.56 to 1.11) | 0.1(-0.01 to 0.26) |
| Eastern Sub-Saharan Africa | 702(475 to 978) | 0.7(0.46 to 0.94) | 1612(1096 to 2229) | 0.7(0.48 to 0.97) | 0(0.03 to 0.05) |
| Burundi | 21(14 to 29) | 0.7(0.45 to 0.91) | 44(30 to 62) | 0.7(0.47 to 0.97) | 0.1(0.04 to 0.1) |
| Comoros | 2(1 to 3) | 0.7(0.45 to 0.92) | 4(3 to 5) | 0.7(0.47 to 0.96) | 0(0.01 to 0.07) |
| Djibouti | 2(1 to 2) | 0.7(0.46 to 0.94) | 5(4 to 7) | 0.7(0.48 to 0.98) | 0.1(0.02 to 0.09) |
| Eritrea | 10(7 to 14) | 0.7(0.44 to 0.89) | 26(17 to 35) | 0.7(0.46 to 0.93) | 0(0.02 to 0.08) |
| Ethiopia | 190(128 to 263) | 0.7(0.46 to 0.95) | 433(292 to 598) | 0.7(0.49 to 1) | 0(0.03 to 0.07) |
| Kenya | 86(58 to 119) | 0.7(0.48 to 0.98) | 217(147 to 302) | 0.7(0.5 to 1) | 0(0.02 to 0.04) |
| Madagascar | 46(31 to 64) | 0.7(0.46 to 0.94) | 103(70 to 143) | 0.7(0.48 to 0.97) | 0(0.01 to 0.07) |
| Malawi | 35(24 to 49) | 0.7(0.46 to 0.93) | 71(48 to 98) | 0.7(0.47 to 0.97) | 0(0.01 to 0.07) |
| Mozambique | 50(34 to 69) | 0.7(0.45 to 0.9) | 106(72 to 148) | 0.7(0.46 to 0.94) | 0(0 to 0.06) |
| Rwanda | 26(18 to 36) | 0.7(0.45 to 0.91) | 53(36 to 74) | 0.7(0.47 to 0.95) | 0(0.01 to 0.08) |
| Somalia | 25(17 to 34) | 0.7(0.45 to 0.92) | 69(47 to 96) | 0.7(0.47 to 0.95) | 0(0 to 0.06) |
| South Sudan | 23(15 to 32) | 0.7(0.46 to 0.95) | 36(24 to 50) | 0.7(0.47 to 0.98) | 0(0 to 0.06) |
| United Republic of Tanzania | 97(66 to 135) | 0.7(0.45 to 0.92) | 228(154 to 314) | 0.7(0.47 to 0.96) | 0(0.01 to 0.08) |
| Uganda | 61(42 to 85) | 0.7(0.46 to 0.93) | 146(98 to 201) | 0.7(0.47 to 0.96) | 0(0 to 0.07) |
| Zambia | 28(19 to 38) | 0.7(0.45 to 0.92) | 68(46 to 94) | 0.7(0.47 to 0.96) | 0(0 to 0.07) |
| Central Sub-Saharan Africa | 199(135 to 274) | 0.7(0.48 to 0.97) | 496(336 to 684) | 0.7(0.49 to 0.97) | 0(-0.05 to 0.09) |
| Angola | 35(23 to 48) | 0.7(0.45 to 0.9) | 107(72 to 147) | 0.7(0.49 to 0.98) | 0.1(0.04 to 0.15) |
| Central African Republic | 9(6 to 13) | 0.6(0.43 to 0.87) | 19(13 to 26) | 0.7(0.45 to 0.9) | 0(0.01 to 0.08) |
| Congo | 9(6 to 13) | 0.7(0.46 to 0.94) | 23(15 to 31) | 0.8(0.51 to 1.02) | 0.1(0.01 to 0.18) |
| Democratic Republic of the Congo | 140(94 to 191) | 0.7(0.49 to 1) | 333(226 to 457) | 0.7(0.49 to 0.97) | 0(-0.1 to 0.08) |
| Equatorial Guinea | 2(1 to 2) | 0.6(0.42 to 0.85) | 5(4 to 8) | 0.8(0.53 to 1.07) | 0.3(0.18 to 0.4) |
| Gabon | 5(3 to 6) | 0.7(0.5 to 1) | 9(6 to 12) | 0.8(0.54 to 1.09) | 0.1(-0.01 to 0.2) |
