# Supplementary material for: The global burden of decubitus ulcers from 1990 to 2019
Source: Sci Rep. 2021 Nov 5;11:21750. doi: 10.1038/s41598-021-01188-4 (PMC8571371; doi:10.1038/s41598-021-01188-4)
Supplement: Supplementary file 2 — Supplementary Figures. [file 41598_2021_1188_MOESM2_ESM.pdf]

Additional file 2

The global burden of decubitus ulcers from 1990 to 2019

List of supplemental Figure lengends

Figure S1: The percentage change in age-standardized point prevalence (A) and incidence (B) of decubitus ulcer from 1990 to 2019 for 21 GBD regions by sex……………………………**3**

Figure S2: Number of prevalent cases of decubitus ulcer from 1990 to 2019 for 21 GBD regions…………………………………………………………………………….....**4**

Figure S3: The percentage change in age-standardized point prevalence (A) and incidence (B) of decubitus ulcer from 1990 to 2019 for country and territory…………………….…….**5**

Figure S4: Number of incident cases of decubitus ulcer from 1990 to 2019 for 21 GBD regions……………………………………………………………….…………....**6**

Figure S5: The age-standardized years lived with disability (YLDs) rate in 2019 for 21 GBD regions, by sex…………………………………………….……………………....**7**

Figure S6: The percentage change in age-standardized point YLDs of decubitus ulcer from 1990 to 2019 for 21 GBD regions by sex. …….……………………………..…………....**8**

Figure S7: Age-standardized YLDs rates of decubitus ulcer per 100,000 population in 2019, by country and territory. ……………………….……………………...……………..………....**9**

Figure S8: The percentage change in age-standardized point YLDs of decubitus ulcer from 1990 to 2019 for country and territory. ……….……………….……….................……....**10**

Figure S9: Global number of incident cases and incidence rate of decubitus ulcer per 100,000 populations by age and sex, 2019, Shading indicate the 95% upper and lower uncertainty intervals (95% UIs) for the incident rate, respectively. ….……..…………………….....…....**11**

Figure S10: Global number of YLDs rate of decubitus ulcer per 100,000 populations by age and sex, 2019, Shading indicate the 95% upper and lower uncertainty intervals (95% UIs) for the YLDs rate, respectively. ……………………….………..……………………..….........**12**

Figure S11: Association of age-standardized incidence rate due to decubitus ulcer and

sociodemographic index (SDI) for 21 regions in the GBD study. Expected values based on Socio-demographic Index and disease rates in all locations are shown as the black line. Different points are plotted for each GBD region and show observed age-standardized incidence rates from 1990 to 2019. ……………………….……..………………………….....**13**

Figure S12: Association of age-standardized prevalence rate due to decubitus ulcer and sociodemographic index (SDI) for 21 regions in the GBD study. Expected values based on Socio-demographic Index and disease rates in all locations are shown as the black line. Different points are plotted for each GBD region and show observed age-standardized prevalence rates from 1990 to 2019. …………….…………...…………………………....**14**

Figure S13: Association of age-standardized incidence rate due to decubits ulcer and Sociodemographic Index for 204 countries and territories. Expected values based on Socio-demographic Index and disease rates in all locations are shown as the black line. Each point shows the observed incidence rate per country in 2019. ………..………………........……....**15**

Figure S14: Association of age-standardized prevalence rate due to decubitus ulcer and Sociodemographic Index for 204 countries and territories. Expected values based on Socio-demographic Index and disease rates in all locations are shown as the black line. Each point shows the observed prevalence rate per country in 2019. ……………………..…...……....**16**

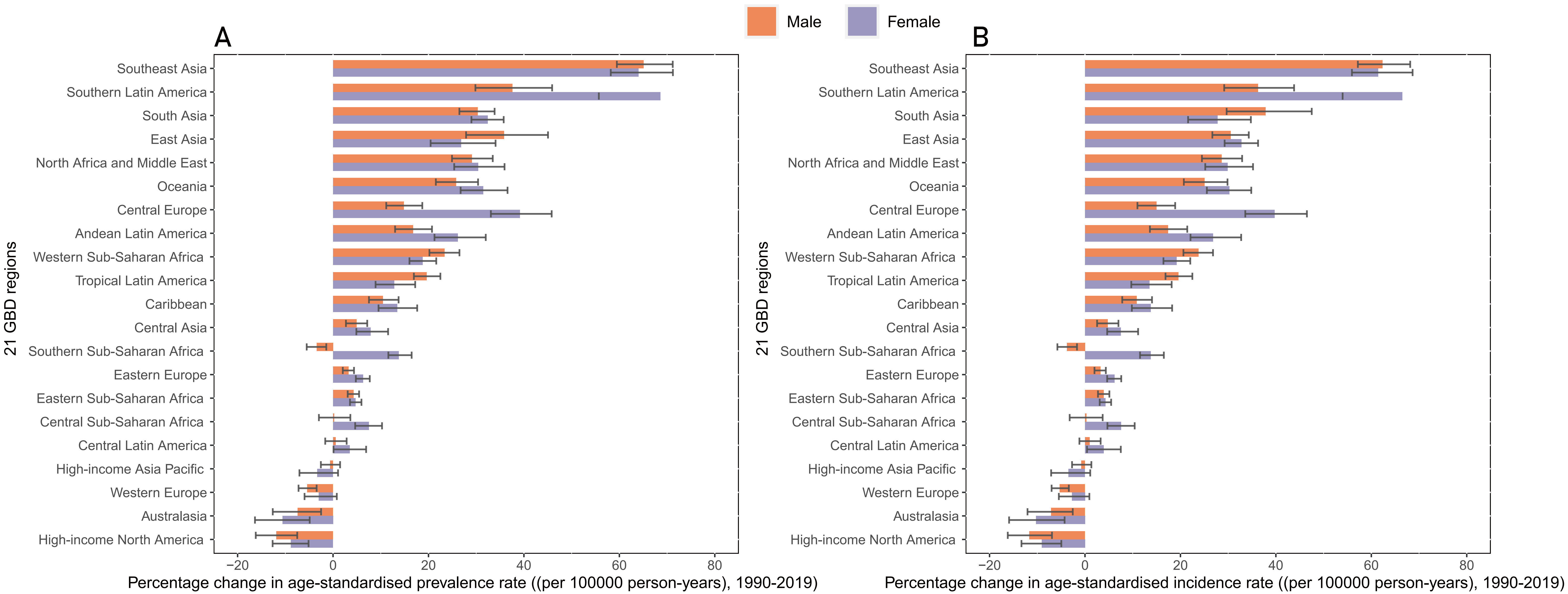

Figure S1: The percentage change in age-standardized point prevalence (A) and incidence (B) of decubitus ulcer from 1990 to 2019 for 21 GBD regions by sex.

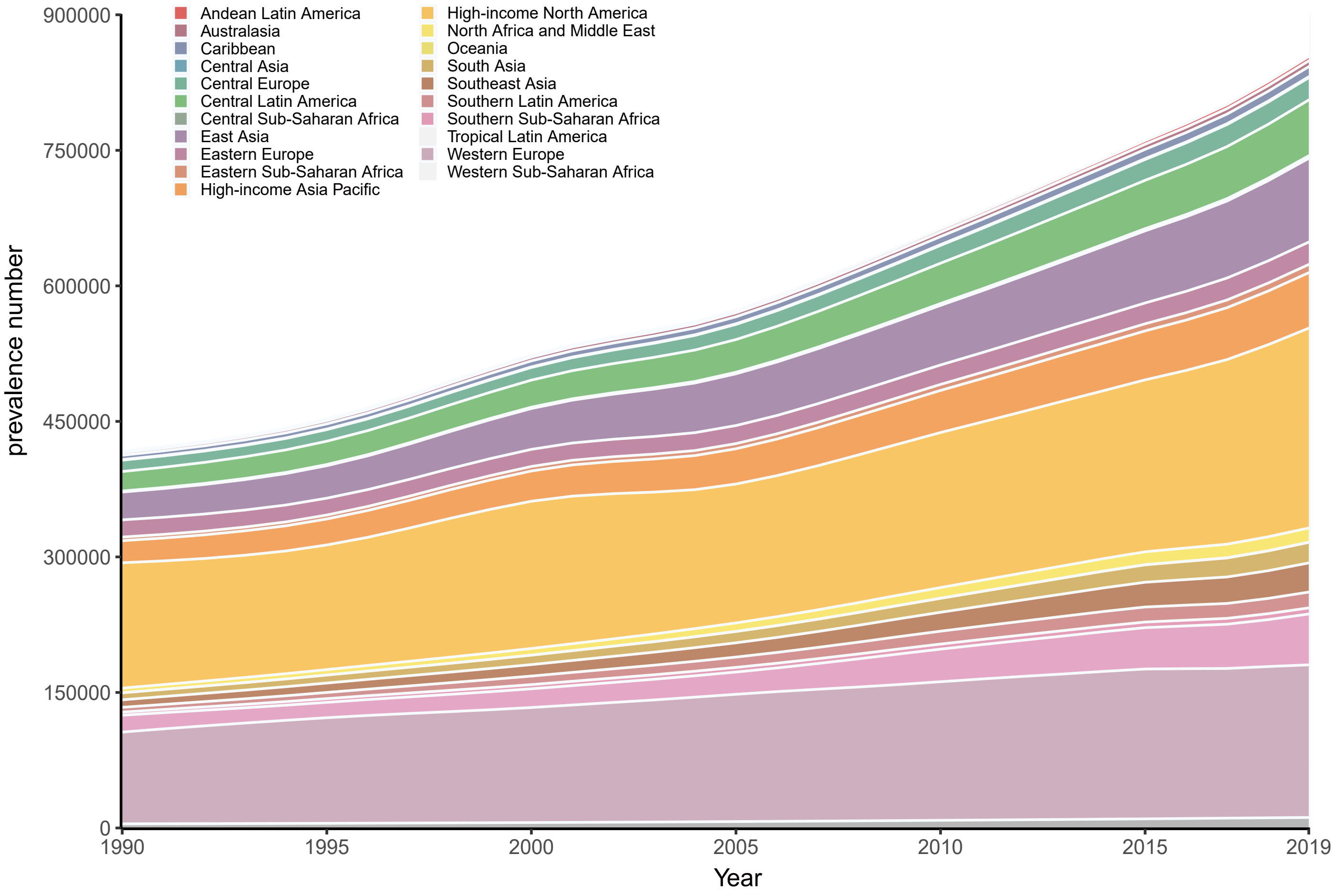

Figure S2: Number of prevalent cases of decubitus ulcer from 1990 to 2019 for 21 GBD regions.

A

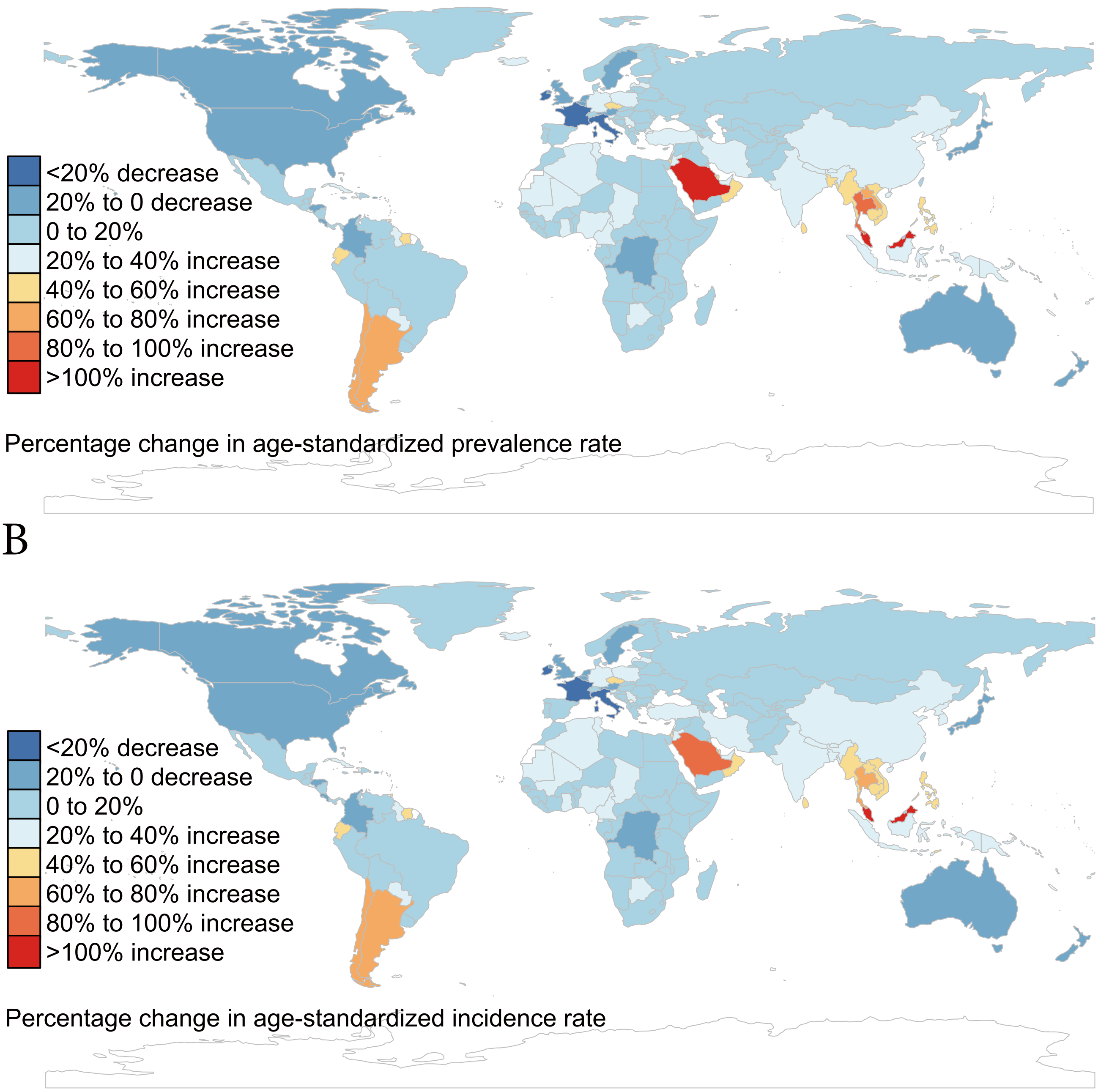

B

Figure S3: The percentage change in age-standardized point prevalence (A) and incidence (B) of decubitus ulcer from 1990 to 2019 for country and territory.These pictures were generated by R software version 3.6.3 (https://cran.r-project.org/doc/FAQ/R-FAQ.html#Citing-R) and visualized using the ggplot2 3.3.0 package [8].

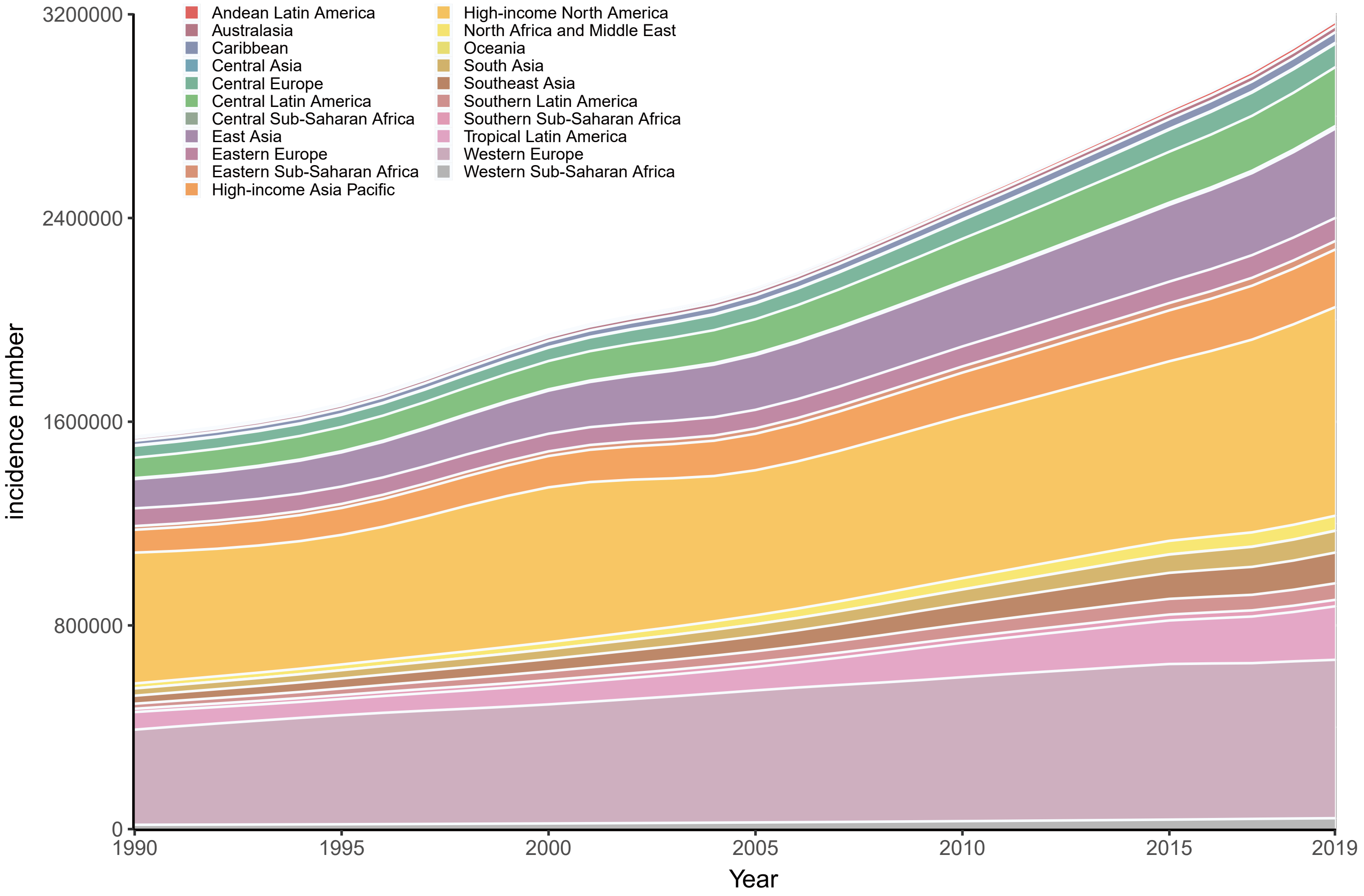

Figure S4: Number of incident cases of decubitus ulcer from 1990 to 2019 for 21 GBD regions.

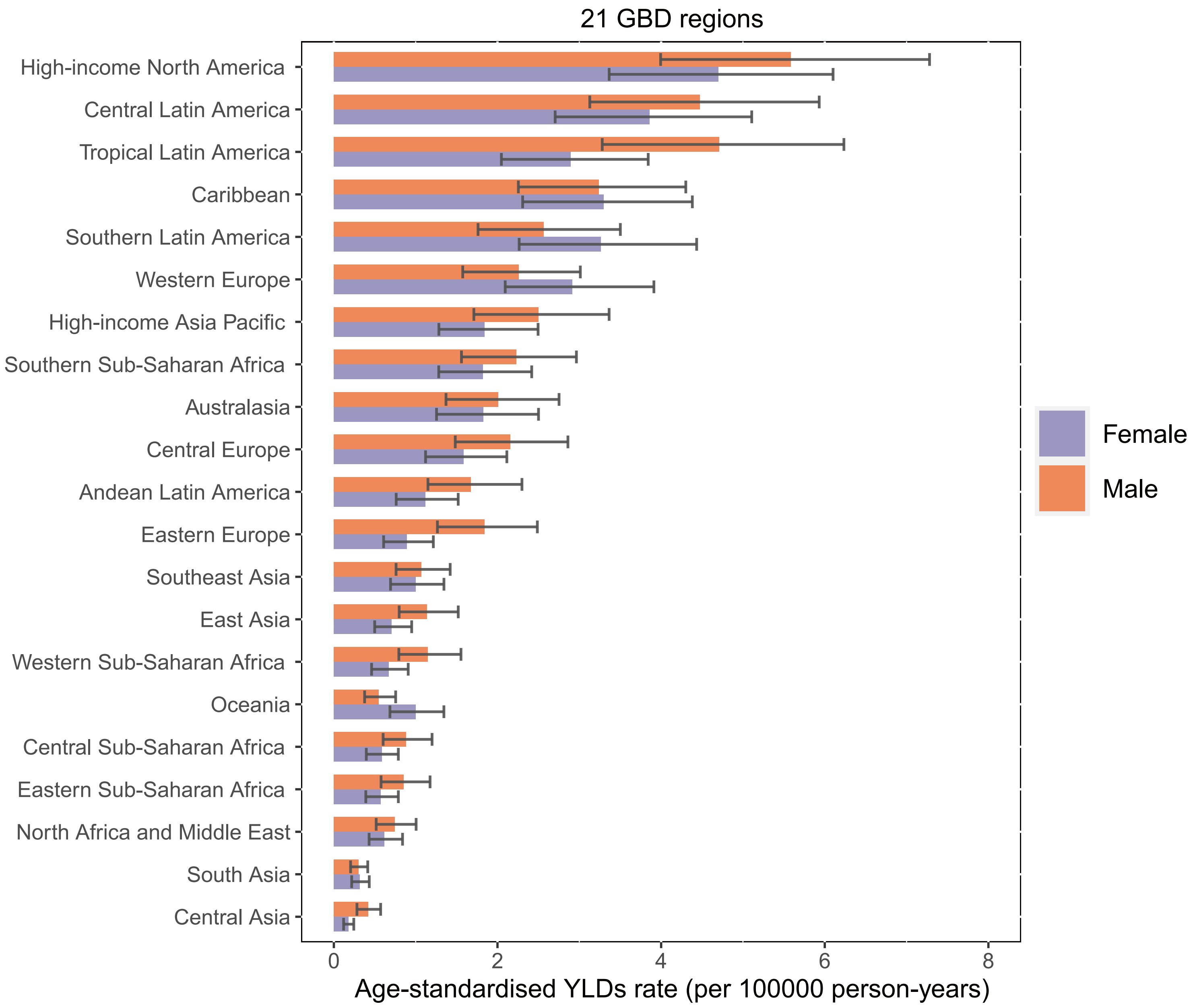

Figure S5: The age-standardized years lived with disability (YLDs) rate in 2019 for 21 GBD regions, by sex.

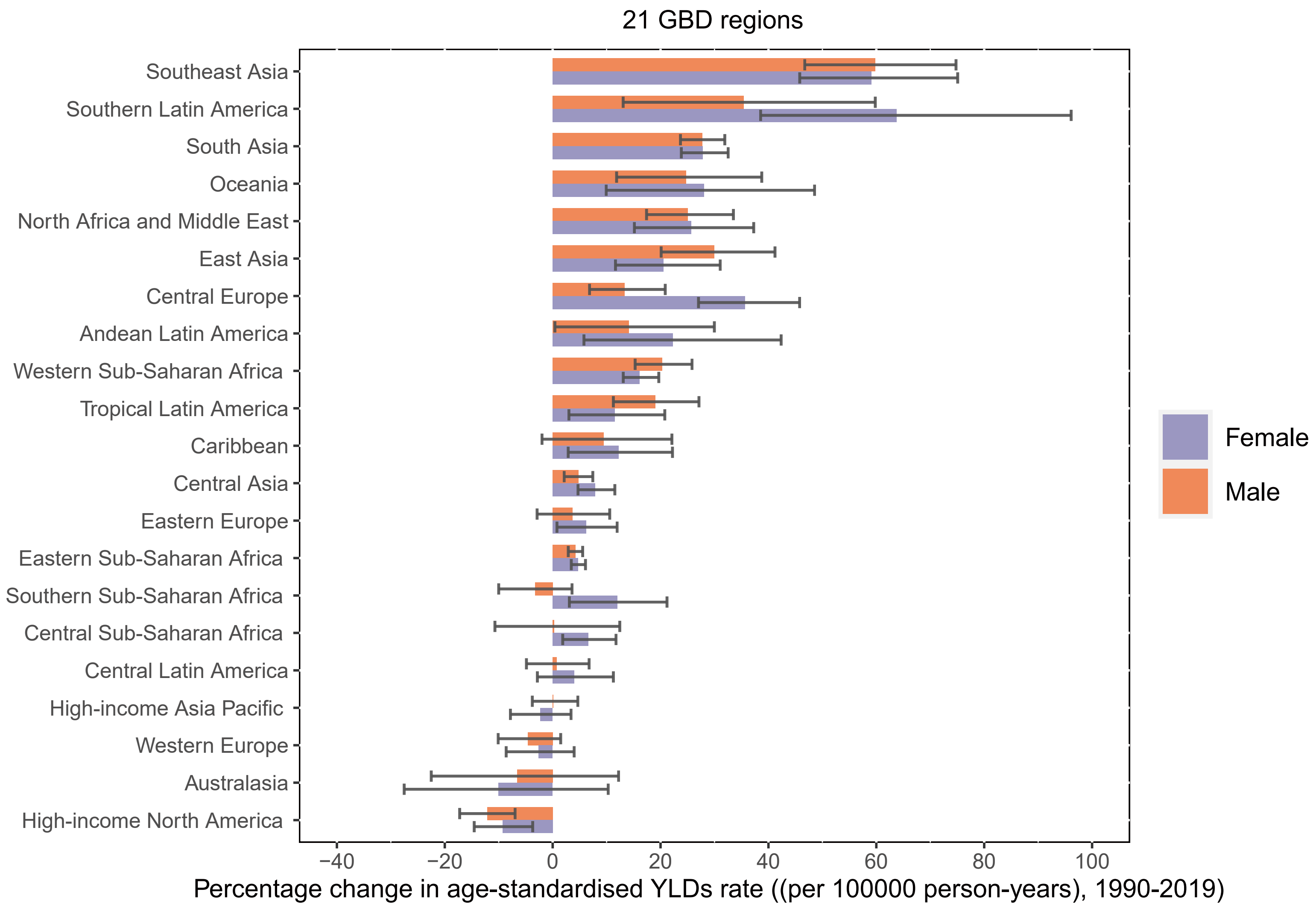

Figure S6: The percentage change in age-standardized point YLDs of decubitus ulcer from 1990 to 2019 for 21 GBD regions by sex.

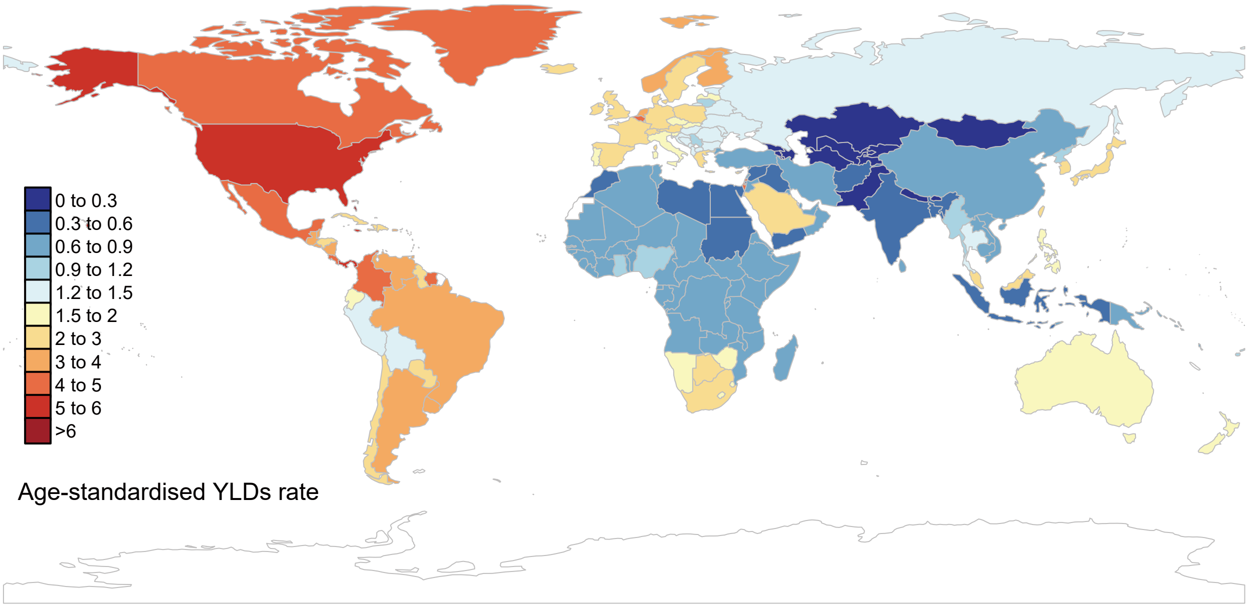

Figure S7: Age-standardized YLDs rates of decubitus ulcer per 100,000 population in 2019, by country and territory. This picture was generated by R software version 3.6.3 (https://cran.r-project.org/doc/FAQ/R-FAQ.html#Citing-R) and visualized using the ggplot2 3.3.0 package [8].

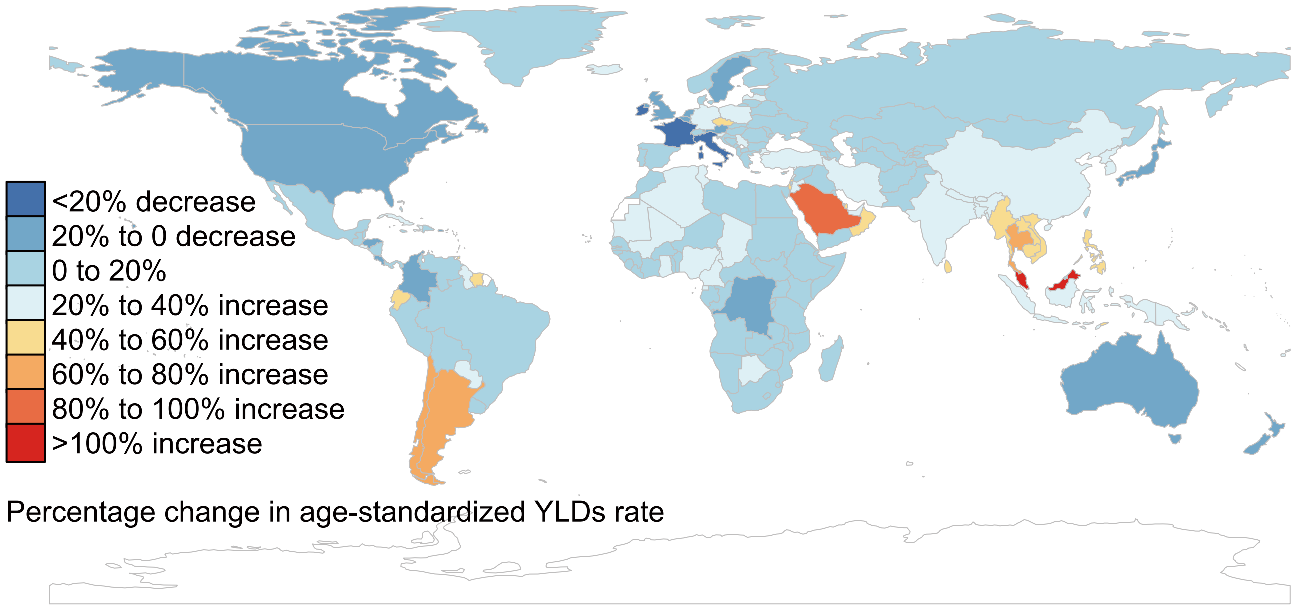

Figure S8: The percentage change in age-standardized point YLDs of decubitus ulcer from 1990 to 2019 for country and territory. This picture was generated by R software version 3.6.3 (https://cran.r-project.org/doc/FAQ/R-FAQ.html#Citing-R) and visualized using the ggplot2 3.3.0 package [8].

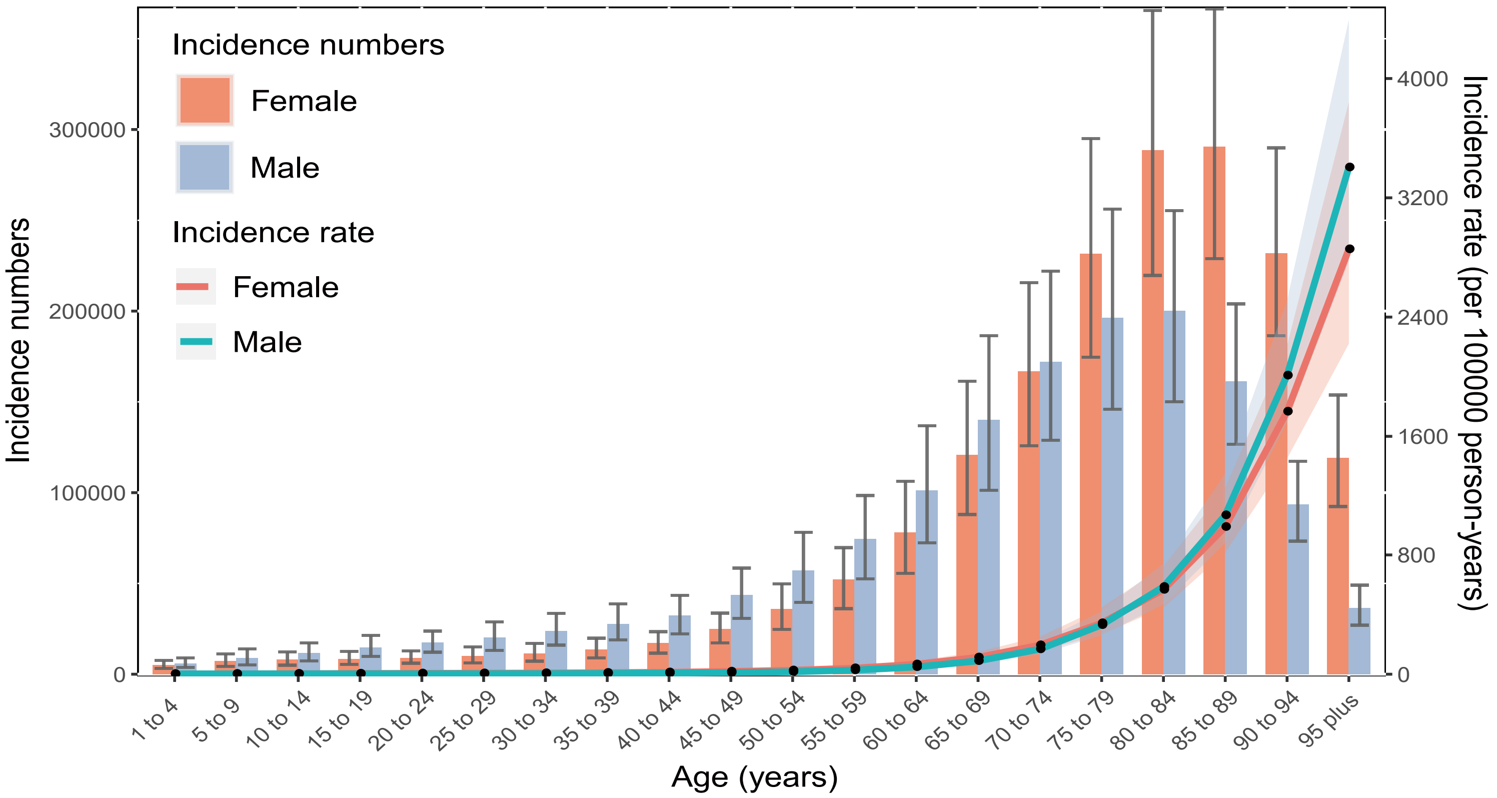

Figure S9: Global number of incident cases and incidence rate of decubitus ulcer per 100,000 populations by age and sex, 2019, Shading indicate the 95% upper and lower uncertainty intervals (95% UIs) for the incident rate, respectively.

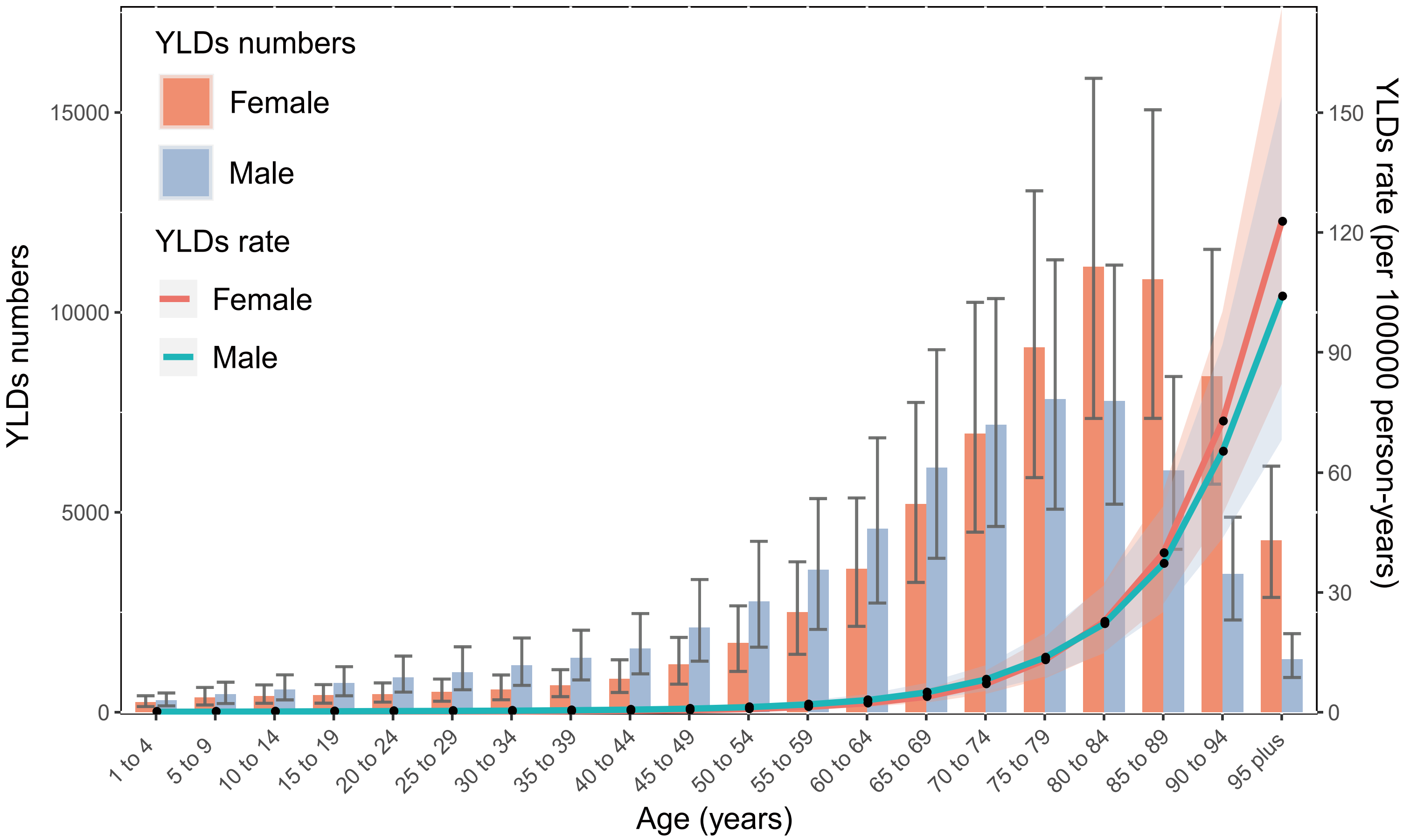

Figure S10: Global number of YLDs rate of decubitus ulcer per 100,000 populations by age and sex, 2019, Shading indicate the 95% upper and lower uncertainty intervals (95% UIs) for the YLDs rate, respectively.

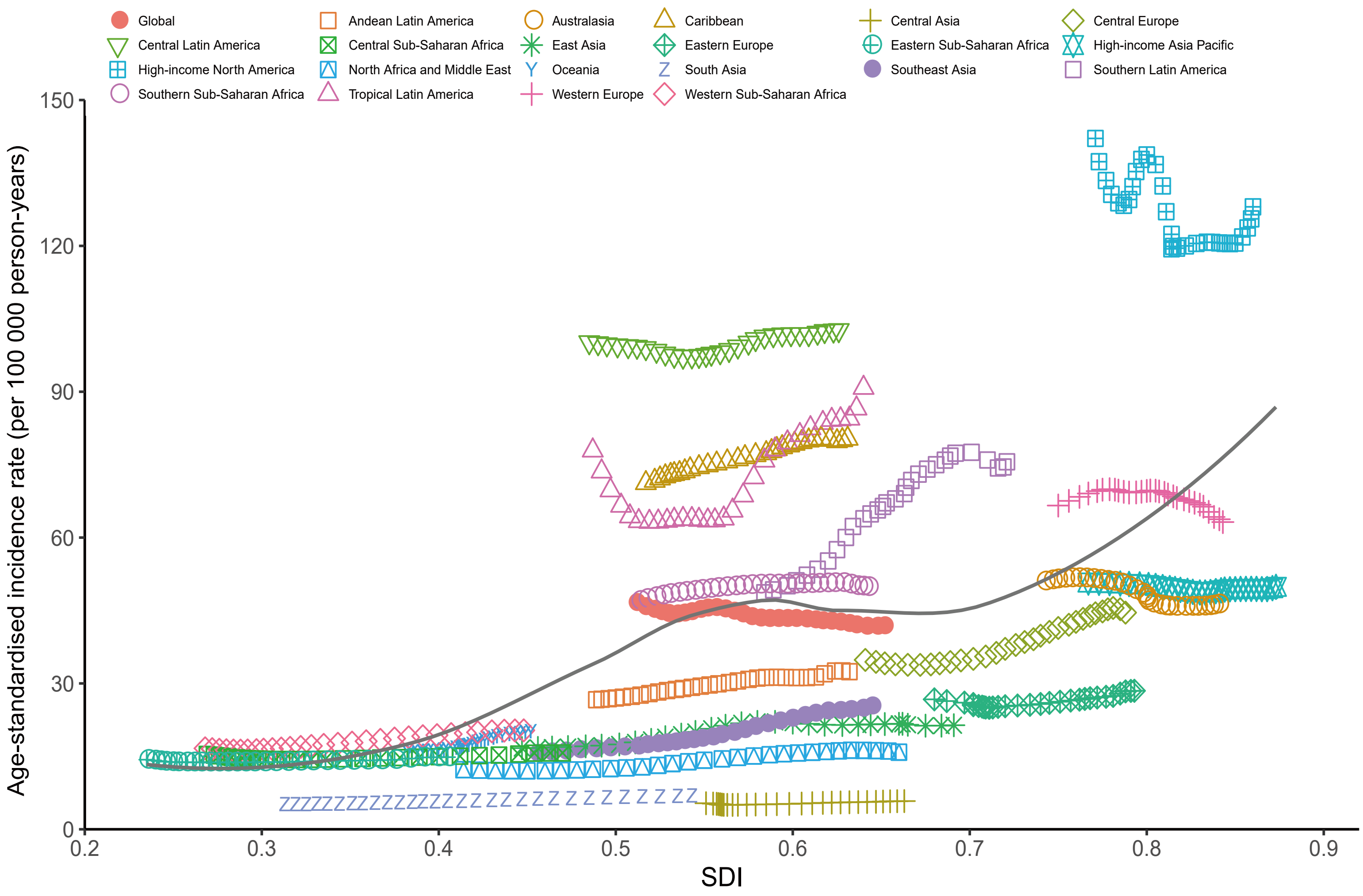

Figure S11: Association of age-standardized incidence rate due to decubitus ulcer and sociodemographic index (SDI) for 21 regions in the GBD study. Expected values based on Socio-demographic Index and disease rates in all locations are shown as the black line. Different points are plotted for each GBD region and show observed age-standardized incidence rates from 1990 to 2019.

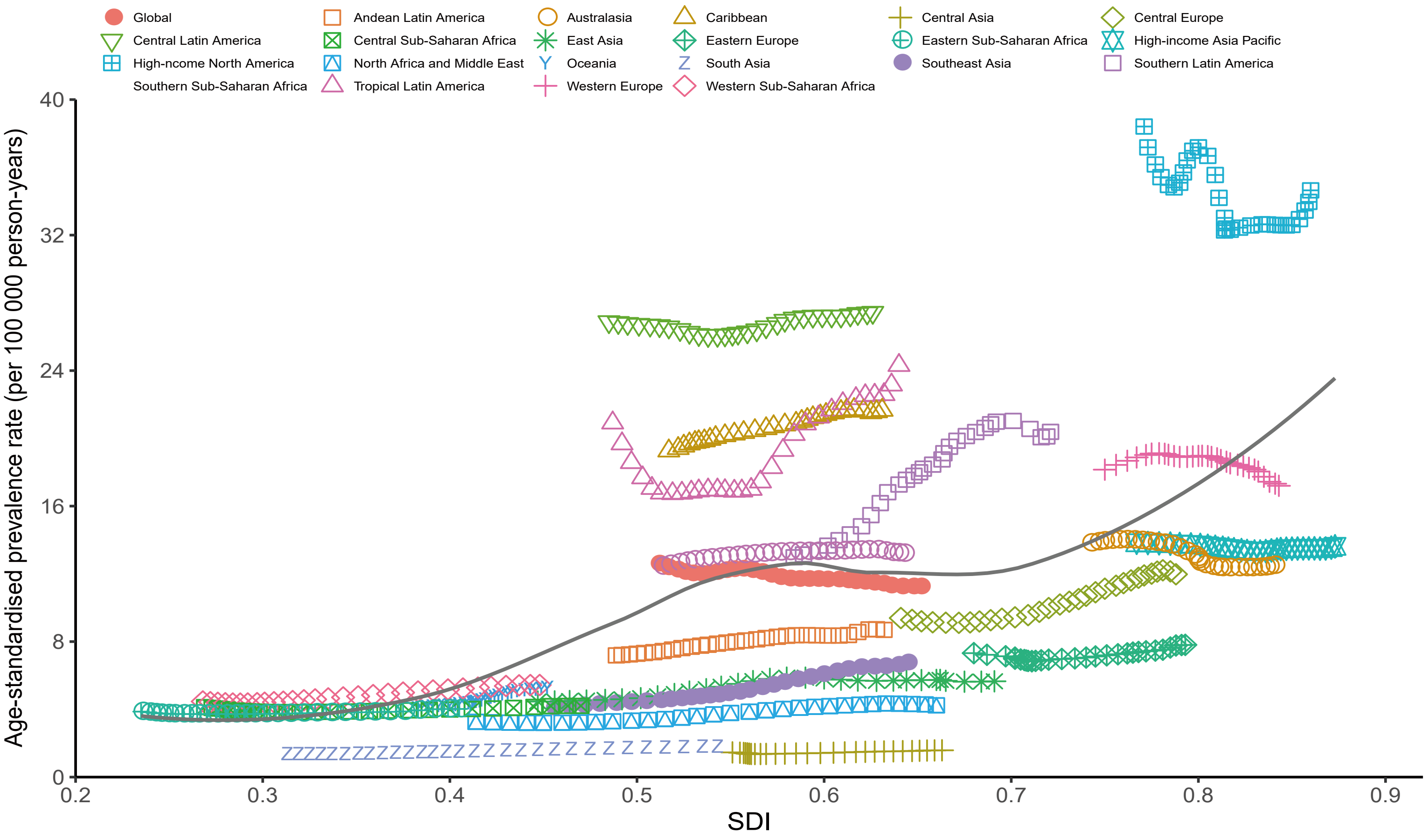

Figure S12: Association of age-standardized prevalence rate due to decubitus ulcer and sociodemographic index (SDI) for 21 regions in the GBD study. Expected values based on Socio-demographic Index and disease rates in all locations are shown as the black line. Different points are plotted for each GBD region and show observed age-standardized prevalence rates from 1990 to 2019.

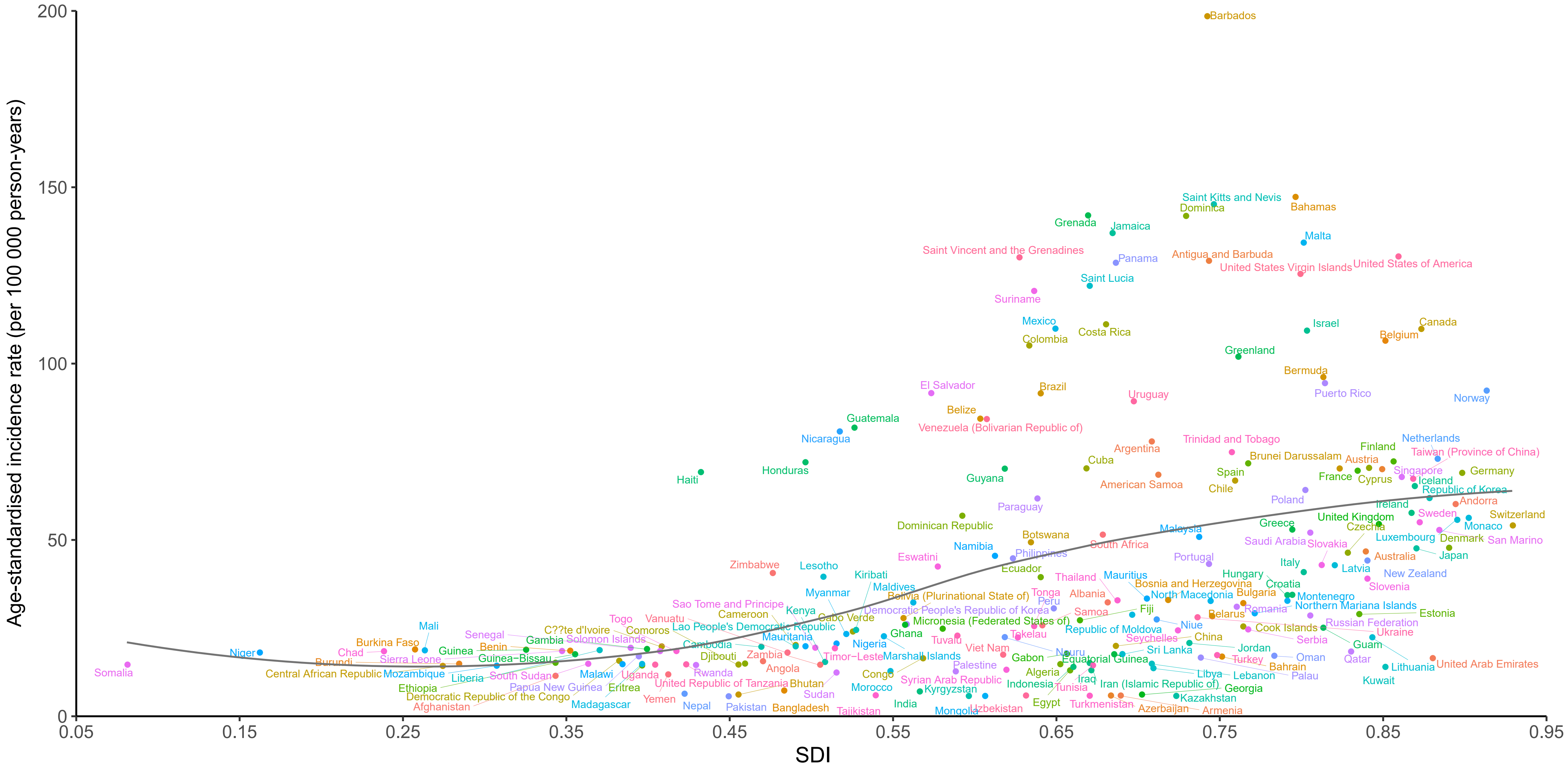

Figure S13: Association of age-standardized incidence rate due to decubitus ulcer and Sociodemographic Index for 204 countries and territories. Expected values based on Socio-demographic Index and disease rates in all locations are shown as the black line. Each point shows the observed incidence rate per country in 2019.

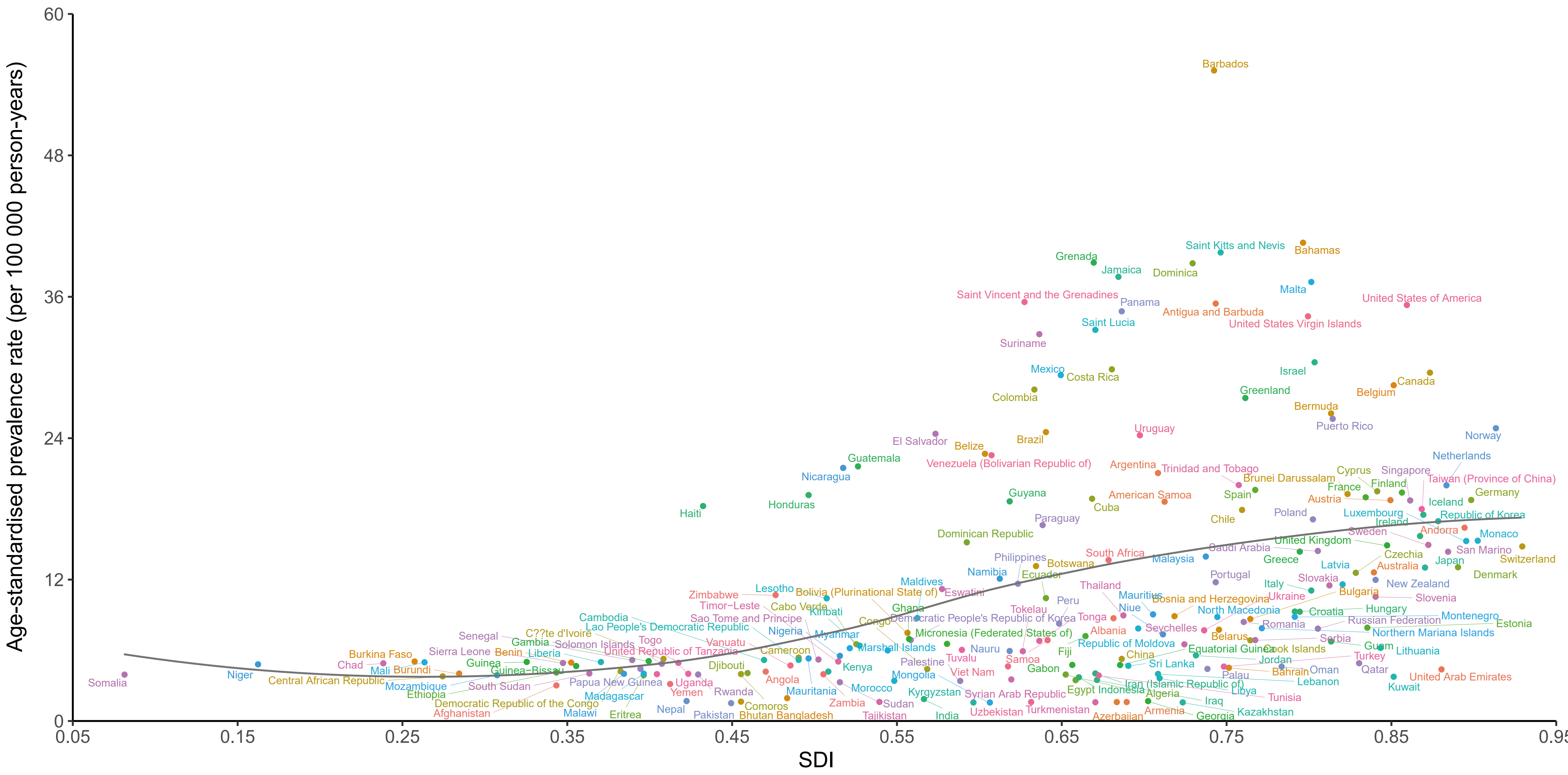

Figure S14: Association of age-standardized prevalence rate due to decubitus ulcer and Sociodemographic Index for 204 countries and territories. Expected values based on Socio-demographic Index and disease rates in all locations are shown as the black line. Each point shows the observed prevalence rate per country in 2019.
